# Endothelial STING and STAT1 mediate IFN-independent effects of IL-6 in an endotoxemia-induced model of shock

**DOI:** 10.1172/JCI189570

**Published:** 2025-09-16

**Authors:** Nina Martino, Erin K. Sanders, Ramon Bossardi Ramos, Iria Di John Portela, Fatma Awadalla, Shuhan Lu, Dareen Chuy, Neil Poddar, Mei Xing G. Zuo, Uma Balasubramanian, Peter A. Vincent, Pilar Alcaide, Alejandro P. Adam

**Affiliations:** 1 Department of Molecular and Cellular Physiology, Albany Medical College, Albany, New York, USA.; 2 Department of Immunology, Tufts University School of Medicine, Boston, Massachusetts, USA.; 3 Department of Ophthalmology, Albany Medical College, Albany, New York, USA.

**Keywords:** Inflammation, Vascular biology, Cell stress, Endothelial cells, Innate immunity

## Abstract

Severe systemic inflammatory reactions, including sepsis, often lead to shock, organ failure, and death, in part through an acute release of cytokines that promote vascular dysfunction. However, little is known about the vascular endothelial signaling pathways regulating the transcriptional profile in failing organs. Our work focused on signaling downstream of IL-6, due to its clinical importance as a biomarker for disease severity and predictor of mortality. Here, we show that loss of endothelial expression of the IL-6 pathway inhibitor SOCS3 promoted a type I IFN–like (IFNI-like) gene signature in response to endotoxemia in mouse kidneys and brains. In cultured primary human endothelial cells, IL-6 induced transient IFNI-like gene expression in a noncanonical, IFN-independent fashion. We further show that STAT3, which we had previously demonstrated to control IL-6–driven endothelial barrier function, was dispensable for this activity. Instead, IL-6 promoted a transient increase in cytosolic mitochondrial DNA and required STAT1, cGAS, STING, and IRF1, -3, and -4. Inhibition of this pathway in endothelial cell–specific STING-KO mice or global STAT1-KO mice led to reduced the severity of the response to acute endotoxemic challenge and prevented expression of an endotoxin-induced IFNI-like gene signature. These results suggest that permeability and DNA-sensing responses are driven by parallel pathways downstream of this cytokine, provide potential insights into the complex response to acute inflammatory responses, and offer the possibility of novel therapeutic strategies for independently controlling the intracellular responses to IL-6 in order to tailor the inflammatory response.

## Abstract 2

Shock induces severe vascular dysfunction. Here, we show that inflammatory cytokines drive an endothelial stress transcriptional response initiated by cytoplasmic DNA

## Graphical abstract

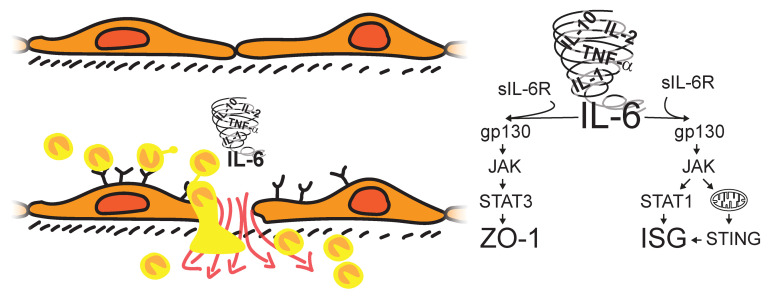

## Introduction

Severe systemic inflammatory reactions often lead to multiorgan dysfunction, in part through an acute release of cytokines — a process called cytokine storm (1, 2). These cytokines cause systemic vascular leak, coagulopathy, leukocyte adhesion, and vasodilation, resulting in severe hypotension, organ hypoperfusion, and ultimately organ failure and death (3–9). A better understanding of the specific roles of each cytokine in the vasculature is essential for the design of innovative therapeutics targeting systemic inflammation. Although a large number of cytokines are highly induced during a cytokine storm, circulating levels of the proinflammatory cytokine IL-6 correlate with disease severity (10–14) and are good predictors of shock mortality (15, 16). IL-6 receptor binding activates multiple parallel signaling pathways, including PI3K/Akt, MEK/ERK, and JAK/STAT3 (10, 11, 17). IL-6 signaling induces its own expression through a positive feedback loop that is associated with worse outcomes (10, 11, 18, 19) while also promoting a negative feedback loop by inducing expression of SOCS3, the primary inhibitor of JAK/STAT3 signaling (10, 11). How IL-6 promotes endothelial dysfunction and organ injury, however, remains poorly understood.

We previously reported that IL-6 signaling in cultured primary endothelial cells leads to an increase in endothelial permeability that requires STAT3 phosphorylation at Y705 and de novo protein synthesis (17). This signaling axis functions as a critical autocrine loop induced by LPS or TNF-α that is blocked by SOCS3 overexpression (20). The ability of SOCS3 to function as a signaling brake, however, is limited by very fast proteasome-mediated degradation (19). We further showed that endothelial cell–specific, tamoxifen-inducible SOCS3-knockout (SOCS3^iEKO^) mice display fast mortality after an LPS challenge, which is preceded by severe vasculopathy and kidney failure (19). Notably, organ damage and lethality were associated with a proinflammatory transcriptional profile, including a strong type I IFN–like (IFNI-like) response (19, 21, 22).

Canonical IFNI expression is induced by activation of stress response pathways, promoting activation of the cyclic GMP-AMP synthase (cGAS)/stimulator of IFN genes (STING) protein pathway. This leads to activation of TBK1, which induces phosphorylation and nuclear translocation of IFN regulatory factor (IRF) 3 and nuclear factor NF-κB. The latter 2 are crucial transcription factors that promote a rapid induction of IFNI (IFN-α and -β) expression (23, 24). In turn, activation of the IFN-α receptor (IFNAR) leads to JAK-mediated activation of STAT1 and STAT2, which together with IRF9 promote expression of IFN-stimulated genes (ISGs) (25). However, IFN signaling is complex (26), and recent reports showed IRF3-independent vasculopathy induced by STING activation (27, 28), thus suggesting that alternative pathways exist. It is well accepted that inflammatory stimuli such as LPS or TNF-α can promote IFNI expression (29–31). This is also true in endothelial cells, in which rapid IFNI expression was shown in response to several proinflammatory stimuli (32–34), and STING inhibitors prevent LPS- and TNF-α–induced expression of leukocyte receptors (35). In contrast, we showed that the IL-6–induced IFNI-like transcriptional response in vitro was associated with increased STAT1 and IRF activity (19, 21) but was surprisingly not associated with an increase in IFNI mRNA or protein expression (19). The mechanisms by which IL-6 can signal in endothelial cells to promote this IFNI-like transcriptional response remained unknown. Of note, the inflammatory phenotype of mice overexpressing IL-6 in the brain was largely not dependent on microglia STAT1 expression (36), suggesting that IL-6 dependency on STAT1 may depend on the cell type.

By performing translating ribosome affinity purification (TRAP) (37) and RNA sequencing (TRAP-Seq) on endotoxemic kidneys and brains to assess the whole-organ and the endothelial cell–specific transcriptional response in mice lacking or possessing SOCS3 in the endothelium, we demonstrated a strong endothelial cell–specific IFNI-like response in vivo. RNA-Seq from endothelial cell–enriched kidney samples demonstrated a crucial role for STING in this process. Mechanistically, we demonstrated that IL-6 induced direct expression of multiple ISGs that did not require an autocrine IFNI signaling loop or canonical STAT3 signaling. Instead, IL-6 led to transient release of mitochondrial DNA (mtDNA) and cGAS/STING-dependent gene expression that required parallel STAT1, IRF1, IRF3, and IRF4 activity, arguing for a noncanonical pathway downstream of IL-6 signaling promoting inflammatory gene expression.

## Results

### Acute endotoxemia leads to strong ISG expression in the kidney endothelium.

**Figure 1:**
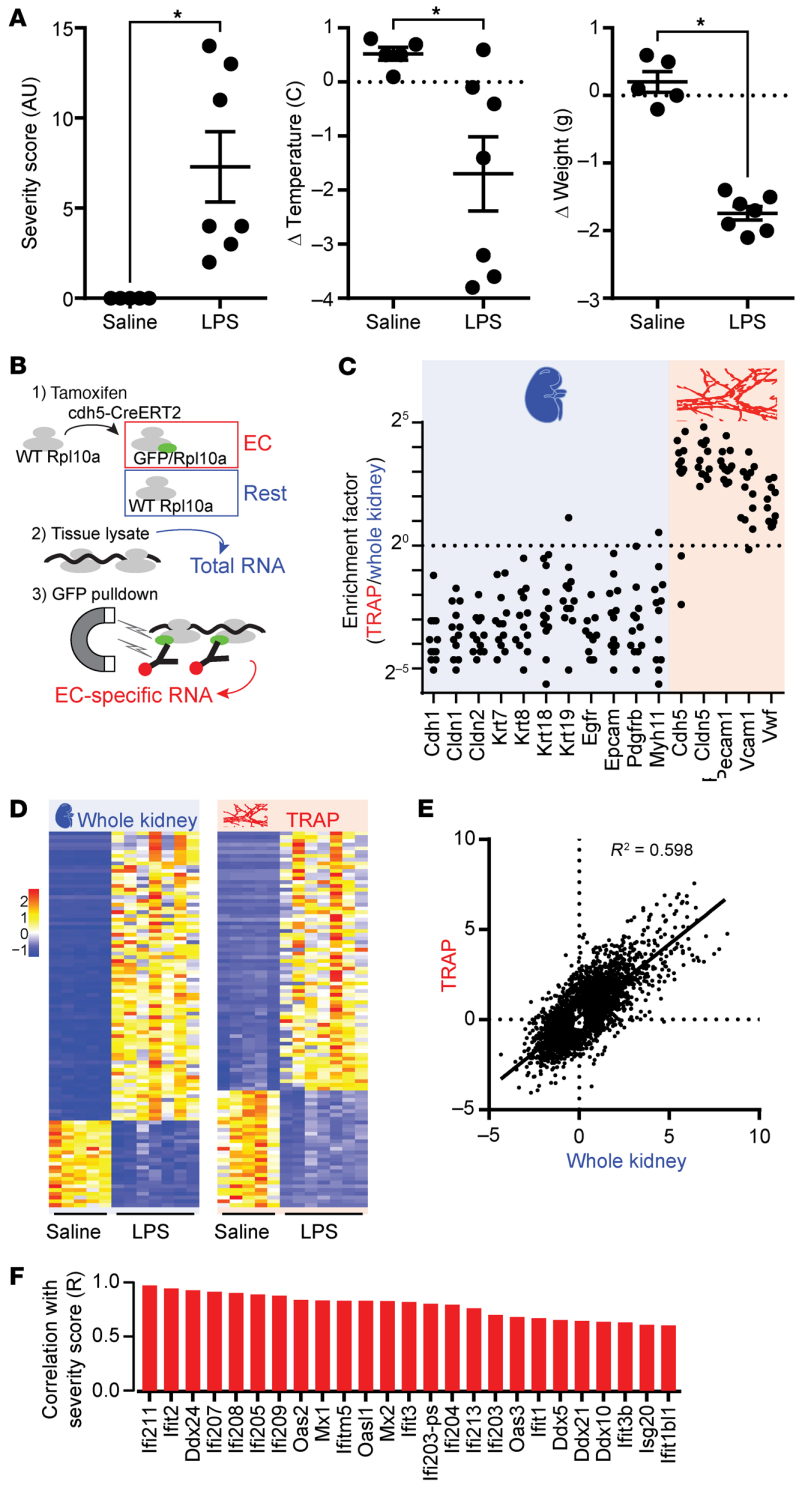
Endothelial transcriptional response in the kidney endothelium. (**A**) LPS induces physiological changes associated with a strong inflammatory reaction: increased severity, hypothermia, and an acute loss of body weight. *P < 0.05 (severity: Mann-Whitney *U* test; temperature: Welch’s *t* test; weight: unpaired *t* test). (**B**) Schematic of TRAP approach. EC, endothelial cell. (**C**) Ratio of normalized counts (TRAP-Seq over whole kidney RNA-Seq) obtained from the same organs. (**D**) Heatmap of the top 100 most significant changes in kidney expression for bulk RNA-Seq (left) and TRAP-Seq (right). Lookup table (LUT) corresponds to *z*-score. (**E**) Strong correlation between whole kidney and endothelial gene expression in response to LPS. (**F**) Endothelial expression of multiple IFN-regulated genes significantly correlates with disease severity (*P* < 0.05).

To determine whether the endothelium was responsible in part for the LPS-induced expression of ISGs, we performed endothelial cell–specific TRAP-Seq on mice challenged with a single LPS bolus. The TRAP approach allows for isolation of ribosome-bound mRNA to capture actively translated transcripts (37). Mice expressing the endothelial driver cdh5-CreERT2 (38) and floxed SOCS3 alleles (39) were described previously (19, 21, 22). To perform TRAP, the tdTomato reporter was replaced by an EGFP/Rpl10a transgene (37) on the same Rosa26 locus. EGFP/Rpl10a expression was induced in the endothelium by tamoxifen treatments at least 2 weeks prior to a challenge with 250 μg LPS. We previously reported that this dose led to 100% mortality in SOCS3^iEKO^ mice but was nonlethal in littermate WT SOCS3 mice (19) and led to substantial transcriptional changes in kidney and brain endothelium (21, 22). This challenge induced the expected increase in the severity score, hypothermia, and acute weight loss, demonstrating that this dose of LPS elicited a strong systemic reaction (Figure 1A). Mice were then euthanized and perfused with cycloheximide (CHX) prior to whole-kidney tissue homogenization. CHX perfusion is required to lock ribosomes in place for subsequent immunoprecipitation and isolation of ribosome-bound mRNA. After organ retrieval and tissue mincing, an aliquot was removed for total RNA extraction, and the rest was processed for TRAP pull-down. A diagram of the approach is shown in Figure 1B. RNA-Seq analysis of paired total and TRAP data from each mouse showed the high efficiency of the TRAP protocol, with high expression ratios for endothelial markers and low expression ratios for nonendothelial genes (Figure 1C). As expected, a challenge with LPS induced a strong transcriptional response in whole kidneys 15 hours later, with 1,040 genes upregulated at least 2-fold (adjusted *P* value < 0.05) and 1,309 genes downregulated at least 2-fold (adjusted *P* value < 0.05). The endothelium showed a similarly strong response (429 genes upregulated and 546 downregulated) (Figure 1D) that partially but significantly correlated with the total organ response (Figure 1E). Notably, the expression of many ISGs in the kidney endothelium highly correlated with overall severity score (Figure 1F).

### Loss of endothelial SOCS3 enhances ISG expression.

**Figure 2:**
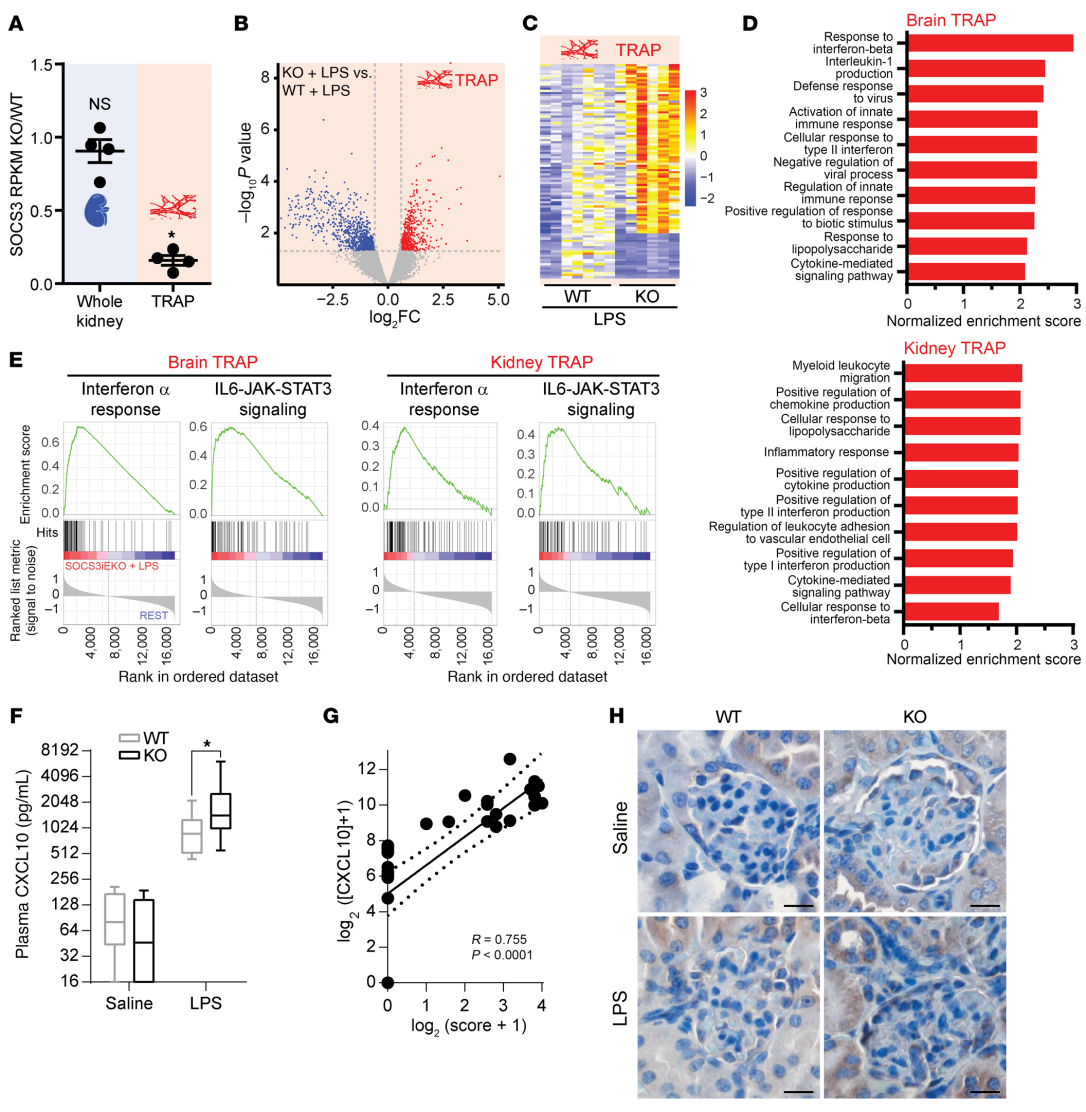
Loss of endothelial SOCS3 promotes a type I IFN–like response to LPS in the kidney and brain endothelium. (**A**) TRAP-Seq data demonstrating the specificity of the loss of SOCS3 expression in SOCS3^iEKO^ mice. (**B**) Volcano plot demonstrating a strong alteration of the endothelial transcriptional response to LPS. FC, fold change. (**C**) Heatmap of the most significant kidney transcriptional changes in response to LPS in SOCS3^iEKO^ mice compared with WT mice. (**D**) Normalized gene set enrichment analysis scores of kidney and brain TRAP-Seq comparing the LPS response in SOCS3^iEKO^ and WT mice. (**E**) Strong IFN-α signature in TRAP-Seq from LPS-treated SOCS3^iEKO^ mice. (**F**) Plasma CXCL10 levels are greatly increased by an LPS challenge in WT and further increased in SOCS3^iEKO^ mice. Box-and-whisker plot (line defines the median, the box extends from the 25th to 75th percentiles, and whiskers define the full range of value). *P < 0.05 (2-way ANOVA and Šidák’s post hoc test; *n* = 6–10). (**G**) Correlation between plasma CXCL10 levels and disease severity. Dotted lines represent 95% CI. (**H**) Immunohistochemistry of kidney to detect CXCL10. Scale bars: 20 μm. Representative of 3 independent cohorts.

We have previously shown fast mortality of SOCS3^iEKO^ mice upon similar LPS challenges that was associated with kidney failure and brain microvascular leak (19). Thus, we performed TRAP-Seq analysis to determine the response to LPS in mice lacking or not endothelial SOCS3. Figure 2A shows kidney TRAP-Seq counts of SOCS3 mRNA, demonstrating the efficacy and specificity of the endothelial knockout. Similar to recent findings in brain (22), loss of SOCS3 expression led to a dramatic increase in the number of genes responsive to LPS (975 genes with at least a 2-fold difference, adjusted *P* value < 0.05 in WT; 3,447 genes with at least a 2-fold difference, adjusted *P* value < 0.05 in SOCS3^iEKO^ mice), and SOCS3^iEKO^ mice showing a strong overall difference in gene expression in response to LPS compared with WT mice (Figure 2, B and C). Upstream pathway and transcription factor analysis demonstrated an expected increase in JAK/STAT activity in SOCS3^iEKO^ mice (Supplemental Figure 1; supplemental material available online with this article; https://doi.org/10.1172/JCI189570DS1). Consistent with a role for SOCS3 in limiting ISG expression, loss of SOCS3 further increased an IFN-like response in both kidney and brain endothelium (Figure 2, D and E).

To determine the systemic effects of IL-6–induced endothelial expression of CXCL10, we measured levels of this cytokine in WT and SOCS3^iEKO^ mice. The LPS challenge in WT mice induced a greater than 10-fold increase in circulating CXCL10 levels, which were elevated a further 2-fold in SOCS3^iEKO^ mice (Figure 2F). The levels of this cytokine highly correlated with severity score (Figure 2G). While CXCL10 protein expression was increased in endotoxemic SOCS3^iEKO^ mice (Figure 2H), the increase was much more subdued than for the RNA levels, suggesting that not all CXCL10 mRNA are translated in this setting or that the cytokine is quickly cleared from the affected tissue.

### Induction of ISGs by IL-6 in HUVECs does not require IFNAR1 expression.

**Figure 3:**
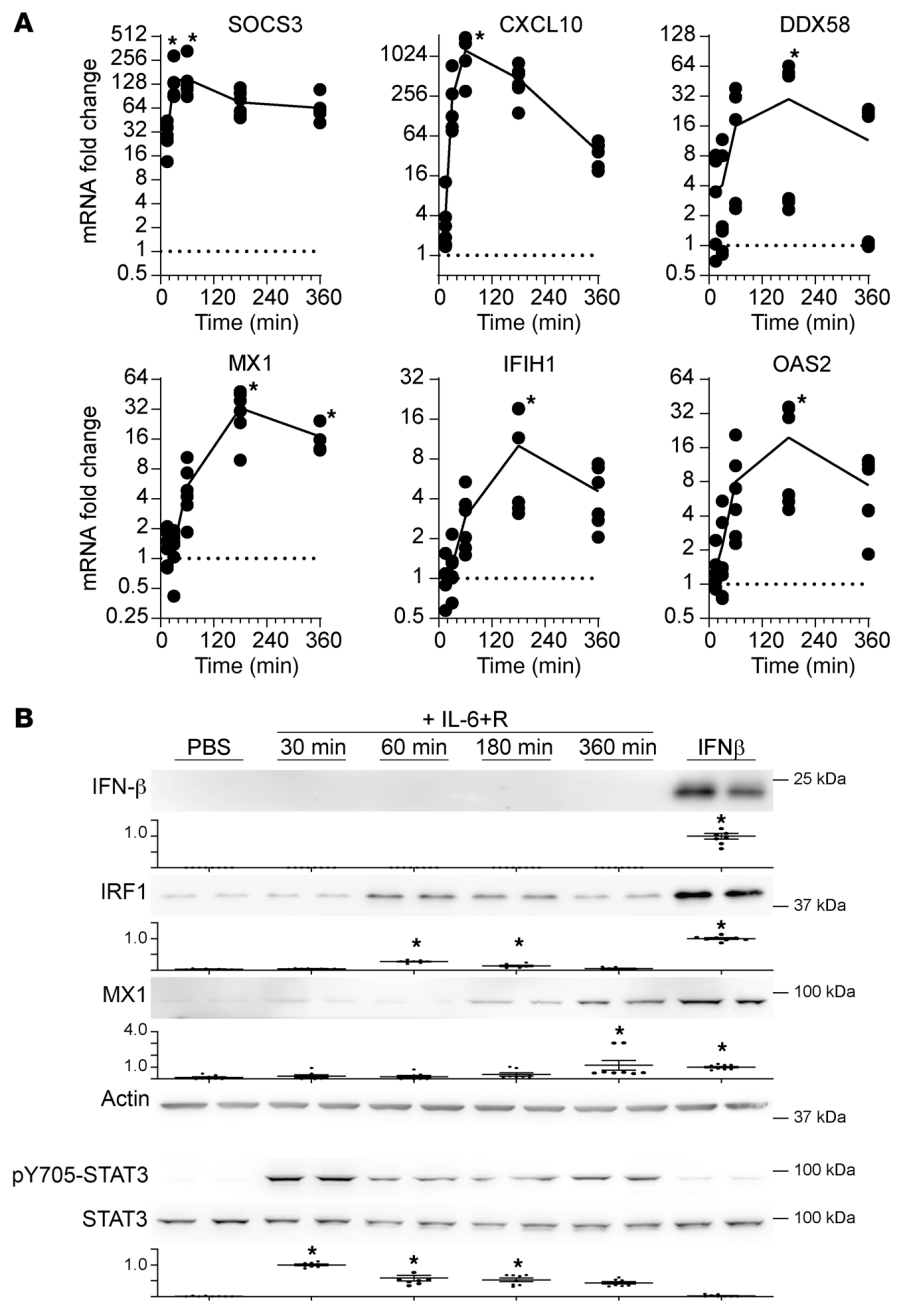
IL-6 promotes a type I IFN–like transcriptional response in HUVECs independently of IFNAR1 signaling. (**A**) RT-qPCR of HUVECs treated with or without 200 ng/mL recombinant human IL-6 and 100 ng/mL recombinant soluble IL-6Rα (IL-6+R). One-way ANOVA for all comparisons except IFIH1, in which a Welch’s ANOVA was performed due to unequal variance. (**B**) Western blotting against IFN-β, IRF1, or MX1 of confluent HUVECs treated with IL-6+R for the described times, or for 2 hours with IFN-β. STAT3 phosphorylation was used as positive control for IL-6 treatments. Upper panels: Representative blots of phosphorylated and total proteins. Lower panels: Normalized band intensity (phosphorylated over total) from 3 experiments. One-way ANOVA. *P < 0.05. Data representative of at least 3 independent experiments each performed in duplicate.

To better understand the mechanisms by which SOCS3 regulates ISG expression, we took advantage of our prior observations that activation of IL-6 signaling in cultured primary endothelial cells led to a transient increase in the expression of several IFN-associated genes, including *IRF1*, *IRF7*, and *MX1* (19). We treated HUVECs with a combination of IL-6 and its soluble receptor (herein referred to as IL-6+R) to confirm expression of increased *MX1* and to determine whether other ISGs were similarly induced by IL-6 signaling. We found that this treatment promoted a fast response to induce expression of *CXCL10*, *DDX58*, *MX1*, *IFIH1*, and *OAS2* (Figure 3A). The responses peaked between 1 and 3 hours after treatment, showing a slight delay compared with the response to the canonical STAT3 target SOCS3. This treatment led to increases in ISG protein expression with similarly fast kinetics. Expression of the transcription factor IRF1 peaked 1 hour after challenge, while expression of MX1 was detectable by 3 hours and further increased by 6 hours (Figure 3B). Consistent with its inhibitory activity, overexpression of a proteasome-resistant SOCS3 construct abrogated the IL-6–dependent induction of ISGs (Supplemental Figure 2). We chose to test the effect of this mutant, since we previously showed an enhancement of JAK/STAT signaling inhibition (19).

The increase of ISG expression by IL-6+R was then tested in the 2 other human endothelial cell types. In the human brain endothelial cell line HCMEC/D3, IL-6+R significantly upregulated expression of *CXCL10*, *DDX58*, *MX1*, and *IFIH1*, but surprisingly not *OAS2* (Supplemental Figure 3). The levels of increase were lower than in HUVECs, consistent with a reduced increase in SOCS3 and IL-6, suggesting a dampened overall response in this cell line. Notably, human primary glomerular endothelial cells showed an increase in *CXCL10* mRNA and IRF1 protein, but not other ISGs (Supplemental Figure 4). These results suggest that this response may depend in part on the origin of the endothelial cells and/or culture conditions.

**Figure 4:**
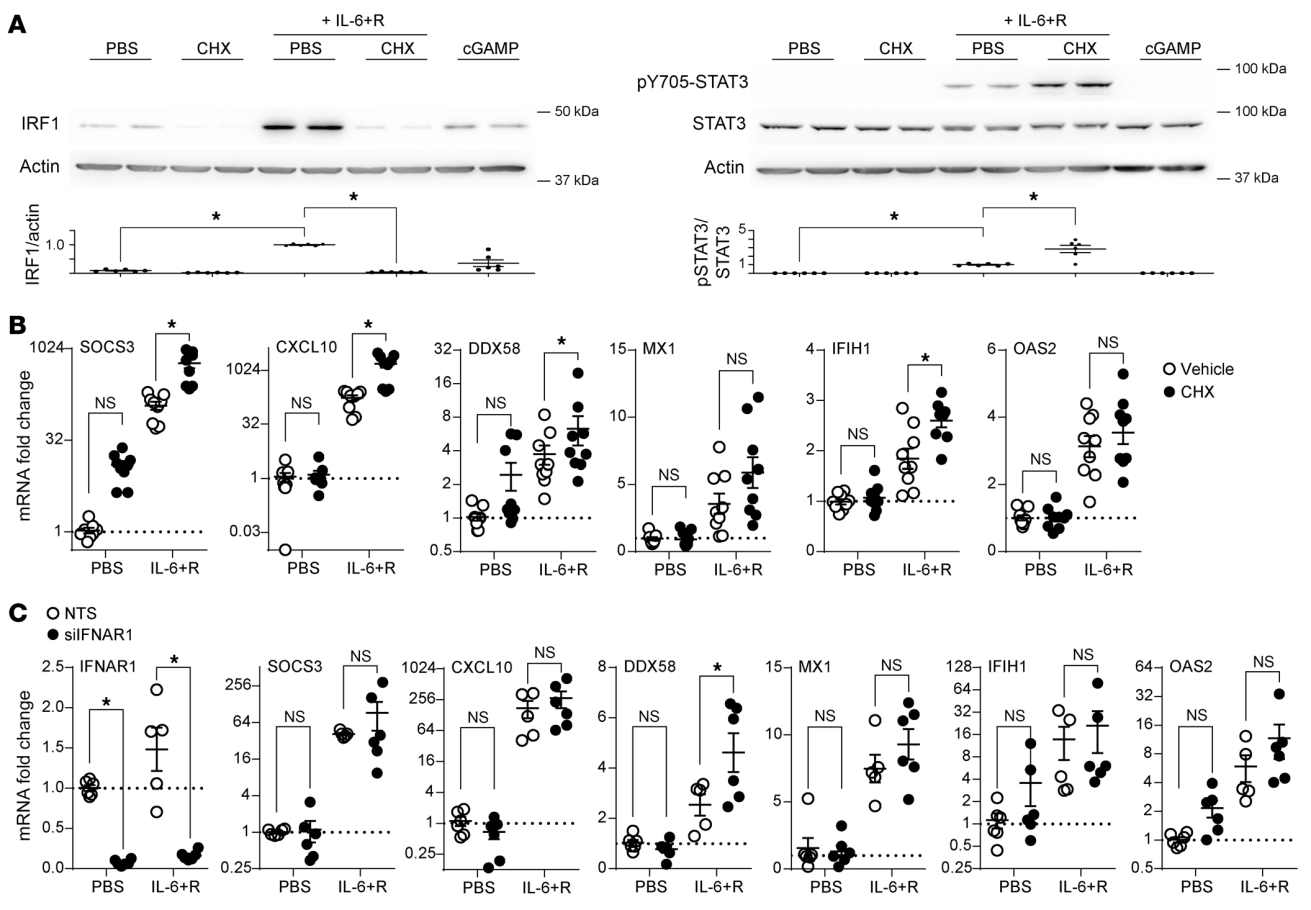
IL-6–induced ISG expression is independent of IFNAR1 signaling. (**A**) Western blotting against IRF1 or phosphorylated STAT3 of HUVECs treated for 2 hours with IL-6+R after pretreatment with or without CHX. Upper panels: Representative blots of phosphorylated and total proteins. Lower panels: Normalized band intensity (phosphorylated over total) from 3 experiments. (**B**) RT-qPCR of HUVECs treated as above. (**C**) RT-qPCR 2 hours after IL-6+R treatment in cells transfected with siRNA to knock down IFNAR1 (siIFNAR1) or with a nontargeting sequence control (NTS). Two-way ANOVA. *P < 0.05. Data representative of at least 3 independent experiments each performed in duplicate.

We have previously shown that an IL-6+R treatment in HUVECs does not lead to a detectable release of IFNs (19), and we were unable to detect IFN-β by Western blotting, at least within a 6-hour time frame (Figure 3B). These findings suggest that the increase in ISG expression was not due to an IFN autocrine loop, as described before for TNF-α (33, 34). This hypothesis was further supported by the quick response to IL-6+R shown in Figure 3. To directly assess whether IL-6+R signaling required de novo IFN protein synthesis for ISG expression, we pretreated cells with the ribosome inhibitor CHX prior to the IL-6+R challenge. This treatment completely abrogated production of IRF1 protein, demonstrating efficient inhibition of translation (Figure 4A). This was not due to an impairment of IL-6 signaling by CHX, because levels of STAT3 phosphorylation were not lowered by the ribosome inhibitor (Figure 4A). As shown in Figure 4B, CHX did not reduce the induction of ISG expression, and in many cases instead it exacerbated the response. Consistent with SOCS3 protein synthesis being required to dampen the IL-6 response, protein synthesis inhibition significantly increased both STAT3 phosphorylation (Figure 4A) and SOCS3 mRNA levels (Figure 4B). To determine whether a release of stored IFNs, which would be insensitive to CHX pretreatment, was responsible for ISG expression, we treated cells with IL-6+R after *IFNAR1* knockdown. As shown in Figure 4C, loss of *IFNAR1* expression did not prevent IL-6+R–induced ISG expression despite significant knockdown. Overall, these data suggested that IL-6 signaling does not require an autocrine IFN loop to induce a transient increase in ISG expression.

### Induction of ISGs by IL-6 in HUVECs requires STAT1, but not STAT3 expression.

**Figure 5:**
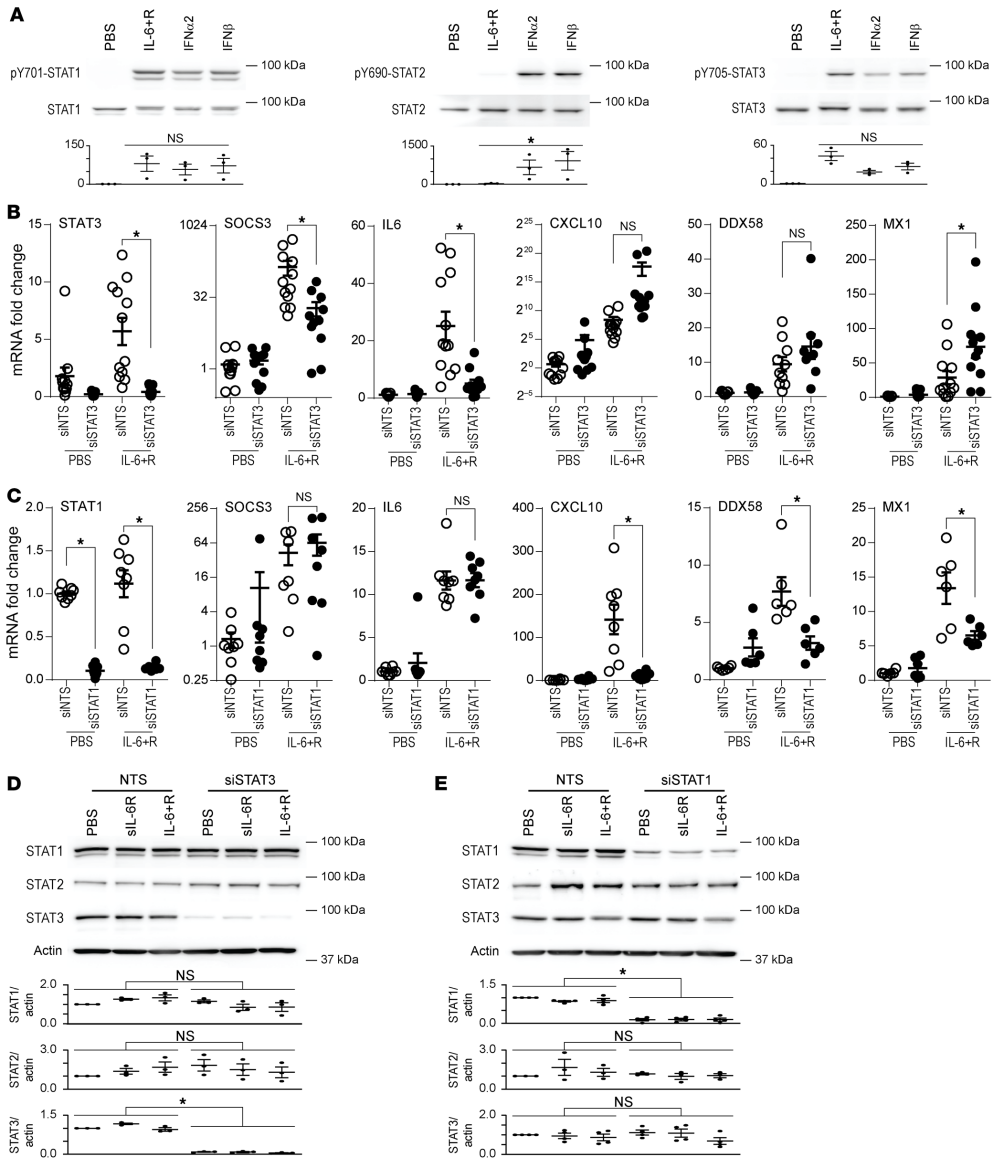
ISG expression by IL-6+R depends on STAT1 but not STAT3. (**A**) Western blotting of HUVEC lysates treated or not with IL-6+R, 2,000 U/mL IFN-α2, or 500 U/mL IFN-β. Upper panels: Representative blots of phosphorylated and total proteins. Images representative of 3 independent experiments. Lower panels: Normalized band intensity (phosphorylated over total) from 3 experiments. One-way ANOVA and Šidák’s post hoc test against IL-6+R. *P < 0.05. (**B**) RT-qPCR 2 hours after IL-6+R treatment in cells transfected with siRNA to knock down STAT3 (siSTAT3) or with a nontargeting sequence control (siNTS). (**C**) RT-qPCR of cells treated as in **B** but after siRNA-mediated STAT1 knockdown (siSTAT1). **B** and **C**: Two-way ANOVA and Šidák’s post hoc test. *P < 0.05. Data representative of at least 4 independent experiments each performed in duplicate. (**D**) HUVECs transfected with siRNAs against STAT3 or a nontargeting sequence were treated for 15 minutes with PBS, soluble IL-6 receptor (sIL-6R) or IL-6+R prior to cell lysis. (**E**) HUVECs treated as in **D** but after siRNA-mediated STAT1 knockdown. **D** and **E**: Representative Western blots (top panels) and band quantification and statistics of 3 independent experiments. *P < 0.05. Two-way ANOVA.

The levels of ISG expression as induced by 2 hours of IL-6+R treatment were significantly lower than those observed with direct treatment with recombinant IFN-α_2_ or IFN-β (Supplemental Figure 5). As expected, IFN treatment did not significantly induce SOCS3 expression within this time frame. The lower expression of ISGs in IL-6+R– versus IFN-treated cells could be explained by a lack of phosphorylation of STAT2 in response to IL-6+R when compared with IFN treatments (Figure 5A). Notably, STAT1 and STAT3 phosphorylation reached similar levels upon these treatments (Figure 5A). As expected, IL-6+R induced very fast STAT3 nuclear translocation. STAT3 remained nuclear, albeit at lower levels, for up to 6 hours (Supplemental Figure 6). While IFN-β induced nuclear translocation of both STAT1 and STAT2 with similarly fast kinetics, IL-6+R treatment led to nuclear translocation only of STAT1, but not STAT2 (Supplemental Figure 7), a finding consistent with the lack of STAT2 phosphorylation by IL-6+R.

We then sought to determine the relative importance of *STAT1* and *STAT3* in the ISG response to IL-6+R. As expected, and consistent with our own prior findings (17), *STAT3* knockdown completely abrogated the IL-6+R–induced expression of SOCS3 and significantly reduced *IL6* expression itself (Figure 5B). Efficient knockdown was confirmed by the significantly lower *STAT3* mRNA expression compared with control cells transfected with a nontargeting siRNA (siNTS). However, this knockdown was unable to block the increases in *CXCL10*, *DDX58*, or *MX1* (Figure 5B). In contrast, while *STAT1* knockdown did not limit IL-6+R–induced *SOCS3* or *IL6* expression, it significantly dampened the ISG response (Figure 5C). Transfection with this siRNA efficiently reduced *STAT1* mRNA levels (Figure 5C). Western blots demonstrating the efficacy and specificity of *STAT3* and *STAT1* knockdowns are shown in Figure 5, D and E.

### IL6-induced ISG expression involves mtDNA release and activation of cGAS and STING.

**Figure 6:**
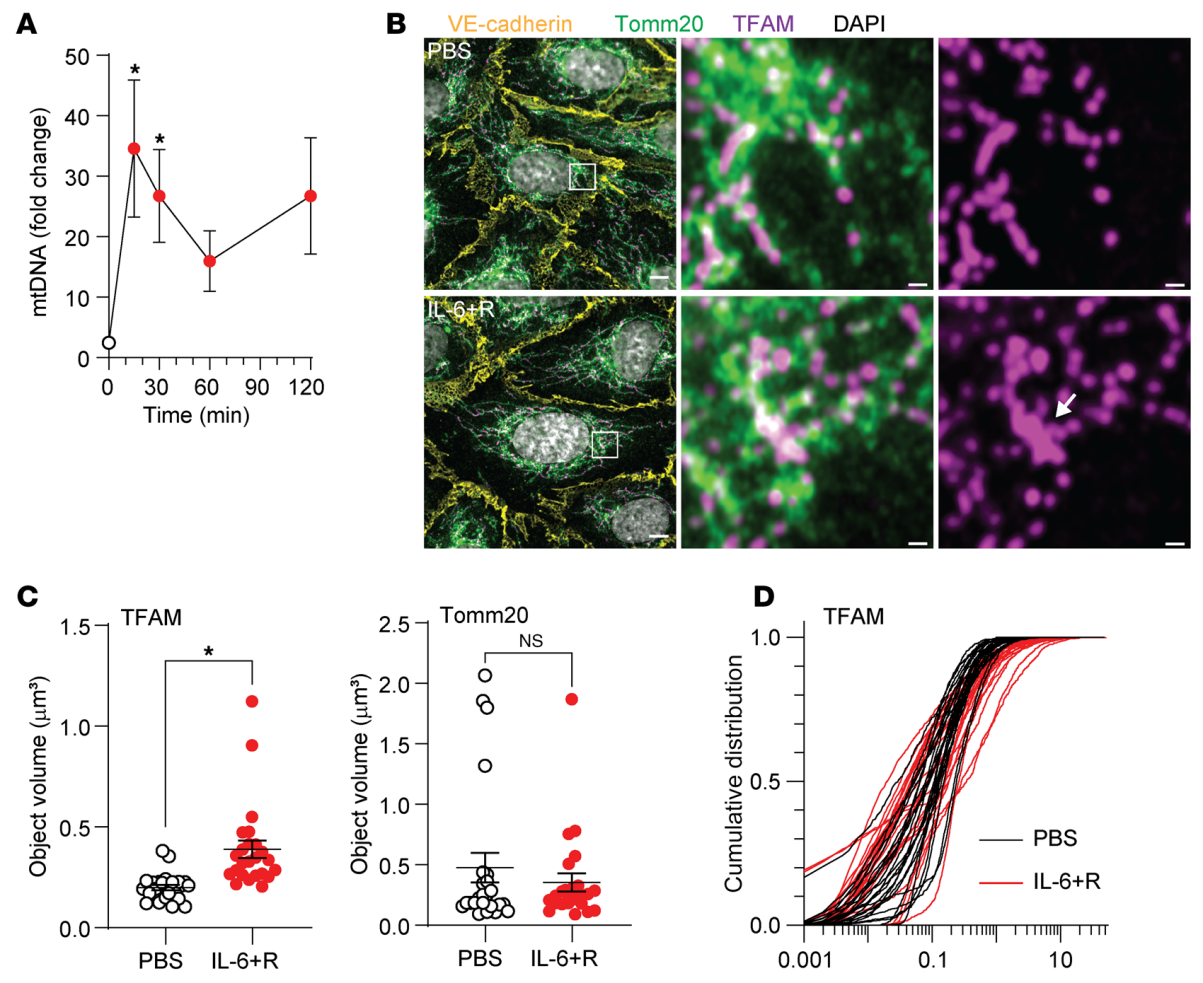
IL-6 leads to mtDNA release. (**A**) qPCR of mtDNA released into the cytoplasm in response to IL-6+R. One-way ANOVA. Data from 5 independent experiments. (**B**) Single section of 3D Airyscan imaging of the mitochondrial outer membrane marker Tomm20 and the mtDNA-binding protein TFAM as detected by indirect immunofluorescence of PBS- or IL-6+R–treated cells. The arrow points to a region of TFAM aggregation. (**C**). Images were rendered in Imaris to segment individual TFAM and Tomm20 objects. Plotted is the average size of each object within an image field. *P < 0.05 by unpaired *t* test. (**D**) Cumulative distribution of all objects for each imaging field. Each line corresponds to an average as shown in **C**. Data from 3 independent experiments; 8 imaging fields per condition, per experiment.

STAT1 activation alone is not described to promote an IFNI-like transcriptional signature. Thus, we hypothesized that a parallel pathway may be activated by IL-6+R. Mitochondrial damage mediates many responses to proinflammatory signaling in the endothelium. We performed mild lysis and centrifugation to isolate the cytoplasmic fraction, followed by quantitative PCR, and found that IL-6+R induced a transient but significant release of mtDNA into the cytoplasm (Figure 6A). This release peaked at 15 minutes after treatment and began resolving after that, without causing apoptosis or other forms of cell death. This transient nature of the mtDNA release is consistent with the fast kinetics of mRNA and protein expression after an IL-6+R challenge (Figure 3). Indirect immunofluorescence and super-resolution imaging (Zeiss Airyscan; Figure 6B) showed that this mitochondrial dysfunction was associated with a marked aggregation of the mitochondrial transcription factor A TFAM (40) within mitochondria, as labeled with the outer-membrane marker Tomm20. 3D reconstruction and rendering using Imaris allowed us to quantify the size of each object. Consistent with the aggregation shown in Figure 6B, we measured significantly larger sizes of TFAM objects in IL-6+R–treated cells (Figure 6, C and D). Together, these findings suggest that IL-6 leads to fast, self-limited mitochondrial dysfunction and DNA release that could further promote ISG expression.

**Figure 7:**
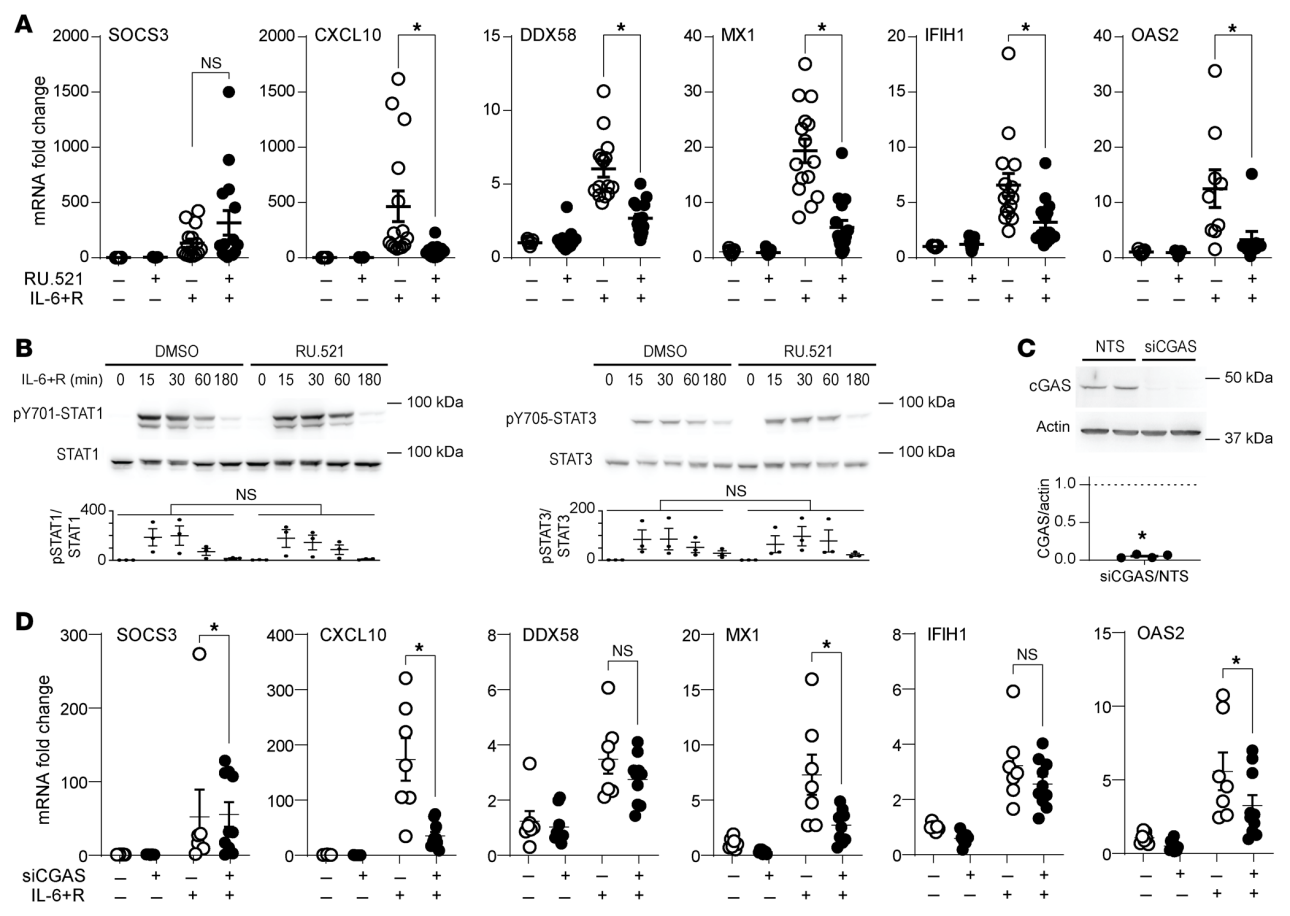
ISG expression in HUVECs in response to IL-6+R requires cGAS signaling. (**A**) RT-qPCR of HUVECs treated for 30 minutes with the cGAS inhibitor RU.521 prior to treatment for 2 hours with or without IL-6+R. (**B**) Western blotting of cells treated with or without RU.521 and IL-6+R to determine the phosphorylation levels of STAT1 and STAT3. (**C**) Western blotting of cells treated with or without siRNA to knock down cGAS (siCGAS) to measure knockdown efficiency. Upper panels: Representative Western blot. Lower panels: Fold expression versus NTS. Single-sample *t* test. (**D**) RT-qPCR of HUVECs transfected with NTS or CGAS siRNA prior to treatment for 2 hours with or without IL-6+R. (**B** and **D**) Two-way ANOVA and Šidák’s post hoc test. *P < 0.05. Data representative of at least 3 independent experiments.

cGAS is a major cytoplasmic DNA sensor, leading to STING-dependent type I IFN signaling. Thus, we sought to determine the involvement of cGAS and STING in this pathway. Inhibition of cGAS activity by the inhibitor RU.521 significantly dampened ISG induction by IL-6+R (Figure 7A), without reducing either STAT1 or STAT3 activation (Figure 7B). Depletion of *CGAS* by siRNA-mediated knockdown (Figure 7C) also limited IL-6+R–induced ISG expression, although not for all genes assayed (Figure 7D).

**Figure 8:**
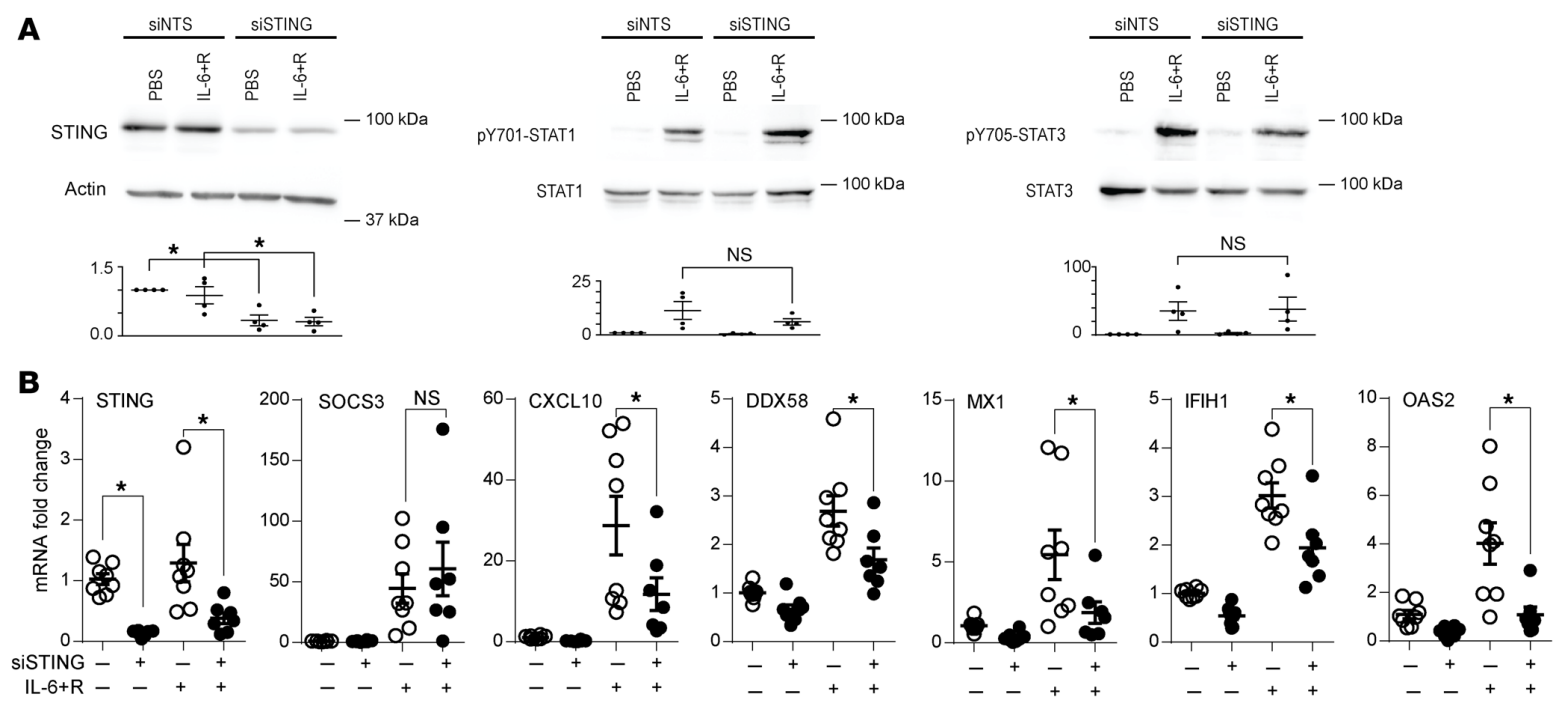
ISG expression in HUVECs in response to IL-6+R requires STING signaling. (**A**) Western blotting of cells treated with or without siRNA to knock down STING (siSTING) and IL-6+R to measure phosphorylation levels of STAT1 and STAT3. (**B**) RT-qPCR of HUVECs transfected with NTS or STING siRNA prior to treatment for 2 hours with or without IL-6+R. Two-way ANOVA and Šidák’s post hoc test. *P < 0.05. Data representative of at least 3 independent experiments.

Consistent with these findings, knockdown of the cGAS downstream mediator *STING* did not alter STAT1 or STAT3 phosphorylation (Figure 8A), but abrogated IL-6+R–induced ISG expression (Figure 8B). *STING* knockdown did not alter IL-6+R–induced SOCS3 expression (Figure 8B), suggesting a role independent of STAT3.

### IRF1 and IRF3 mediate transcriptional changes downstream of IL-6 signaling.

**Figure 9:**
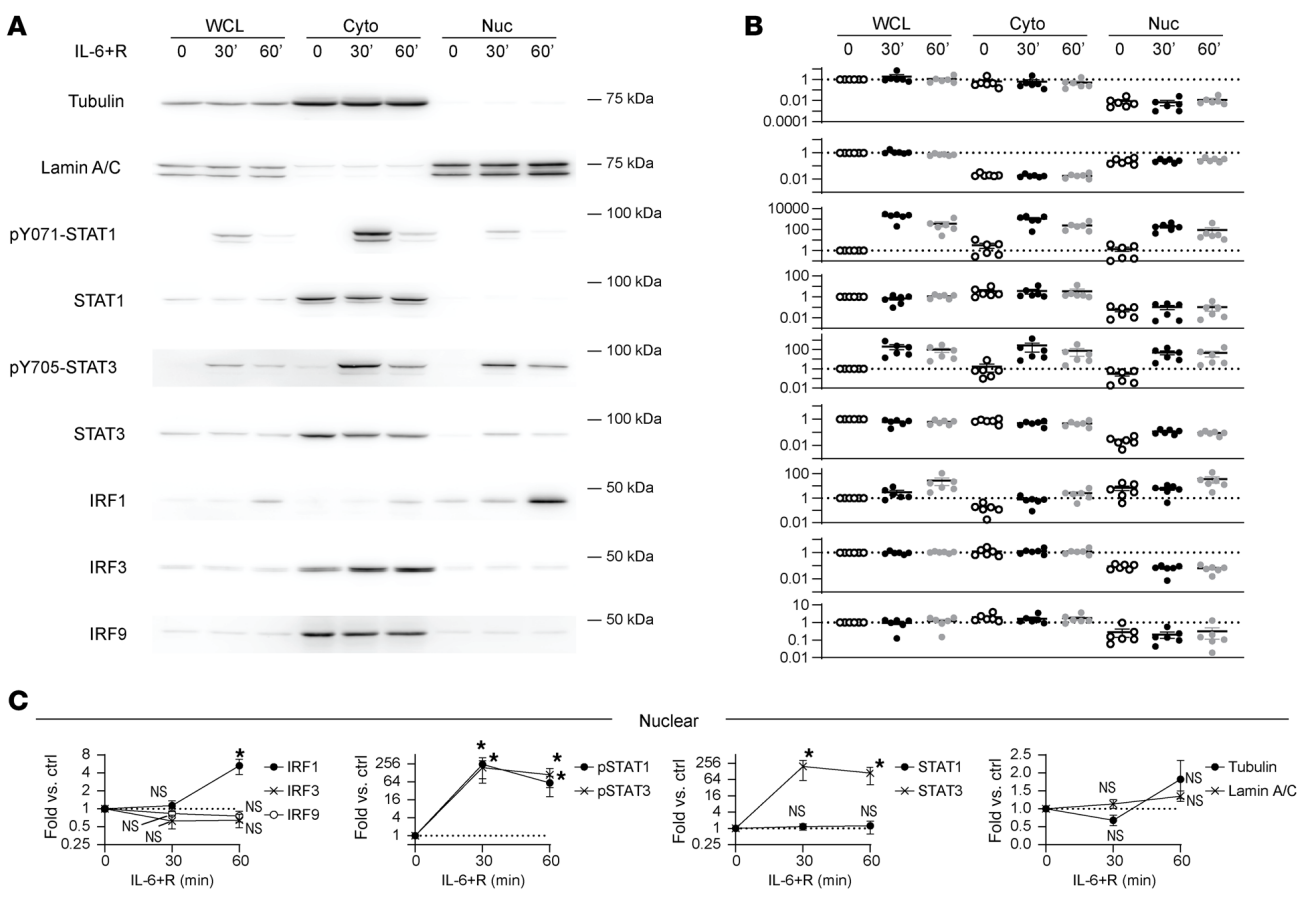
IL-6+R–induced nuclear translocation of IRF1 and phosphorylated STAT1 and STAT3. Subcellular fractionation of HUVECs treated or not with IL-6+R for either 30 minutes or 1 hour. (**A**) Representative Western blotting for whole cell lysates (WCL), cytoplasmic fractions (Cyto), or nuclear fractions (Nuc). Tubulin was used as a cytoplasmic marker, and lamin A/C was used as nuclear marker. (**B**) Densitometry analysis. Data expressed as relative intensity compared with control WCL. (**C**) Densitometry data of nuclear bands expressed as a ratio to control nuclear protein. Three independent experiments performed in duplicate each.

STING promotes activation of IRF proteins, and IRFs can also mediate IFN-independent responses. An analysis of all publicly available ChIP data for the ISGs described above, obtained from ChIP-Atlas: Peak Browser (41), suggested that direct binding of multiple IRFs occurred near the promoter regions in close proximity to, or overlapping with, STAT-binding sites (Supplemental Figure 8). Thus, we sought to determine whether IRF transcription factors were involved in the IFNI-like transcriptional response downstream of IL-6. Although IRF9 has been shown to mediate the transcriptional response to IFNs, indirect immunofluorescence did not detect significant nuclear translocation of IRF9 upon IL-6+R challenge, suggesting that this IRF may not be involved in this particular response (Supplemental Figure 9). Lack of IRF9 nuclear translocation was confirmed by subcellular fractionation and Western blotting (Figure 9A). Western blot analysis was unable to detect expression of IRF5, -6, -7, and -8 (data not shown).

**Figure 10:**
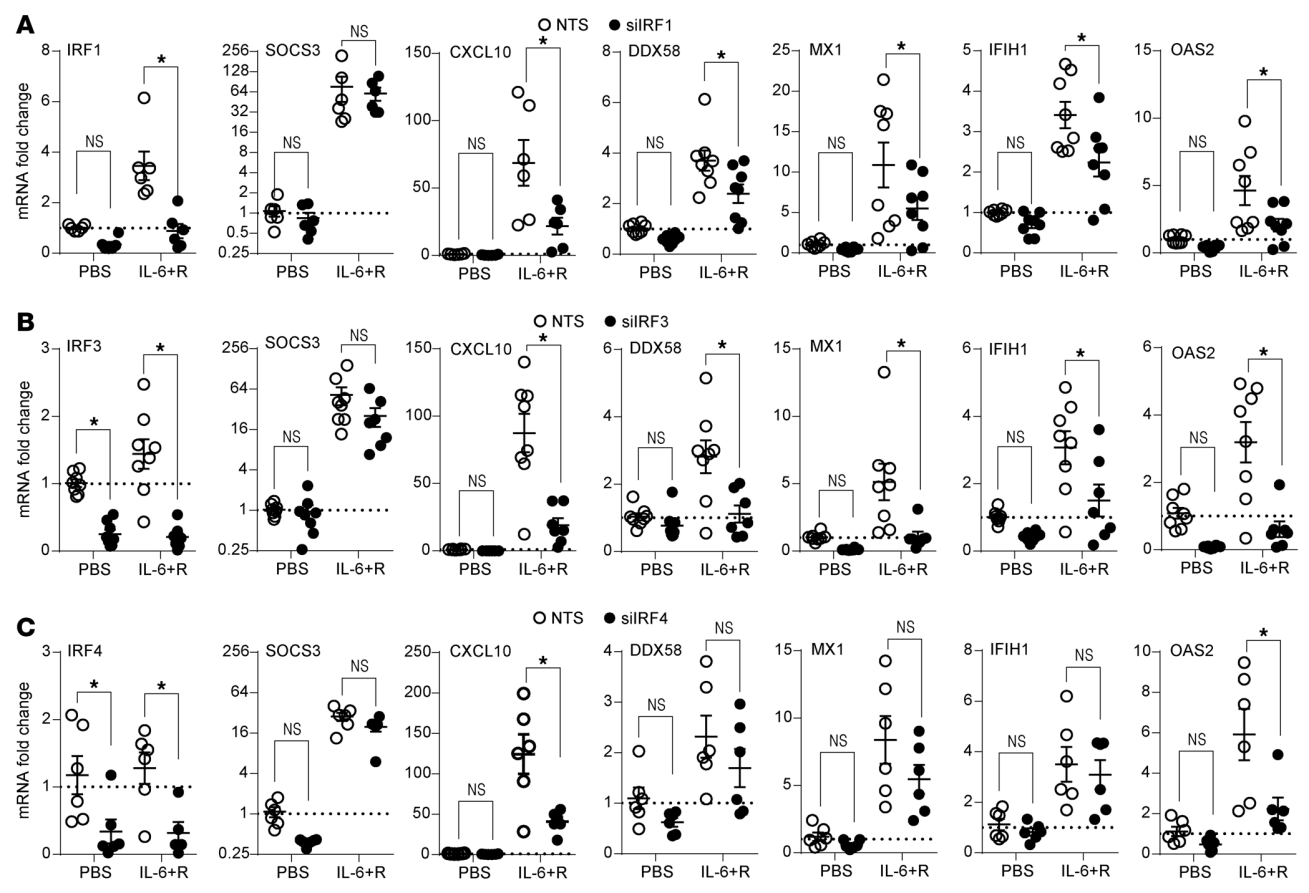
ISG expression in HUVECs in response to IL-6+R requires expression of IRF1, IRF3, and IRF4. (**A**) RT-qPCR of HUVECs transfected with NTS, IRF1 (**A**), IRF3 (**B**), or IRF4 (**C**) siRNA prior to treatment for 2 hours with or without IL-6+R. Two-way ANOVA and Šidák’s post hoc test. *P < 0.05. Data representative of at least 3 independent experiments.

We thus focused our attention on a potential role for IRFs 1–4. IRF1 showed a constitutively nuclear signal and a substantial additional translocation to the nucleus 1 hour after IL-6+R challenge (Figure 9), consistent with its increase in protein expression, whereas IRF3 showed a small but constant nuclear signal. Knockdown of both *IRF1* (Figure 10A) and *IRF3* (Figure 10B) significantly reduced expression of multiple ISGs upon IL-6 signaling, whereas *IRF4* knockdown (Figure 10C) led to a significant reduction of only a subset of genes. In contrast, *IRF2* knockdown did not block induction of any of these ISGs (Supplemental Figure 10). Notably, while we detected significant increases in phosphorylated STAT1 and STAT3, we found that only nuclear STAT3 levels significantly increased with IL-6+R (Figure 9), suggesting that only a small portion of phosphorylated STAT1 is sufficient to drive the observed gene expression.

**Figure 11:**
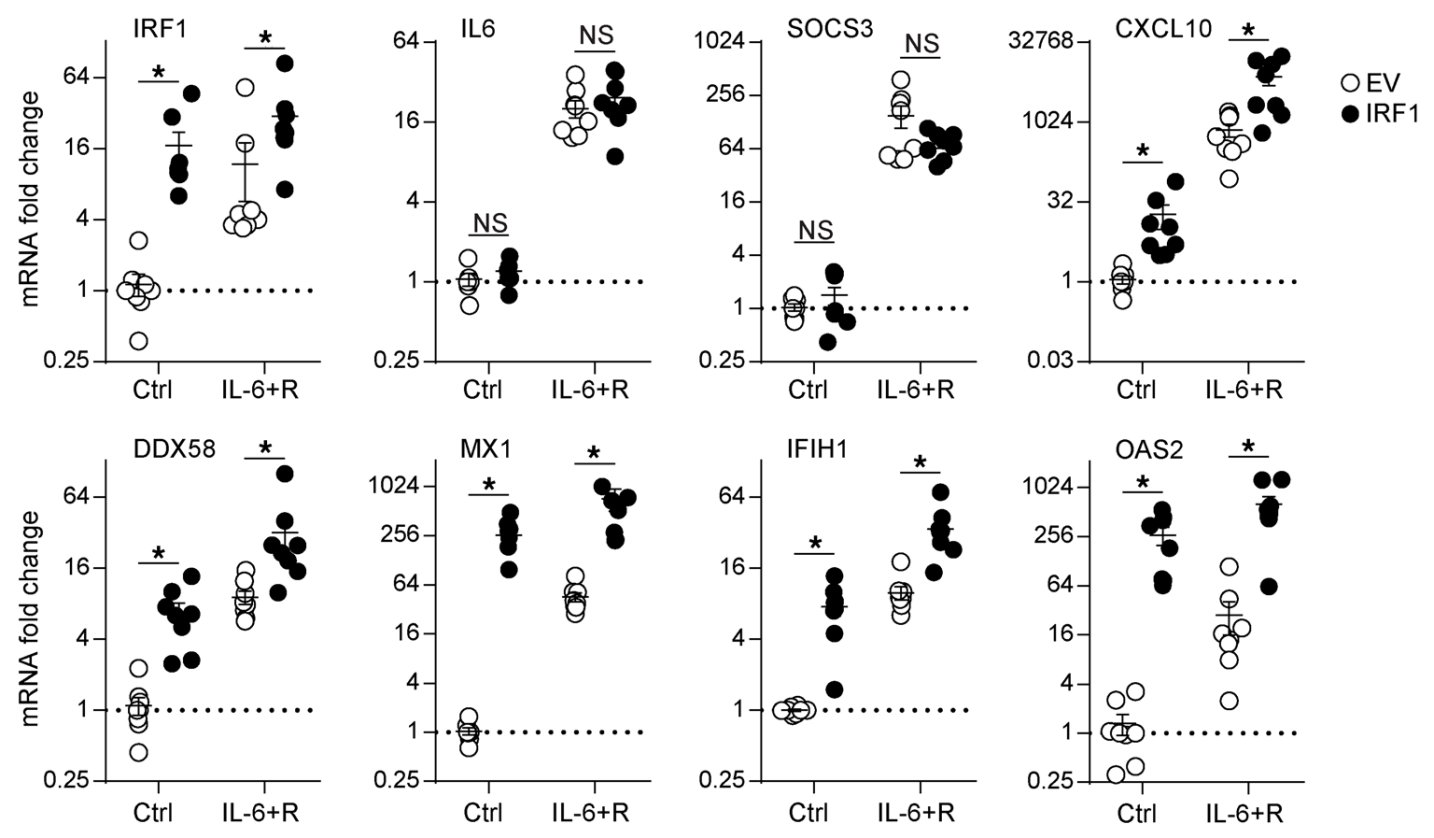
IRF1 overexpression in HUVECs promotes ISG expression. HUVECs were transduced with a lentivirus to overexpress IRF1 or with an empty vector control (EV). RT-qPCR of cells treated for 2 hours with or without IL-6+R. Two-way ANOVA and Šidák’s post hoc test. *P < 0.05. Data representative of 3 independent experiments.

Moreover, lentiviral *IRF1* overexpression led to a significant increase in ISG expression on its own and further increased the levels of expression induced by IL-6+R, suggesting that *IRF1* activation may be sufficient to drive ISG expression in endothelial cells. This effect was specific to ISG expression, because *IRF1* overexpression did not alter *IL6* or *SOCS3* mRNA levels alone or in response to IL-6+R (Figure 11).

### Loss of endothelial STING limits the severity of the LPS challenge.

**Figure 12:**
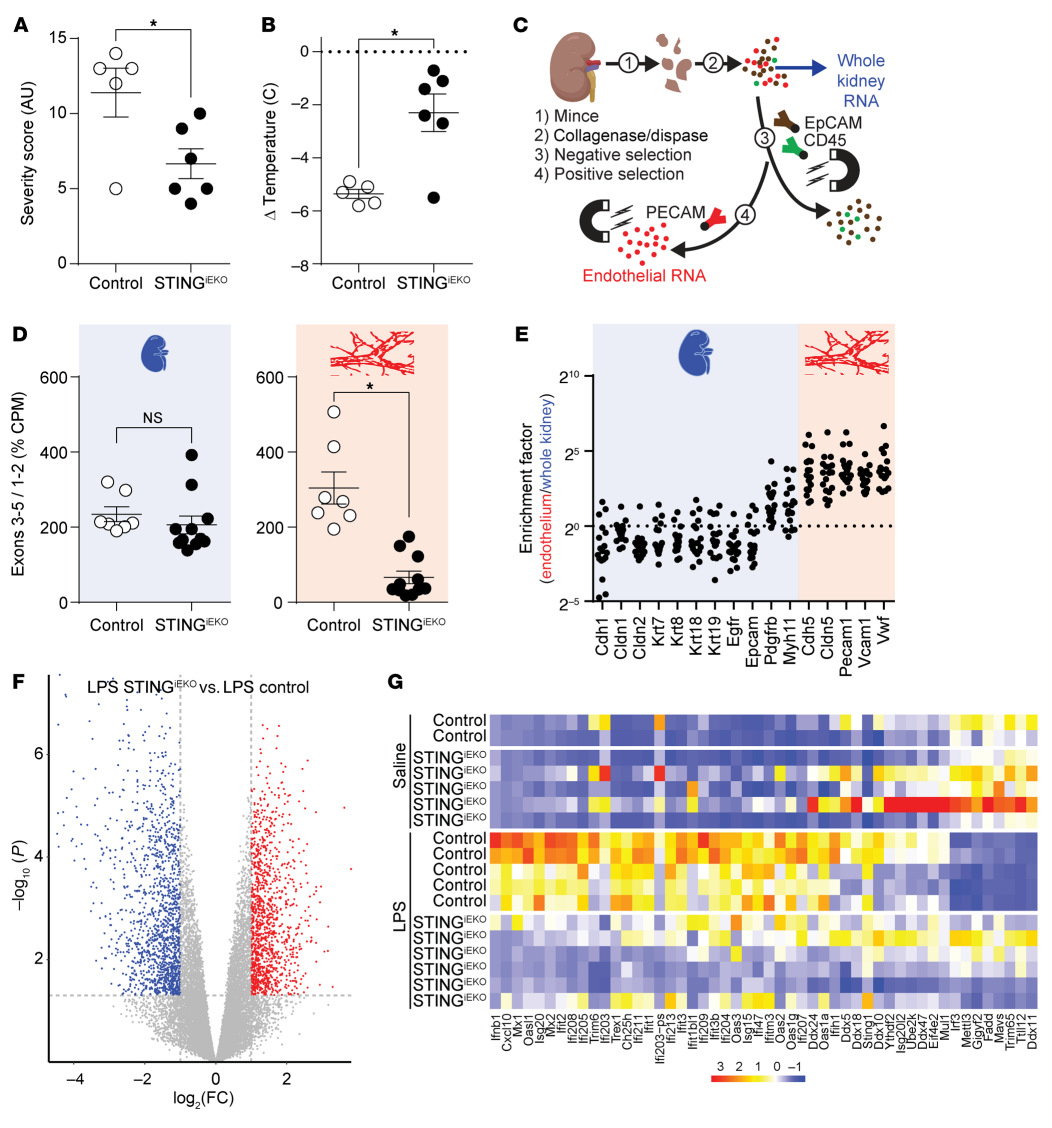
Endothelial STING is required for the kidney type I IFN–like response to LPS. STING^iEKO^ mice were challenged with an intraperitoneal bolus of LPS for 15 hours. Disease severity (**A**) and body temperature (**B**) were measured prior to euthanasia. Immediately afterward, kidneys were processed for RNA isolation from whole tissue and from an endothelial cell–enriched fraction following a 2-step magnetic bead isolation procedure (**C**). (**D**) RNA-Seq counts demonstrating loss of exons 3–5 in the endothelium of STING^iEKO^ mice. (**E**) Ratio of endothelial cell–enriched over whole-kidney RNA-Seq normalized counts obtained from the same organs. (**F**) Volcano plot demonstrating a strong alteration of the endothelial transcriptional response to LPS by loss of STING. (**G**) Heatmap of kidney endothelium expression of ISGs. LUT corresponds to *z*-scores. **A**, **B**, and **D**: *P < 0.05 (severity: Mann Whitney *U* test; temperature: unpaired *t* test; STING expression: unpaired *t* test (whole kidney) or Welch’s *t* test (TRAP-Seq).

To determine whether this pathway was involved in the systemic response to LPS, we performed a challenge in mice lacking or not lacking STING in endothelial cells. Mice carrying both cdh5-CreERT2 and homozygous floxed *Sting1* (coding for STING) were treated for 3 days with tamoxifen (STING^iEKO^) or vehicle (control) 2 weeks prior to LPS injections. STING^iEKO^ mice showed reduced overall disease severity (Figure 12A) and hypothermia (Figure 12B) in response to LPS when compared with control mice. To determine whether *Sting* mediated ISG expression in endothelial cells, we obtained kidneys from LPS- or saline-treated mice and performed RNA-Seq on an endothelial cell–enriched population derived from single-cell suspensions using a 2-step approach including negative selection of non–endothelial cells using EpCAM- and CD45-labeled magnetic beads, followed by PECAM-labeled bead–mediated positive selection (Figure 12C). An aliquot of single cells was obtained prior to endothelial cell enrichment for whole-kidney bulk RNA-Seq. First, we confirmed successful excision of exons 3–5 of the *Sting1* gene in the endothelium but not in the whole kidney from STING^iEKO^ mice (Figure 12D). Endothelial cell enrichment was confirmed by determining the ratio of expression of endothelial cell markers in the enriched population to those in the total kidney (Figure 12E). Of note, in contrast to the efficacy of the TRAP approach (Figure 1), the magnetic bead enrichment approach was efficient in removing epithelial cells, but mural cells (pericytes and smooth muscle cells) appeared to remain as contaminants (Figure 12E). Consistent with the reduction in disease severity, the response to LPS was dramatically altered in STING^iEKO^ mice compared with controls (Figure 12F). Most notably, loss of endothelial *Sting1* greatly diminished the LPS-induced increase in ISG expression (Figure 12G), demonstrating a crucial role for STING in the endothelial transcriptional response to shock.

### Loss of STAT1 expression reduces LPS severity and endothelial ISG expression.

**Figure 13:**
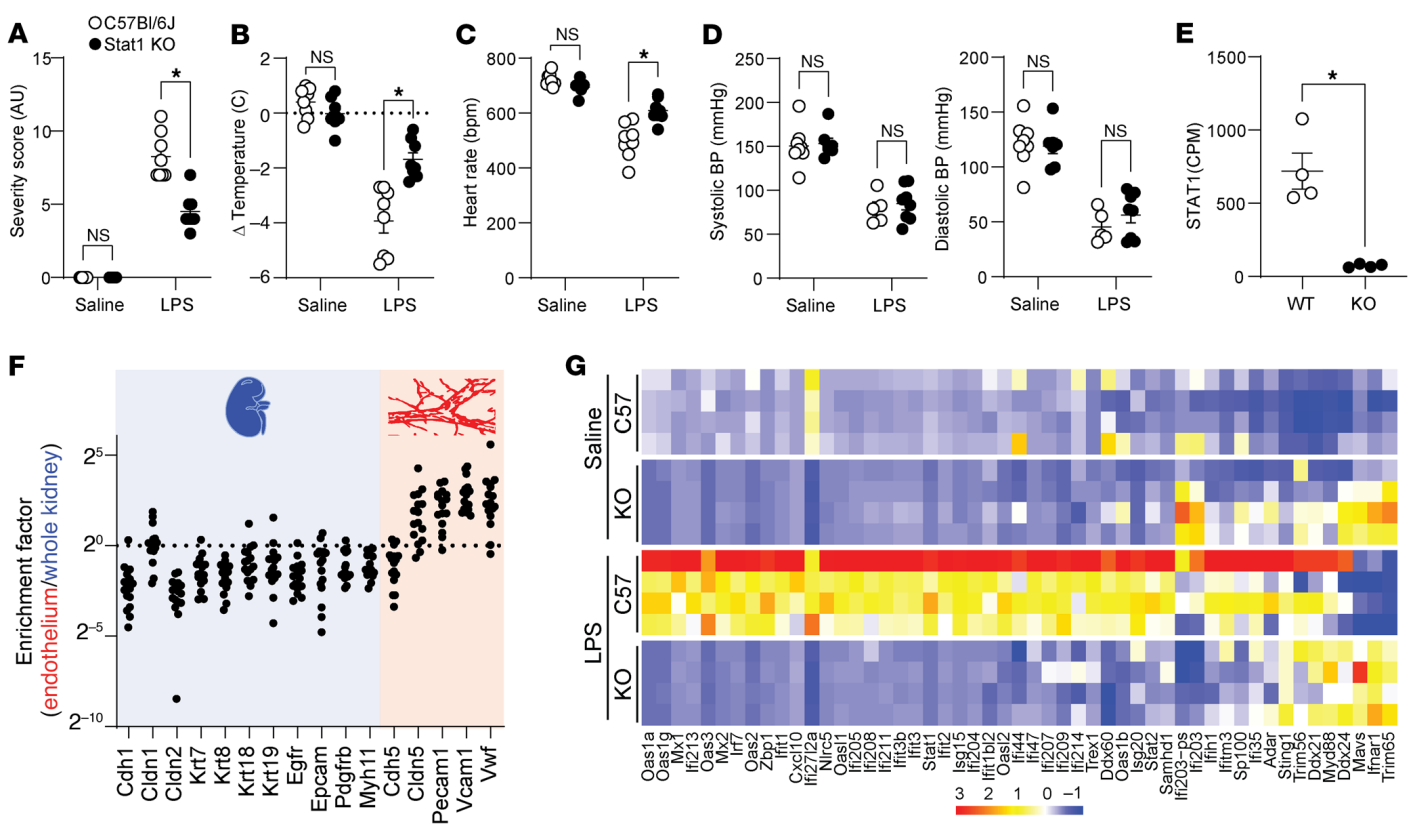
STAT1 is required for the kidney endothelial type I IFN–like response to LPS. STAT1^–/–^ or control C57BL/6J mice were challenged with an intraperitoneal bolus of LPS for 15 hours. Disease severity (**A**) and body temperature (**B**), heart rate (**C**), and blood pressure (**D**) were measured prior to euthanasia. Immediately afterward, kidneys were processed for RNA isolation from whole-tissue and from an endothelial cell–enriched fraction following a 2-step magnetic bead isolation procedure. (**E**) RNA-Seq counts demonstrating lack of STAT1 expression in STAT1^–/–^ mice (*n* = 4). (**F**) Ratio of EC-enriched over whole kidney RNA-Seq normalized counts obtained from the same organs. (**G**) Heatmap of kidney endothelium expression of ISGs. LUT corresponds to *z*-scores. **A**–**D**: Two-way ANOVA (*n* = 8). **E**: Welch’s *t* test. *P < 0.05.

We performed similar LPS challenges in mice lacking *Stat1* expression (*STAT1^–/–^* mice) and age-matched C57BL/6J controls. Liver RT-qPCR confirmed the lack of detectable *Stat1* expression in *STAT1*^–/–^ mice (Supplemental Figure 11). Similar to STING^iEKO^ mice, *STAT1^–/–^* mice showed overall reduced LPS-induced disease severity (Figure 13A) and hypothermia (Figure 13B). Consistent with severe LPS-induced shock, mice displayed hypotension and bradycardia (Figure 13, C and D). Notably, *STAT1^–/–^* mice displayed significantly reduced bradycardia compared with control mice (Figure 13C), despite reaching similar levels of hypotension (Figure 13D). Upon euthanasia, we obtained kidneys and performed endothelial cell enrichment for RNA-Seq as described above. Figure 13E shows confirmation of the loss of *Stat1* as a dramatic reduction in *Stat1* counts. Endothelial enrichment was confirmed as above (Figure 13F). Most importantly, the RNA-Seq analysis confirmed a crucial role for *Stat1* in endothelial ISG expression in response to LPS (Figure 13G). Expression of several ISGs was also lower in livers of *STAT1^–/–^* mice (Supplemental Figure 11).

## Discussion

Little is known about cell type–specific transcriptional profiles in failing organs. Here, we used mice expressing a tagged GFP/Rpl10a (TRAP) transgene specifically in the endothelium to identify expression of an IFN-like signature in the kidney and brain endothelial translatome in response to systemic shock induced by endotoxin that is limited by SOCS3. We further show that the kidney endothelium IFNI-like signature is promoted by *Sting1* and *Stat1* in response to LPS. The main advantages of TRAP approaches to single-cell RNA-Seq or endothelial cell enrichment protocols are (i) rapid processing of the tissue, enabling us to determine the specific translatome virtually at the moment of euthanasia via CHX perfusion and ribosome locking, (ii) high cell type specificity, and (iii) increased depth of RNA-Seq, allowing for the detection of low-count mRNAs. Using this approach, we identified a specific *Socs3*-dependent type I IFN–like response in the kidney and brain endothelium that correlated with circulating CXCL10 levels and with overall severity. Our mechanistic studies demonstrated that this was driven by early signals induced by IL-6 on vascular endothelial cells that triggered mtDNA release and a signaling cascade dependent on a cGAS/STING noncanonical pathway characterized by IRF1- and IRF3-dependent ISG expression in the absence of type I IFN signaling.

The role of SOCS3 in regulating this response suggested a mechanism involving JAK/STAT signaling upon an LPS challenge. We have recently demonstrated that an IL-6 autocrine signaling loop is required for complete endothelial barrier function loss upon LPS or TNF-α signaling (20). IL-6 signaling exerts pleiotropic effects in the endothelium via a complex signaling cascade that involves activation of several serine/threonine and tyrosine kinases (10, 11, 18), resulting (directly and indirectly) in activation of multiple transcription factors, including STATs, AP1, and IRFs (17, 19, 21). Notably, membrane-bound IL-6 receptor preferentially promotes STAT1 phosphorylation in T cells (42), and mouse fibroblasts lacking STAT3 respond to IL-6 with an IFN-γ–like transcriptional response (43). While the mechanisms leading to STAT3 activation are well understood, little is known about how IL-6 promotes the activation of many other transcription factors. Experiments shown here with human primary endothelial cells demonstrated that IL-6 *trans*-signaling directly promotes expression of multiple ISGs via activation of a JAK/STAT1 signaling pathway, rather than the canonical STAT3-dependent transcriptional control that we previously showed to regulate endothelial barrier function in these cells (17). Moreover, our data support a model in which STAT1 mediates ISG expression downstream of IL-6 signaling, in a process that is qualitatively distinct from the STAT3-dependent transcription. Our gene knockdown experiments demonstrate that STAT3 could not compensate for STAT1 loss, nor could STAT1 compensate for STAT3 loss.

This suggests that permeability and DNA-sensing responses are driven by parallel pathways downstream of this cytokine. Although the pathway was initially thought to exclusively mediate IFNI expression as part of the antiviral response, it is now increasingly recognized that chronic inflammatory diseases can promote activation of this pathway by mechanisms that do not involve viral DNA (29–31). Our results indicate that IL-6–induced mtDNA triggered a pathway in endothelial cells that diverged from IFNI antiviral responses, as we found no evidence of IL-6 inducing IFNI. In endothelial cells, rapid IFN expression was shown in response to several NF-κB–dependent proinflammatory stimuli (32–34). IL-6, however, does not induce NF-κB activity or downstream targets such as *ICAM1*, *TNF*, or *IL1B* (19). Consistently, we found no evidence of IFN induction by IL-6 (19). Moreover, we demonstrate that a canonical IFNI autocrine loop was not required for induction of ISG expression by IL-6, because (i) our experiments using CHX as a pretreatment demonstrated that protein synthesis was not required for induction of ISG expression and (ii) *IFNAR*-knockdown experiments showed that *IFNAR* expression was dispensable for IL-6–induced changes. Enzymatic cGAS activity and expression of *CGAS*, *STING*, *IRF1*, *IRF3*, and partially *IRF4* were required for ISG expression. While we did not find evidence of phosphorylated STAT2 and IRF9 involvement, further experiments are required to formally test any potential role for IRF9. Surprisingly, we found little evidence for IRF3 nuclear translocation, a finding that recalls the minimal STAT1 translocation. Because the siRNA-mediated knockdown experiments demonstrated that these proteins were required for IL-6+R–induced ISG expression, we hypothesize that only a small portion of IRF3 and STAT1 is sufficient to drive this gene expression. This is in contrast to the large increase in nuclear IRF1 observed. Moreover, overexpression of *IRF1* led to an increase in ISG expression and further enhanced IL-6–induced changes, demonstrating that this transcription factor is both necessary and sufficient for ISG expression. Experiments with LPS-treated endothelial cell–specific *Sting1*-knockout mice and global *Stat1*-knockout mice showed the crucial role for this noncanonical endothelial pathway in the development of shock.

There are several limitations to this study. First, we describe the response to endotoxemia, which does not fully recapitulate all aspects of sepsis; it will be important to confirm these findings in established septic models, such as cecal ligation and puncture or severe pneumonia. Second, the endothelial response was evaluated at a single time after challenge; a thorough analysis of the response kinetics may provide further insights into the mechanisms of endothelial transcriptional control. Third, while we demonstrate roles for STAT1, STING, and IRFs with multiple transgenic mice and human cultured endothelial cells, we cannot rule out a requirement for other stress sensors such as TLR3, MAVS, and others. Fourth, a causal role for this IFNI-like transcriptional response and specific downstream mediators in organ injury remains to be further tested.

Millions of critically ill patients are admitted to intensive care units (ICUs) every year (44–46). Severe systemic inflammatory reactions, including sepsis, often lead to shock, organ failure and death, in part through an acute release of cytokines that promote vascular dysfunction (1–5). However, simply blocking cytokine activity does not improve survival during sepsis (47). Cytokine blockade or steroid treatments dampen the initial inflammatory response but also prevent the immune system from fighting and clearing the primary infection (47), which could explain early failures in clinical trials (48–51). Thus, therapies for septic shock are limited to infectious source control and hemodynamic support (52). To save these patients, there is an urgent need to understand the mechanisms by which some patients recover from shock, while others experience disease progression that continues unabated until the damage is irreversible and results in death. Multiple endothelial proteins contribute to organ failure by causing systemic vascular leak, coagulopathy, leukocyte adhesion, and vasodilation (3–9). We posit that limiting endotheliopathy will lead to improved therapies to prevent organ damage and mortality without interfering with the required pathogen clearance: that is, targeting mechanisms downstream of cytokine signaling and regulating endothelial dysfunction, rather than the cytokines themselves. As an early-response cytokine, IL-6 is poised to induce a universal inflammatory response quickly after pathogen contact or tissue damage. As such, it may induce both antibacterial and antiviral responses to ensure a broad-spectrum response. This could have evolutionary advantages to quickly limit pathogen progression. However, it may also lead to increased collateral damage during severe systemic inflammatory processes. Controlling the intracellular response to IL-6 by promoting the required signaling arm and limiting unnecessary responses (e.g., antiviral genes in response to bacteremia) is a promising therapeutic avenue to reduce the risk of multiorgan dysfunction without impeding pathogen clearance.

## Methods

### Sex as a biological variable

Assignment of mice to treatment groups was performed through randomization of mice within each genotype for every litter and balanced for sex distribution. Similar findings are reported for both sexes. HUVECs were collected from umbilical cords without information of neonate donor’s sex.

### Materials

The commercial sources for critical reagents and their catalog numbers are listed in Supplemental Table 1. Supplemental Table 2 lists all antibodies used. Supplemental Table 3 provides a list of sequences for RT-qPCR primers.

### Mice

#### Genetics.

Endothelial cell–specific, tamoxifen-inducible *Socs3*-knockout mice were generated by breeding B6.Tg(Cdh5-cre/ERT2)1Rha (Cdh5-CreER^T2^ endothelial driver) mice (38) with B6;129S4-Socs3^tm1Ayos^/J (*Socs3*^fl/fl^ conditional knockout) (39) mice as described previously (19). Mice were backcrossed to a full C57BL/6J background by breeding to C57BL/6J mice (The Jackson Laboratory strain #000664) for at least 10 generations. TRAP mice were generated by removing a tdTomato transgene and breeding with B6.129S4-Gt(ROSA)26^Sortm1(CAG-EGFP/Rpl10a,-birA)Wtp^/J (Rosa26fsTRAP) mice (37) previously backcrossed to a C57BL/6J background (a gift from Patrick Murphy, University of Connecticut). Endothelial cell–specific TRAP WT and SOCS3^iEKO^ mice were recently described (21, 22). Knockout, heterozygous, and control littermates were obtained by crossing Cre^+^; Rosa26fsTRAP^+^; *Socs3*^fl/+^ mice. Genotypes and sex were confirmed by PCR genotyping (Supplemental Table 4). All mice received tamoxifen (2 mg tamoxifen in 100 μL via intra-peritoneal (IP) injection) at 6–9 weeks old for 5 consecutive days. Deletion of target gene after tamoxifen was confirmed by tail digestion and PCR. All the experiments were conducted between 2 and 3 weeks after the end of tamoxifen treatment. Endothelial cell–specific STING knockout mice were previously reported (34). Gene deletion was induced by intraperitoneal injection of 2 mg tamoxifen in oil (100 μL) at 6–9 weeks of age for 3 consecutive days. Control mice received a similar amount of vehicle (oil) but no tamoxifen. Experiments were conducted 2 weeks after the end of tamoxifen treatment. 7-week-old B6.129S(Cg)-Stat1^tm1Dlv^/J (*Stat1*^–/–^, The Jackson Laboratory strain #012606) and age-matched WT C57Bl/6J mice were obtained from the Jackson Laboratory. Experiments were performed after a 2-week acclimation period.

#### Housing.

Mice were housed in specific pathogen free rooms with 12-hour light/dark cycles and controlled temperature and humidity. Mice were kept in groups of 5 or less in Allentown cages with access to food and water ad libitum.

#### Endotoxemic model.

Severe, acute inflammation was induced by a single IP injection of a bolus of 250 μg/250 μL LPS. We previously reported that this dose led to 100% mortality in SOCS3^iEKO^ mice, but is nonlethal in littermate WT *Socs3* mice (19) and leads to substantial transcriptional changes in kidney and brain endothelium (21, 22). Control mice were given an IP injection of 250 μL of sterile saline solution. A severity scoring system was used to assess the response to LPS as previously published (19, 21, 22). Body weight (standard scale) and temperature (infrared thermometer at the base of the skull) were obtained prior to LPS or saline injection and immediately after scoring.

#### Experimental design.

Mice from 10 litters were used in 6 independent SOCS3^iEKO^ TRAP experiments. A mouse with a severity score=0 after LPS treatment was excluded from the studies. Two independent experiments from 4 litters were used for the STING^iEKO^ assays. Two independent experiments (*n* = 4 per group in each experiment) were performed on *Stat1*^–/–^ mice. Assignment to the saline or LPS groups were performed through randomization of mice within each genotype for every litter and balanced for sex distribution. All handling, measurements, and scoring were performed blindly to treatment and genotype groups and based on mouse ID# (ear tags or tail tattoos). Experimental groups were unmasked at the end of each experiment.

### Endothelial gene expression

#### Total and TRAP RNA isolation.

TRAP RNA isolation was performed following established protocols (37, 53) with small modifications. Mice were euthanized with an overdose of pentobarbital. Immediately upon loss of paw reflex, the chest cavity was opened, and mice were subject to intracardiac perfusion via the left ventricle with ice-cold HBSS containing 100 μg/mL CHX for 2 minutes at a rate of 5 mL/min. CHX perfusion is required to lock ribosomes in place for subsequent immunoprecipitation. Kidneys and brains were immediately removed, transferred to plates containing 1 mL of dissection buffer and minced with razor blades prerinsed with RNAse Away solution. Approximately 100 mg of minced kidneys were transferred to RNAse-free tubes and lysed with RNAse-free disposable pestles until visually homogeneous. Samples were then centrifuged at 4°C, 2,000 × *g* for 10 minutes and the supernatant was transferred to a new tube prior to adding 1/9 volume of 10% Igepal and 1/9 volume of 300 mM DHPC. Tubes were mixed by inversion several times and incubated for 5 minutes on ice. Following incubation, samples were centrifuged at 4°C, 20,000 × *g* for 10 minutes. 50 μL of the supernatant was separated for processing for total RNA and the rest was transferred to a new tube for ribosome pull-down. Total RNA was obtained by adding 500 μL of Trizol to each tube and performing extraction with 100 μL of chloroform. The RNA was obtained from the aqueous fraction by adding an equal volume of 70% EtOH (approx. 350 μL) and using RNeasy Micro spin columns (Qiagen) following manufacturer’s instructions. The remainder of the supernatant/Igepal/DHPC solution was processed for TRAP. Briefly, each tube was incubated with prewashed Dynabeads on a nutating rocker for 15 minutes at RT to remove nonspecific binding to the beads and separated with a magnet. The supernatant was then incubated with Dynabeads bound to anti EGFP antibodies on a nutating rocker for 45 minutes at 4°C. After 3x washes with RNA buffer, the beads were allowed to reach RT and then incubated for an additional 5 minutes in RLT buffer containing β-mercaptoethanol. RNA was then purified using RNeasy Micro spin columns following manufacturer’s instructions.

#### Enrichment of endothelial cells.

Kidney endothelial cells were enriched as described previously (21). Briefly, after euthanasia, kidneys from STING^iEKO^, *Stat1^–/–^*, or control mice were minced into small pieces (~1 mm^3^) and digested with 5 mL of PBS containing calcium and magnesium plus 100 mg of type 1 collagenase and 100 mg dispase II for 10 minutes at 37°C. The slurry of tissue and buffer was triturated by passage through a 14-gauge cannula attached to a 10 mL syringe approximately 10 times, followed by a 20-gauge cannula attached to a 10 mL syringe (10 times), and finally filtered through a 70 μm cell strainer. After a wash with 5 mL of PBS + 0.1% BSA, single cell suspensions were centrifuged at 400 × *g* for 8 minutes at 4°C and pellets were resuspended with 1.5 mL of ice-cold PBS + 0.1% BSA. One aliquot (75 μL) per sample was lysed in Trizol to obtain whole kidney RNA. The remainder cell suspension was subjected to negative selection by incubating cells with 100 μL of streptavidin-conjugated Dynabeads bound to biotinylated EpCAM and biotinylated CD45 antibodies for 10 minutes at RT with rotation. Following this, tubes were placed on a Dynal MPC-S magnet for 5 minutes. Remaining cells in supernatants were subjected to positive selection by incubating cells with 100 μL of streptavidin-conjugated Dynabeads bound to biotinylated CD31 antibodies for 10 minutes at RT with rotation. Cells were collected with a magnet and the supernatant was discarded. Any remaining contaminating cells were removed by washing cells 6 times with PBS containing 0.1% BSA. RNA isolation of these cells was done following the RNeasy Plus Mini Kit (Qiagen) protocol.

#### RNA sequencing and bioinformatics.

SOCS3^iEKO^ and control brain TRAP-Seq and bulk RNA-Seq was published recently (22) and deposited in NCBI’s Gene Expression Omnibus (GEO Series accession number GSE253438). 500 ng of total and TRAP RNA, or 50–200 ng of endothelial cell–enriched RNA from each kidney were processed for next-gen sequencing by Azenta Life Sciences. RNA-Seq libraries were prepared using Illumina’s TruSeq protocol and were sequenced on an Illumina NextSeq 500. Reads were aligned to the mm10 genome using Rsubread v1.5.3 (54). Data were deposited in NCBI’s Gene Expression Omnibus and are accessible through GEO Series accession numbers GSE277696 (SOCS3^iEKO^), GSE277816 (STING^iEKO^), and GSE300619 (*Stat1^–/–^*). Gene counts were quantified by Entrez Gene IDs using featureCounts and Rsubread’s built-in annotation (55). Gene symbols were provided by NCBI gene annotation. Genes with count-per-million above 0.5 in at least 3 samples were kept in the analysis. Differential expression analysis was performed using edgeR (56) and limma-voom (57). GSEA (54, 58) was performed using GSEA v4.3.2 for Windows. Upstream analysis (enrichment in pathways and transcription factors) of each datasets was performed using the DecoupleR (59) package, the PROGENy model (58, 60), and the CollecTRI network. The full R code is provided as a Supplemental method.

### Immunohistochemistry

Kidneys were fixed for 48 hours in 10% formalin and then stored in 70% EtOH prior to dehydration and embedding in paraffin. 5 μm thick sections were deparaffinized and rehydrated in sequential baths with xylene and ethanol. Endogenous peroxidase activity was blocked by incubation for 10 minutes at RT in 0.5% H2O2/MeOH. Antigen retrieval was performed in an EZ Retriever (BioGenex) for 15 minutes at 98°C in buffer. After washes with PBS, slides were blocked with 5% fetal bovine serum and then incubated with an anti CXCL10 antibody in PBS (1:20) overnight at 4°C. Slides were then washed in PBS and incubated with biotinylated anti rabbit secondary antibody 1:500 in PBS+5% fetal bovine serum for 1 hour at RT. Biotin was detected using Vector Lab’s ABC kit Elite and ImmPACT DAB as per manufacturer’s instructions. Slides were then counterstained with hematoxylin, dehydrated, and mounted with VectaMount. Stain controls lacking primary antibody did not show any detectable signal and are shown in Supplemental Figure 12.

### Cell culture and treatment

#### Cells.

The brain endothelial cell line HCMEC/D3 was obtained from Millipore Sigma (catalog number SCC066) and cultured in EndoGRO-MV Complete Culture Media (Millipore Sigma). All experiments were performed within 10 passages. Human primary renal glomerular endothelial cells (HRGEC) were obtained from Cell Biologics (catalog number H-601G) and cultured in complete endothelial cell media with supplement kit (Cell Biologics). HUVECs were isolated in-house according to established protocols as previously described. Briefly, umbilical cords (20–30 cm) were obtained from Cesarean sections scheduled at Albany Medical Center. Following cord blood retrieval, cords were stored at 4°C in phosphate-buffered saline (PBS) containing a mixture of penicillin and streptomycin. Cords were used within 24 hours or discarded. Samples were anonymized following the recommendations of the Albany Medical Center IRB. Cords were quickly rinsed in 70% ethanol and sterile PBS. Then, vein lumens were washed with PBS to remove remaining blood and clots and then incubated for 30 minutes at RT in sterile PBS containing 0.2% collagenase, pH 7.4 with gentle massaging. Released cells were collected in growth media containing phenol red-free EBM2 media supplemented with EGM-2 Growth Medium 2 Supplement Mix, penicillin, streptomycin, and amphotericin B. Cells were then centrifuged, resuspended in fresh growth media and plated in plastic culture flasks precoated with 0.1% gelatin. Upon reaching initial sub-confluence (4–7 days), cells were amplified by serial passage twice and frozen in liquid nitrogen until use. Identity and purity of the HUVEC isolations was confirmed once for each isolation by > 99% positive immunostaining with endothelial cell markers (FITC-*Ulex europaeus* lectin, VE-cadherin) and > 99.9% negative for α-smooth muscle actin. Thawed vials were passaged 3 times per week in the absence of antibiotics and assayed between passages 4 and 8. Unless otherwise stated, for all assays, cells were plated at full confluency at a density of 8 × 10^4^ cells/cm^2^ on plates precoated for 30 minutes with 0.1% gelatin and incubated at least 48 hours prior to the start of experiments.

#### Treatments.

Cells were treated with 30 μg/mL 2’3’ cGAMP (stock: 1 mg/mL dissolved in water), or inhibitors to cGAS activity (10 μM RU.521, stock: 5 mM dissolved in DMSO), or protein synthesis (10 μg/mL CHX, stock: 5 mg/mL dissolved in PBS). A similar amount of water, PBS, or DMSO was added to vehicle control wells. To induce IL-6 signaling, cells were treated with a combination of 200 ng/mL recombinant human IL-6 and 100 ng/mL sIL-6Rα (IL-6+R). In some experiments, cells were treated with 2,000 U/mL IFN-α_2_ or 500 U/mL IFN-β.

### Gene knockdown

Small interference RNA (siRNA) against *STAT1*, *STAT3*, *CGAS*, *STING*, *IFNAR1*, *IRF1*, *IRF2*, *IRF3*, and *IRF4* were obtained as a pool of 4 sequences from Horizon Discovery (On target plus SiRNA). Cells were transfected with siRNA complexed with Lipofectamine siRNA iMAX (Invitrogen) in suspension and then seeded at slightly above confluence (1 × 10^5^ cells/cm^2^) to account for transfection toxicity. Controls were transfected with nontargeting control pooled duplexes (Horizon Discovery). Cells were incubated for 2–3 days prior to treatments or lysis. Knockdown efficiency was determined by Western blotting and qPCR analysis.

### Lentiviral delivery

#### Production of viral particles.

Lentiviral constructs to express *SOCS3* with C-terminal Myc and DDK tags were described previously (19). Lentiviral constructs to express *IRF1* with C-terminal Myc and DDK tags were generated by cloning *IRF1* from a Human Tagged Lenti ORF Clone (Origene catalog number RC203500L1) into a pLenti-C-Myc-DDK-P2A-tGFP (Origene PS100088) lentiviral gene expression vector. Lentiviral particles were grown in HEK293FT cells by cotransfecting cells with the *IRF1*-expressing lentiviral construct or pLenti-C-Myc-DDK-P2A-tGFP (empty vector control) and pCMV-dR8.2 dvpr and pCMV-VSVG packaging plasmids (61). After 48 hours, media was concentrated using a 30 kDa cutoff filter, aliquoted, and stored at –80°C until use. Viral titer was determined by the proportion of infected cells with green fluorescence 72 hours after infection.

#### Transductions.

Cells were seeded at 50% confluence and allowed to attach and spread overnight. Then, cells were incubated with lentiviral particles overnight. Dilutions of each viral prep were obtained to achieve similar levels of GFP mRNA expression as determined by RT-qPCR (19). Transduced cells were then lifted and seeded for the required experiments.

### RNA isolation and RT-qPCR

Confluent monolayers grown in multiwell plates were lysed with Trizol reagent and total RNA was isolated following manufacturer’s instructions. 400 ng of total RNA were used to prepare cDNA using Primescript RT Master Mix at 42°C following manufacturer’s instructions. cDNA was diluted 10-fold in nuclease-free water and then 2 μL of cDNA were used per PCR reaction. qPCR was performed in a StepOnePlus (Applied Biosystems) instrument using SYBR green-based iTaq supermix and 2 pmol primers (Thermo Fisher). Fold induction was calculated using the ΔΔCt method using *GAPDH* (human RNA) or *Hprt1* (mouse RNA) as housekeeping genes.

### Western blotting

Confluent HUVEC were lysed in Laemmli buffer containing cOmplete protease inhibitor mixture, PhosSTOP phosphatase inhibitor mixture, 0.1 M NaF, 0.1 mM phenyl arsine oxide, 10 mM pyrophosphate and 0.1 mM pervanadate. After boiling for 5 minutes, a total of 15 μL of cell lysate per lane was loaded on standard SDS-PAGE gels and transferred to nitrocellulose membranes. Immunoblots were performed by blocking the membranes with 5% BSA in PBS/Tween and incubating overnight at 4°C with respective primary antibodies as described in Supplemental Table 2. Secondary HRP-conjugated anti-mouse or anti-rabbit antibodies were incubated for 1 hour at room temperature. Membranes were developed via chemiluminescence detected with a Chemidoc MP (Bio-Rad) imaging system. Band quantification from raw image files was performed using Bio-Rad’s ImageLab 6.01.

### mtDNA

Confluent cells grown in 6-well plates were lysed in 1% Igepal CA-630 in mQ water. After scraping, lysates were incubated on ice for 15 minutes and then centrifuged at 16,000 × *g* for 15 minutes at 4°C. DNA purification from supernatants (soluble cytosolic fraction) was done using DNeasy Blood & Tissue Kits following manufacturer’s instructions. 90 ng of total DNA were used for qPCR reactions against *MT-CO1*.

### Subcellular fractionation

Confluent cells grown in 6-well plates were lifted with trypsin, centrifuged, and resuspended in 1 mL PBS. An aliquot was separated for whole cell lysate and immediately mixed with 2x Laemmli buffer. The remainder was processed for cytoplasmic and nuclear enrichment using a NePer kit following manufacturer’s instructions. Similar lysate volumes were loaded in SDS-PAGE gels for Western blotting. Tubulin and Lamin A/C were used for subcellular enrichment controls.

### Immunofluorescence

Confluent cells were grown on 8-well μ-slide chambers (Ibidi) precoated with 0.1% gelatin. At 48 hours after seeding, cells were treated as indicated. Cells were then fixed with 4% paraformaldehyde in PBS for 30 minutes at 4°C and then washed twice with PBS. Fixed cells were permeabilized with 0.1% Triton X-100 in PBS (PBS-TX) for 15 minutes and blocked with 5% bovine serum in PBS-TX for 1 hour. Antibodies were incubated for 2 hours at room temperature. Slides were then washed in PBS-TX and stained with Alexa Fluor–conjugated secondary antibodies and 0.5 μg/mL DAPI for 1 hour at RT. Slides were then washed in PBS. Images were taken at magnification 20x and 63x using a Zeiss AXIO Observer Z1 microscope or at 63x using a Zeiss LSM880 with Airyscan microscope. Images were processed for linear contrast and pseudocoloring using Zeiss Zen Blue software. Stain controls lacking primary antibody did not show any detectable signal and are shown in Supplemental Figure 12.

### TFAM aggregation

Cells were stained via immunofluorescence against TFAM and Tomm20. Z-stack images were obtained with a Zeiss LSM880 with Airyscan at 63X. Upon 3D Airyscan processing using default settings in Zen Blue, images were imported into Imaris V10.1. Individual channels were processed for object rendering. Individual object sizes per imaging field were tabulated and exported to GraphPad Prism for the calculation of averages and cumulative distribution plotting.

### Statistics

Groups were analyzed with a combination of parametric or nonparametric 1- or 2-factor tests, as described in each of the figure legends. All statistical tests were performed with GraphPad Prism version 6 or 10. In all figures, asterisks denote *P* < 0.05. Homogeneity of variance of 2 groups was tested via an *F* test, or a Bartlett’s test for more than 2 groups. Unpaired *t* tests or 1- or 2-way ANOVAs were performed if variances were not statistically different, or Welch’s *t* tests and ANOVA were performed in those cases with different variances. Some normalized values were analyzed by one sample *t* test as described in the respective legends. Normality was tested using the Kolmogorov-Smirnov test, and Mann-Whitney *U* tests were performed on non-normally distributed data. Severity scores (integers 0–15) were assumed not normally distributed. Bias in data interpretation was limited by masking of genotype and treatment groups and independent reproduction of all experiments shown herein.

### Data availability

Transcriptomics data were deposited in the NCBI’s Gene Expression Omnibus: GSE253438 (SOCS3^iEKO^ brain), GSE277696 (SOCS3^iEKO^ kidney), GSE277816 (STING^iEKO^ kidney), and GSE300619 (*Stat1^–/–^*). All other data are available in the main text or the supplemental material. Values for all data points in graphs are reported in the Supporting Data Values file. The workflow for RNA-seq analysis was done in R. The full code is provided as “Supplemental code” at the end of the supplemental materials PDF.

### Study approval

Mice were housed at the Animal Research Facility (ARF) at Albany Medical Center, which is accredited by AAALAC and licensed by the USDA and New York State Department of Public Health, Division of Laboratories and Research; or at Tufts University School of Medicine. All animal work was performed according to IACUC-approved protocols at Albany Medical College or Tufts University School of Medicine.

## Author contributions

Conceptualization: NM, PAV, PA, and APA. Methodology: NM, EKS, RBR, IDJP, FA, SL, DC, NP, MXGZ, UB, and APA. Investigation: NM, EKS, RBR, FA, PAV, PA, and APA. Visualization: NM, RBR, and APA. Funding acquisition: APA and PA. Project administration: APA. Supervision: APA and PA. Writing — original draft: NM and APA. Writing — review and editing: NM, EKS, RBR, SL, PAV, PA, and APA.

## Funding support

This work is the result of NIH funding, in whole or in part, and is subject to the NIH Public Access Policy. Through acceptance of this federal funding, the NIH has been given a right to make the work publicly available in PubMed Central

- NIH R01GM124133 and R35GM152454 (to APA), R01HL144477 and R01HL165725 (PA).
- Alzheimer’s Association grant ALZ-D-NTF 968957 (to APA).
- American Heart Association fellowships 22PRE916096 (to SL) and 24PRE1187744 (to EKS).

## Supplementary Material

Supplemental data

Unedited blot and gel images

Supporting data values

## Version 1 09/16/2025

In-Press Preview

## Version 2 11/03/2025

Electronic publication
