# Supplementary material for: Endothelial STING and STAT1 mediate IFN-independent effects of IL-6 in an endotoxemia-induced model of shock
Source: J Clin Invest. 2025 Sep 16;135(21):e189570. doi: 10.1172/JCI189570 (PMC12578408; doi:10.1172/JCI189570)
Supplement: Supplemental data [file jci-135-189570-s083.pdf]

Supplemental Figure 1

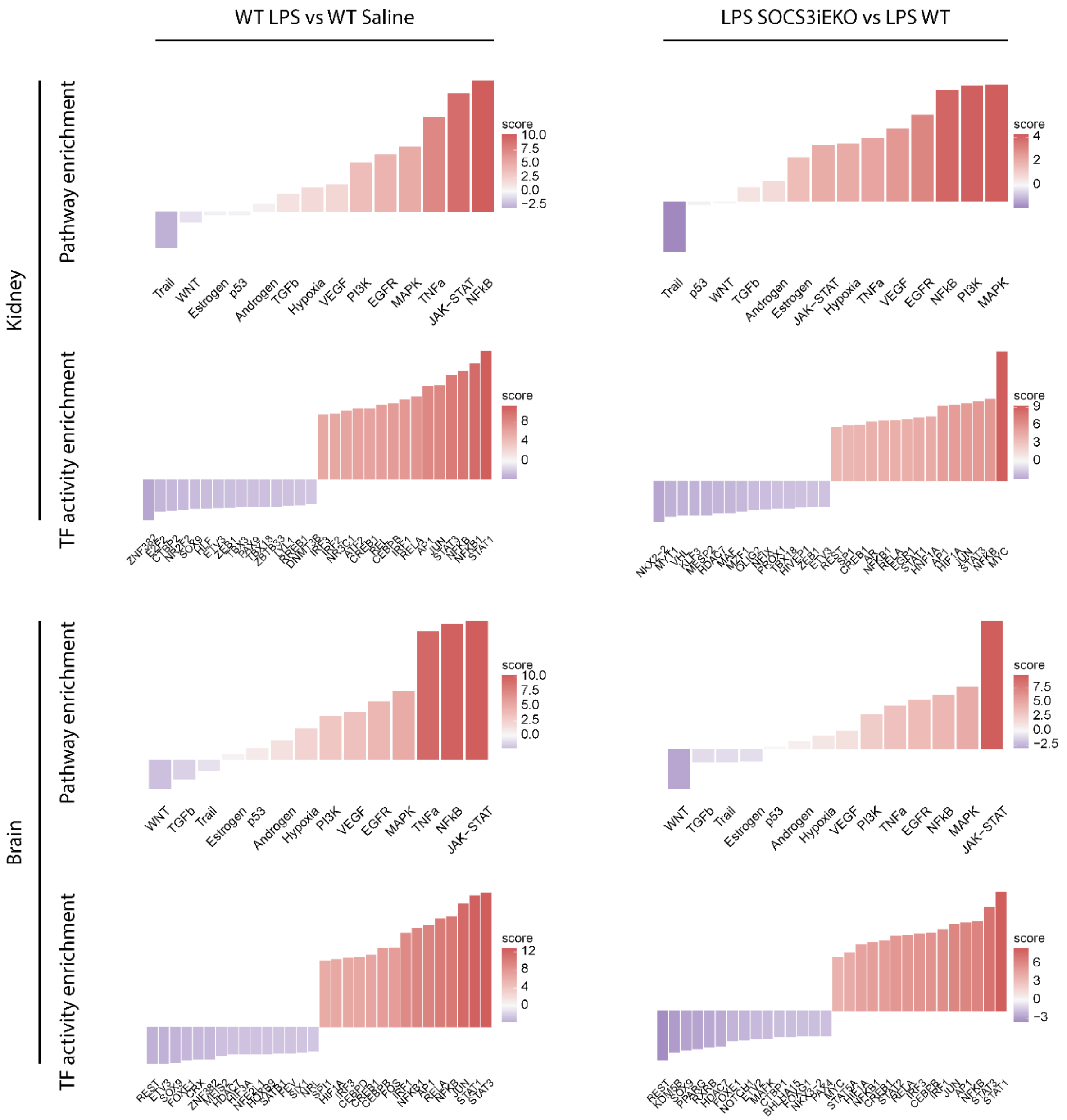

Supplemental Figure 1. Pathway and transcription factor activity enrichment in the endothelium of LPS-treated mice. The datasets shown in Figures 1 and 2 in the main text were analyzed for putative upstream signaling pathway and transcription factor activity following a workflow in R/Bioconductor consisting of the DecoupleR package, the PROGENy model, and the CollecTRI network as described in the supplemental methods.

Supplemental Figure 2

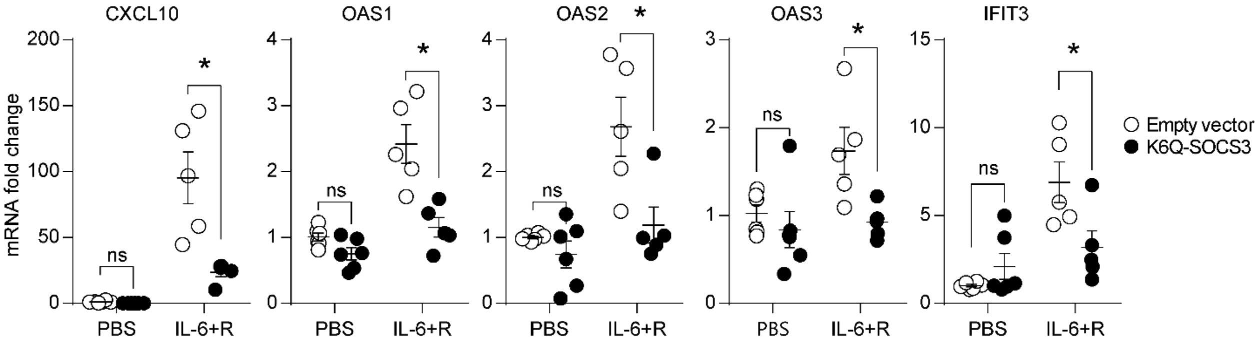

Supplemental Figure 2. SOCS3 overexpression prevents IL-6+R-induced ISG expression. Cells were transduced with an empty vector, or a vector coding for a stabilized form of SOCS3, as we described previously(*18*). Cells were treated for 2 hours with or without IL-6+R prior to lysis and RNA extraction for RT-qPCR. Data combined from three independent experiments performed in duplicate each. * $p<0.05$, Two-way ANOVA and Sidak post-hoc test.

Supplemental Figure 3

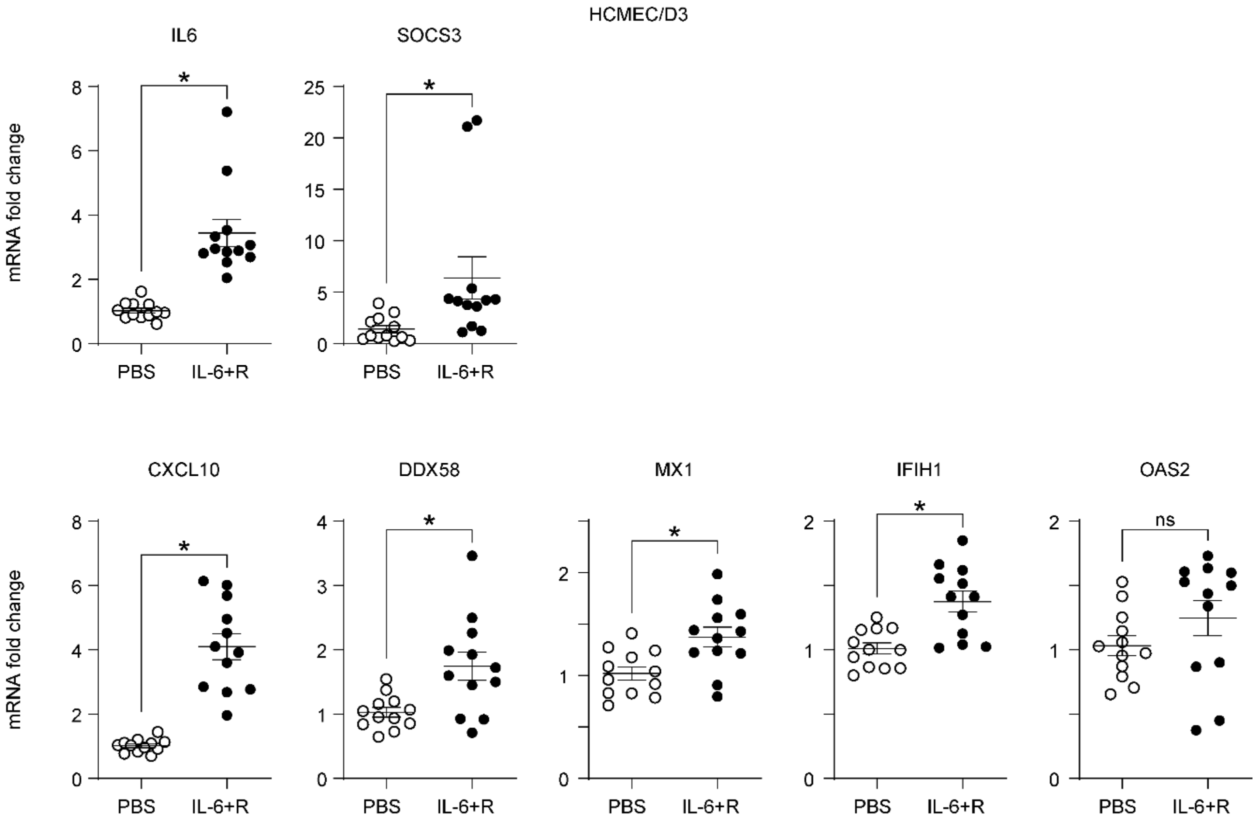

Supplemental Figure 3. IL-6+R-induced ISG expression in HCMEC/D3 cells. Cells were treated for 2 hours with or without IL-6+R prior to lysis and RNA extraction for RT-qPCR. Data combined from three independent experiments performed in duplicate each. * $p<0.05$, Two-way ANOVA and Sidak post-hoc test.

Supplemental Figure 4

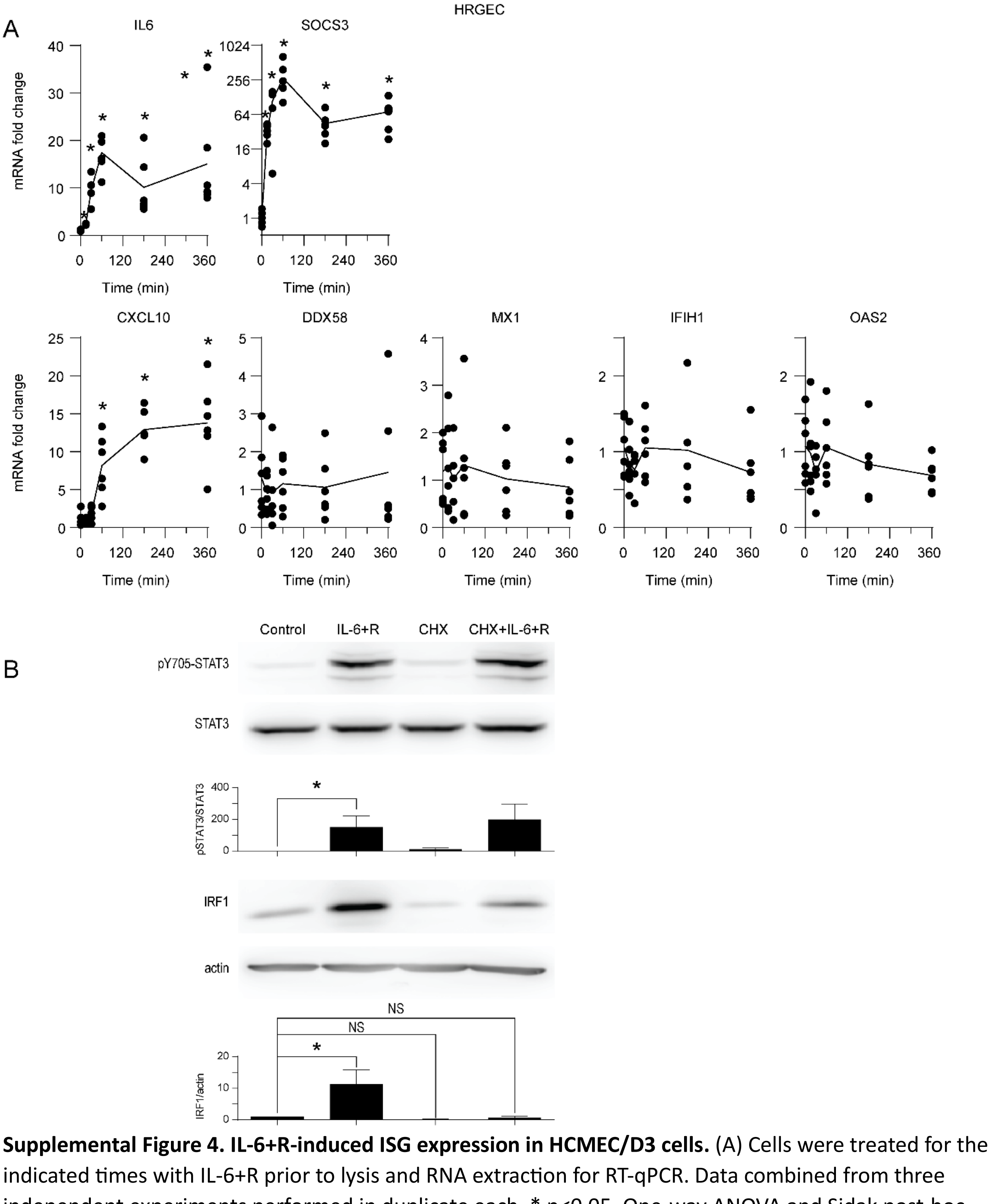

Supplemental Figure 4. IL-6+R-induced ISG expression in HCMEC/D3 cells. (A) Cells were treated for the indicated times with IL-6+R prior to lysis and RNA extraction for RT-qPCR. Data combined from three independent experiments performed in duplicate each. * $p<0.05$, One-way ANOVA and Sidak post-hoc test. (B) Cells were treated with or without IL-6+R for 2 h in the presence or absence of a 30 min

pretreatment with cycloheximide (CHX) prior to lysis and Western blotting. Two-way ANOVA and Sidak post-hoc test. Data from three independent experiments.

Supplemental Figure 5

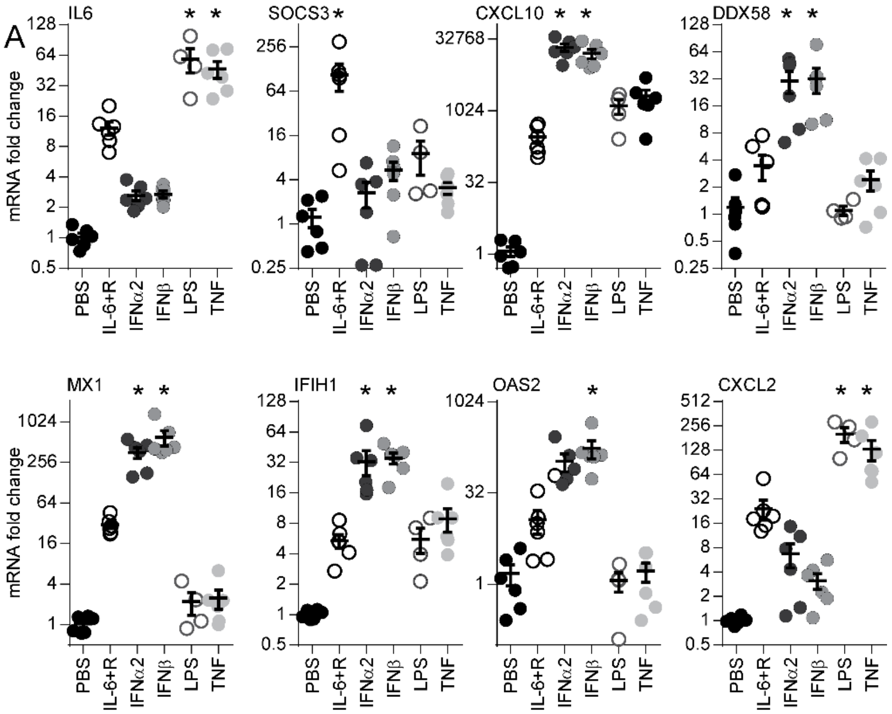

Supplemental Figure 5. The magnitude of ISG expression induced by IL-6+R is lower than IFNI-induced changes. HUVEC were treated for two hours with either IL-6+R, 2000 U/ml IFNα2, 500 U/ml IFNβ, 1 μg/ml LPS, or 20 ng/ml TNF prior to lysis and RT-qPCR. * $p<0.05$, one-way ANOVA and Sidak post-hoc test vs PBS.

Supplemental Figure 6

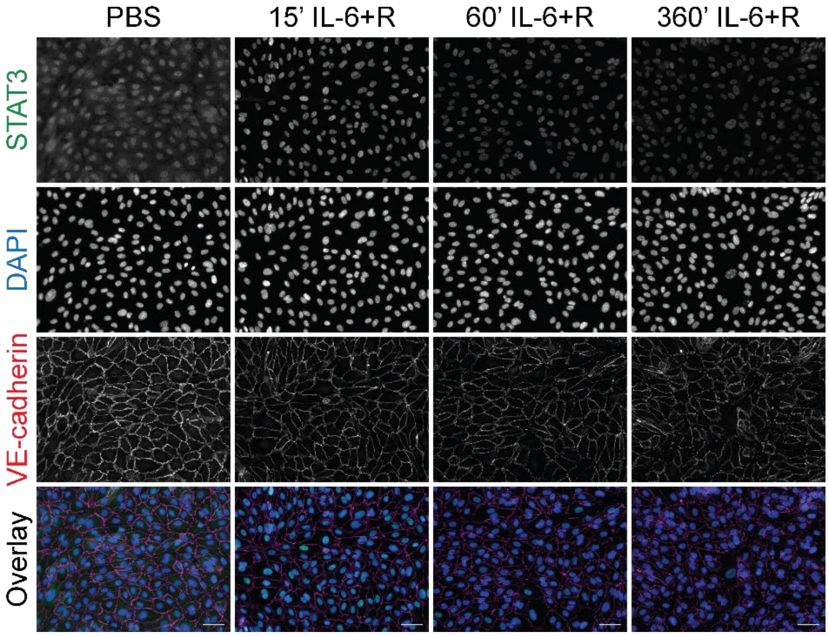

Supplemental Figure 6. IL-6+R induces rapid nuclear localization of STAT3. Immunofluorescence to detect total STAT3 in cells treated or not with IL-6+R. Cells were counterstained with a VE-cadherin antibody and DAPI to mark nuclei. Data representative of three independent experiments.

Supplemental Figure 7

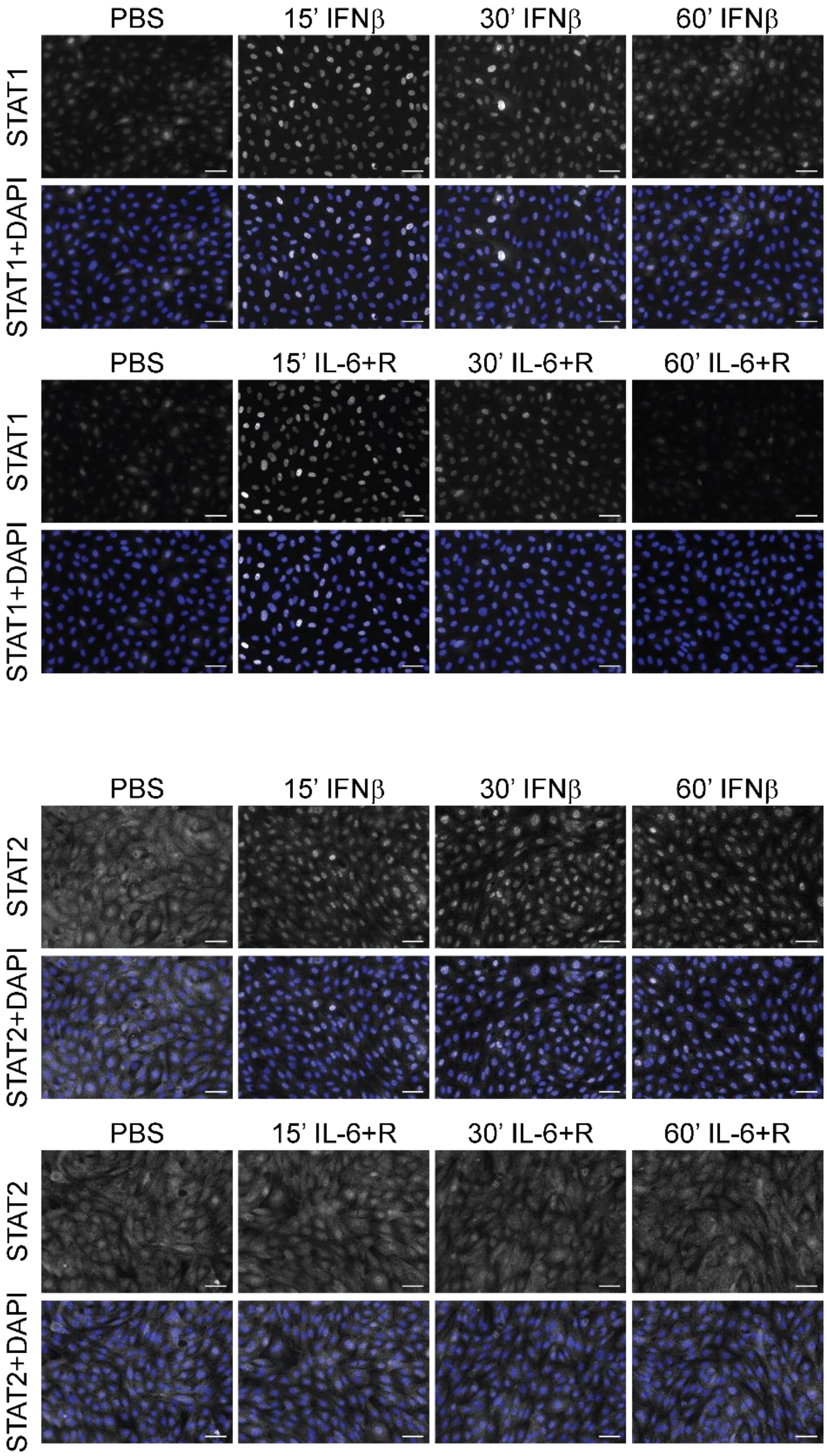

Supplemental Figure 7. IL-6+R induces rapid nuclear localization of STAT1, but not STAT2.
Immunofluorescence to detect total STAT1 and STAT2 in cells treated or not with IL-6+R. Cells were

counterstained with a VE-cadherin antibody and DAPI to mark nuclei. Data representative of three independent experiments. Cells were treated in parallel with 500 U/ml IFNβ as positive control for nuclear localization of STAT1 and STAT2.

Supplemental Figure 8

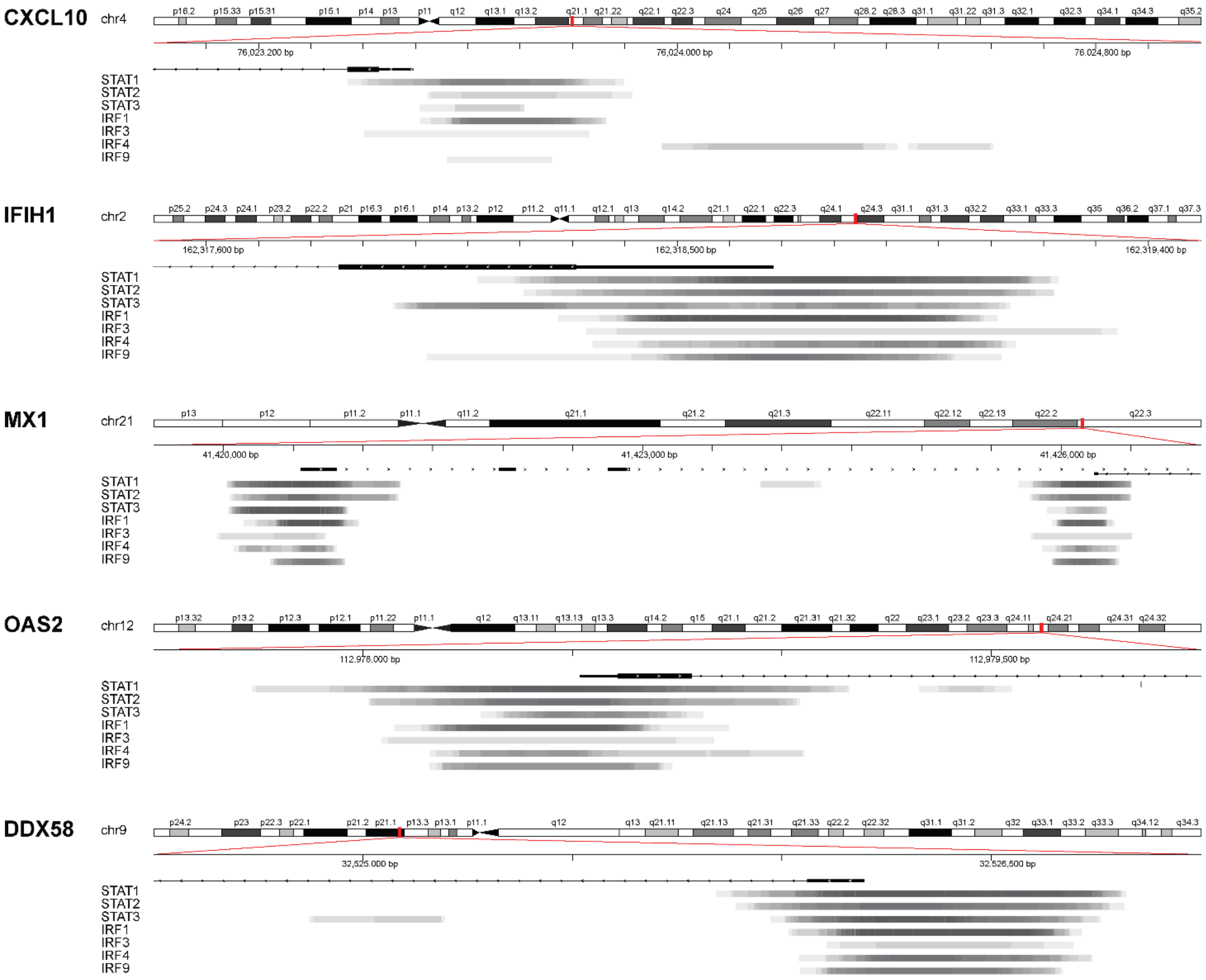

Supplemental Figure 8. Direct IRF binding to ISG promoters. The ChIP-Atlas database was interrogated on July 2023 for all known binding sites for STAT1/2/3 and IRF1/3/4/9, with no limits to cell type or experimental condition. Shown are the identified interactions within the proximal promoters of CXCL10, IFIH1, MX1, OAS2, and DDX58.

Supplemental Figure 9

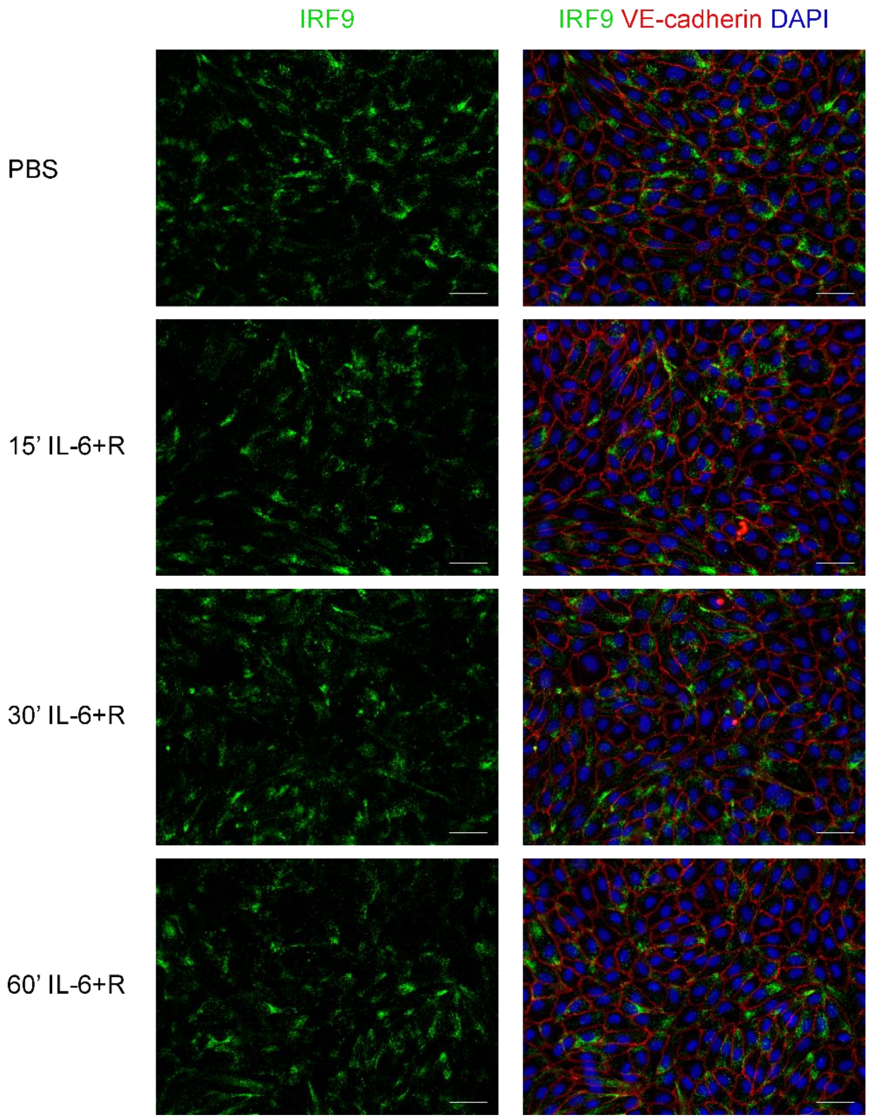

Supplemental Figure 9. IL-6+R does not induce nuclear localization of IRF9. Immunofluorescence to detect IRF9 in cells treated or not with IL-6+R. Cells were counterstained with a VE-cadherin antibody and DAPI to mark nuclei. Data representative of three independent experiments.

Supplemental Figure 10

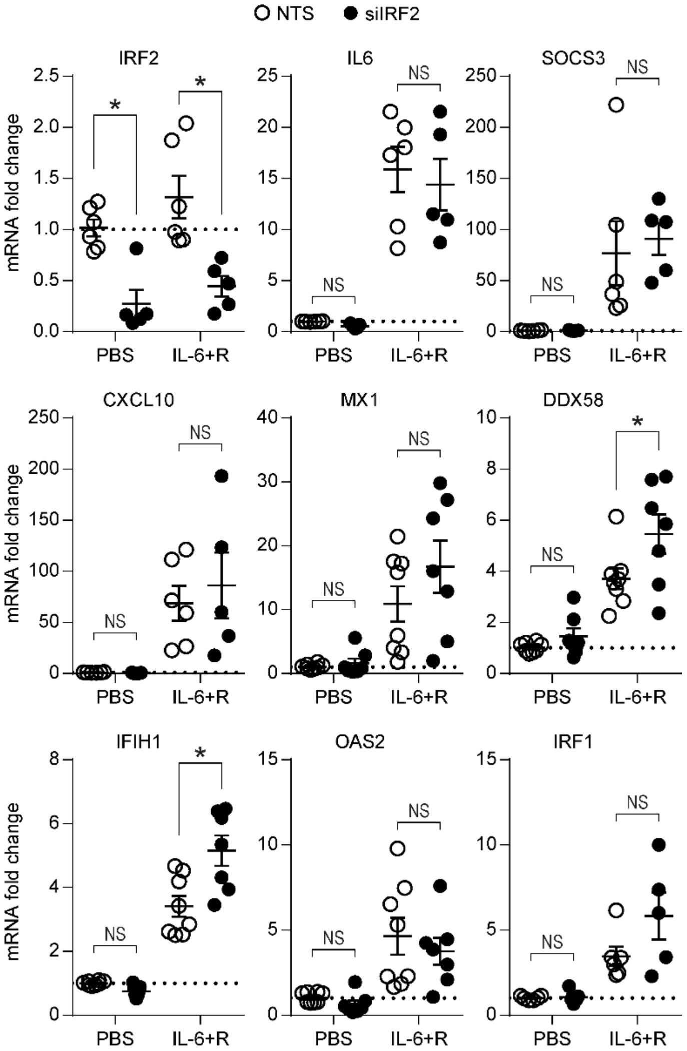

Supplemental Figure 10. ISG expression in HUVEC in response to IL-6+R does not require expression of IRF2. RT-qPCR of HUVEC transfected with NTS or IRF2 siRNA prior to treatment for 2 hours with or without IL-6+R. Two-way ANOVA and Sidak post-hoc test. * $p < 0.05$. Data representative of at least three independent experiments.

Supplemental Figure 11

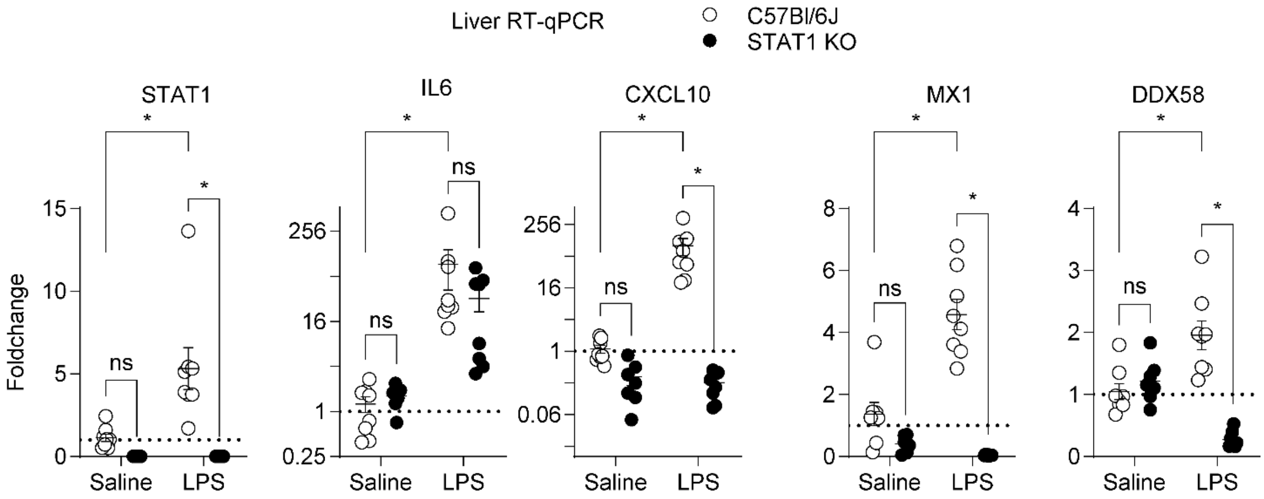

Supplemental Figure 11. LPS-induced liver ISG expression is reduced in endotoxemic STAT1-/- mice. RTqPCR of liver mRNA of STAT1-/- or control (C57Bl/6J) mice challenged with LPS as described for figure 12 in the main text. * $p<0.05$. Two-way ANOVA and Sidak post-hoc test (n=8).

Supplemental Figure 12

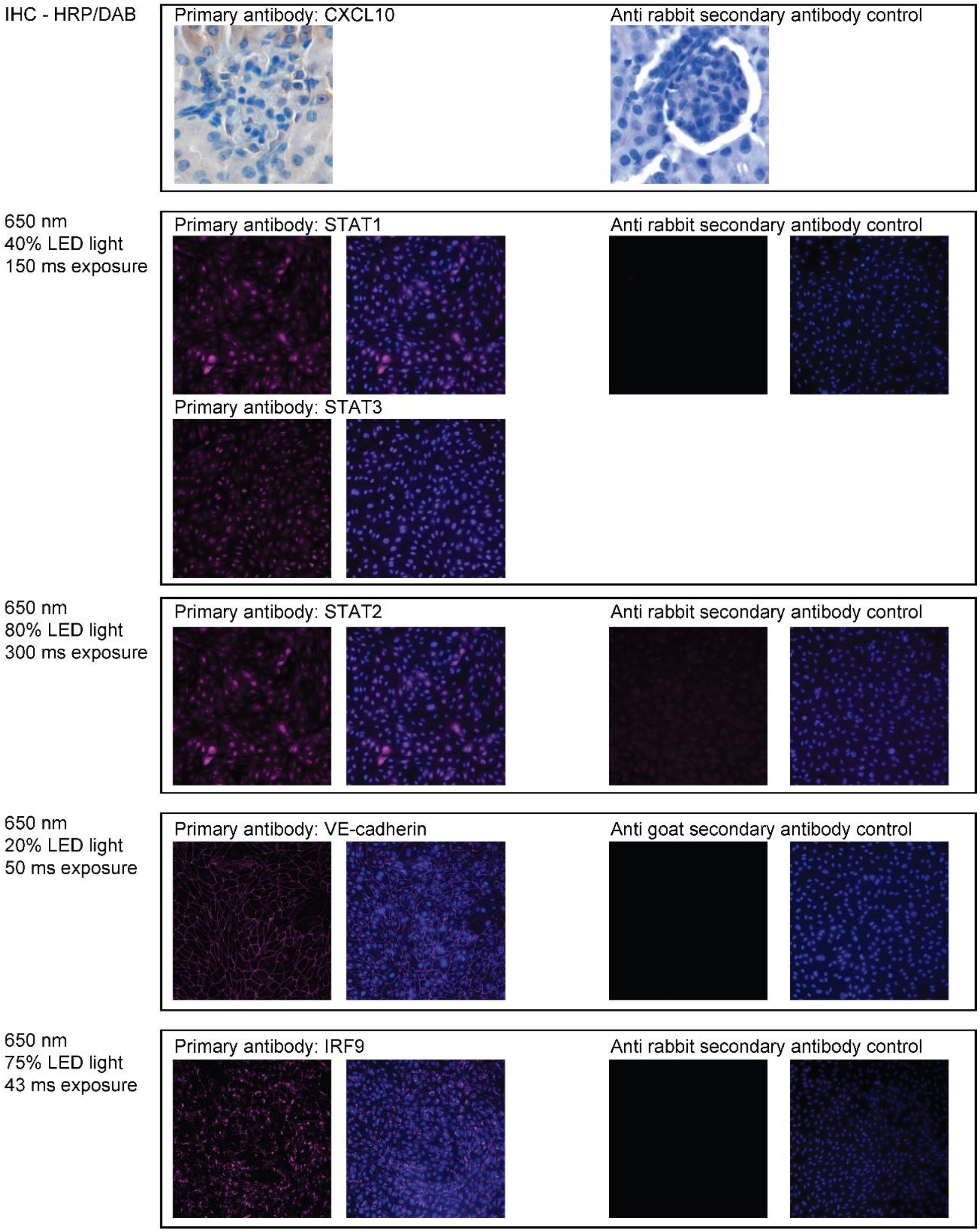

Supplemental Figure 12. Secondary only staining controls. Kidneys sections (IHC) or HUVEC (IF) were stained for the respective proteins and counterstained with hematoxylin or DAPI. In parallel, other sections or cultured cells were processed exactly as for the corresponding staining, but without incubation with the primary antibody. Control images were taken with the same conditions as their corresponding target.

Supplemental Table 1 - Commercial sources for critical reagents and their catalog numbers

M&M section	Reagent or disposable	Vendor	Catalog Number	Concentration/ Dose
Mice	Tamoxifen	Sigma Aldrich	T5648	2 mg in 100 µL
	Peanut Oil	Sigma Aldrich	P2144	N/A
	Lipopolysaccharides	Sigma Aldrich	L4391	250 µg/ 250 µL
Endothelial Enrichment	Collagenase Type 1	Worthington Biochemicals	LS004196	0.20%
	RNAse A	Qiagen	19101	100 mg/ml
	Dispase II	Roche	10295825001	0.20%
	DNeasy Blood and Tissue kit	Qiagen	69504	As per instructions
	RNEasy Plus Micro Kit	Qiagen	74034	As per instructions
	BD Falcon cell strainer 70um pore size	Thermo Fisher	352350	N/A
	Dynabeads M-280 Streptavidin	Thermo Scientific	11205D	1mg/100 µL
Immuno-histochemistry	ABC kit Elite	Vector Labs	PK-6100	N/A
	ImmPACT DAB	Vector Labs	SK-4105	N/A
	VectaMount	Vector Labs	H-5000	N/A
	Fetal bovine serum	Sigma Aldrich	F4135	5%
Cell Culture	Recombinant Human IL6 protein	R&D Systems	206-IL-200/CF	200 ng/mL
	Recombinant human IL6r alpha	R&D Systems	227-SR-025/CF	100 ng/mL
	Phenol red-free EBM 2 media	PromoCell	C-22211	N/A
	Growth medium 2 supplement mix	Promocell	C-39216	1X
	Complete Endothelial Cell Medium /w Kit	Cell Biologics	H1168	1X
	EndoGRO-MV Complete Culture Media Kit	Millipore Sigma	SCME004	1X
	Penicillin-Streptomycin Solution, 100x	Corning	30-002-CI	1X
	Gelatin	Millipore Sigma	ES-006-B	0.10%
	0.05% Trypsin-EDTA	Gibco	25300054	1x
	Ruxolitinib	Selleck Chem	S1378	2 µM
	Cycloheximide	Sigma	357420010	5 µg/ml
	DMSO	Millipore Sigma	472301	<0.1%
	RU-521	Cayman Chemicals	31765	10 µM
	2'3' cGAMP	Cayman Chemicals	19887	30 µg/ml
	Recombinant Human IFN-alpha 2	R&D Systems	11105-1	2000 units/ml
	Recombinant Human IFN beta protein	R&D Systems	8499-IF-010/CF	500 units/ml
	TNF	R&D Systems	210-TA-020	20 ng/ml
	Lipopolysaccharides	Sigma Aldrich	L4391	1 µg/ml
	Endothelial Cell Growth Supplement	Discovery Labware	356006	5 mg/mL
	Heparin sodium salt from porcine intestinal mucosa	Sigma	H3393	10 mg/mL
	Sodium Pyruvate (100mM)	Gibco	11360-070	1%
	GlutaMAX (100X)	Gibco	A12860-01	1%
	MEM Non-Essential Amino Acids (100X)	Gibco	11140-050	1%
	HEPES (1M)	GIbco	15630-080	2.50%
	Dynabeads Sheep anti-Rat igG	Invitrogen	11035	0.5 mg/mL
Gel Electrophoresis and Immunoblotting	Complete protease inhibitor mixture	Roche Applied	11697498001	1X

	PhosSTOP phosphatase inhibitor mixture	Roche Applied	4906845001	1X
	Sodium fluoride	Millipore Sigma	S-1504	100 mM
	Phenyl arsine oxide	Millipore Sigma	P-3075	100 µM
	Sodium pyrophosphate decahydrate	Millipore Sigma	221368	10 mM
	Sodium orthovanadate	Millipore Sigma	S6508	100 µM
	Transblot Turbo RTA Mini Nitrocellulose - Transfer kit	Bio-Rad	1704270	N/A
	Clarity Western ECL Substrate	Bio-Rad	1705061	As per instructions
	Clarity Max Western ECL Substrate	Bio-Rad	1705062	As per instructions
	Bovine Serum Albumin	Rockland Immunochemicals	BSA-1000	1-5%
**Immuno-fluorescence**	4′,6-Diamidine-2′-phenylindole dihydrochloride (DAPI)	Roche	10236276001	1 µg/ml
	Triton X	Sigma	T8787	0.1%
**RNA isolation and RTqPCR**	PrimeScript RT Master Mix	Clontech	RR036B	1X
	TRIzol reagent	Invitrogen	15596018	1X
	iTaq Universal SYBR Green Supermix	Bio Rad	1725125	1X
	Rneasy Plus Micro Kit	Qiagen	74034	N/A
**siRNA**	Lipofectamine RNAiMAX transfection reagent	Invitrogen	13778150	6 pmol siRNA/µl lipid
	ON-TARGETplus non-targeting control pool	Horizon Discovery	D-001810-10-20	50 nM
	Opti-MEM	Thermo Fisher Scientific	31985070	1X
	ON-TARGETplus Human STAT1 siRNA, smartpool	Horizon Discovery Biosciences	L-003543-00-0005	50 nM
	ON-TARGETplus Human STAT3 siRNA, smartpool	Horizon Discovery Biosciences	L-003544-00-0005	50 nM
	ON-TARGETplus Human IFNAR1 siRNA, smartpool	Horizon Discovery Biosciences	L-020209-00-0020	50 nM
	ON-TARGETplus Human TMEM173 (STING) siRNA, smartpool	Horizon Discovery Biosciences	L-024333-00-0005	50 nM
	ON-TARGETplus Human IRF1 siRNA, smartpool	Horizon Discovery Biosciences	L-011704-00-0020	50 nM
	ON-TARGETplus Human IRF2 siRNA, smartpool	Horizon Discovery Biosciences	L-019668-00-0020	50 nM
	ON-TARGETplus Human IRF3 siRNA, smartpool	Horizon Discovery Biosciences	L-006875-00-0005	50 nM
	ON-TARGETplus Human IRF4 siRNA, smartpool	Horizon Discovery Biosciences	L-011705-02-0020	50 nM
	ON-TARGETplus Human TMEM173 (CGAS) siRNA, smartpool	Horizon Discovery Biosciences	L-015607-02-0010	50 nM
	On Target plus non-targeting control pool	Horizon Discovery Biosciences	D-001810-10-20	50 nM
**TRAP**	Avanti Polar Lipids, 1,2-diheptanlyl-sn-glycerol	Avanti Polar Lipids	NC9999043	300 mM
	RNAsin Plus	Promega	PAN2615	40 U/ml
	Protein G beads	Thermo Fisher/Invitrogen	10004D	375 µl
	Ab for TRAP	Memorial Sloan Kettering CC	HTZGFP-19C8	50 µg

	Ab for TRAP	Memorial Sloan Kettering CC	HTZGFP-19F7	50 µg
	HBSS no calcium, mg, phenol red	Gibco	14175079	1X
	Complete mini EDTA free easy pack	Roche	4693159001	1X
	Cyclohexamide	Sigma	C1988	100 µg/m
	DTT-biotech grade	VWR Life Sciences	97061-340	0.5 mM
	Potassium Chloride ACS	VWR Life Sciences	97061-566	350 mM
	HEPES Free Biotechnology grade	Sigma	H4034	20 mM
	Magnesium Chloride	Honeywell	63020	5 mM
	Diethyl pyrocarbonate (DEPC), 5ml	Sigma	D5758	1X
	Sodium Bicarbonate	Fisher Scientific	S233	4 mM
	(D) + Glucose	Acros	41095	35 mM
	Igepal	MP Biomedical	CA-630	1X
	Disposable pellet pestles with 1.5ml microtube	DWK Life Sciences	749520-0000	N/A
	Cordless Pestle Motor	VWR Life Sciences	47747-370	N/A
	Diamond Midi Centrifuge Tube, 5.0mL, PP, Separate Red Screw Cap, Graduated	Globe Scientific	111580	N/A
	RNeasy Plus Micro Kit	Qiagen	74034	N/A
	RNAse Away	Molecular Bioproducts	7002	1X
**ELISA**	IP10 (CXCL10) Mouse Elisa kit	Thermo Fisher	BMS6018	As per instructions
**Lentivirus production**	HEK293FT	Invitrogen	R70007	N/A
	pCMV-dR8.91	Addgene	8455	67 µg
	pCMV-VSVG	Addgene	8454	33 µg
	Vivacell 100, 30,000 MWCO PES, 10pc	Sartorius	VC1022	N/A
**Cell fractionalion**	NE-PER™ Nuclear and Cytoplasmic Extraction Reagents	Thermo Fisher	78833	N/A

Supplemental Table 2 - Antibodies

Target	Species	Clone or isotype	Conjugate	Vendor	Catalog No	RRID
**pY701-STAT1**	rabbit	clone 58D6		Cell Signaling	9167	AB_561284
**STAT1**	rabbit	clone D1K9Y		Cell Signaling	14994	AB_2737027
**pY690-STAT2**	rabbit	clone D3P2P		Cell Signaling	88410	AB_2800123
**STAT2**	rabbit	clone D9J7L		Cell Signaling	72604	AB_2799824
**pY705-STAT3**	rabbit	clone D3A7		Cell Signaling	9145	AB_2491009
**STAT3**	rabbit	clone D1A5		Cell Signaling	8768	AB_2722529
**STING**	rabbit	clone D2P2F		Cell Signaling	13647	AB_2732796
**CGAS**	rabbit	clone E5V3W		Cell Signaling	79978	AB_2905508
**Tubulin**	mouse	clone DM1A		Santa Cruz	sc-322939	AB_628412
**Lamin A/C**	rabbit	polyclonal		Proteintech	10298-1-AP	AB_2296961
**β-Actin**	mouse	clone AC-15		Sigma	A5441	AB_476744
**Ve-Cadherin**	goat	polyclonal		R&D Systems	AF1002	AB_2077789
**pS396-IRF3**	rabbit	clone D6O1M		Cell Signaling	29047	AB_2773013
**IRF3**	rabbit	clone D6I4C		Cell Signaling	11904	AB_2722521
**IRF9**	rabbit	clone D2T8M		Cell Signaling	76684	AB_2799885
**HTZGFP**	mouse	clone 19C8		Memorial Sloan Kettering Antibody & BioResource Core	HTZGFP-19C8	AB_2716737
**HTZGFP**	mouse	clone 19F7		Memorial Sloan Kettering Antibody & BioResource Core	HTZGFP-19F7	AB_2716736
**CD326**	rat	clone G8.8	Biotin	Biolegend	118204	AB_1134178
**CD45**	rat	clone 30-F11	Biotin	Biolegend	103104	AB_312969
**CD31**	rat	clone MEC13.3	Biotin	Biolegend	102504	AB_312911
**CD31**	rat	clone MEC13.3		BioLegend	102502	AB_312909
**CD102**	rat	clone 3C4(MIC2/4)		BioLegend	105602	AB_313195
**CXCL10**	rabbit	clone D5L5L		Cell Signaling	14969	AB_2798668
**mouse IgG**	goat	polyclonal	HRP	Jackson Immunoresearch	115-035-062	AB_2338504
**rabbit IgG**	goat	polyclonal	HRP	Jackson Immunoresearch	111-035-003	AB_2313567
**rabbit IgG**	donkey	polyclonal	Alexa Fluor 647	Invitrogen	A-31573	AB_2536183
**goat IgG**	donkey	polyclonal	Alexa Fluor 594	Invitrogen	A-11058	AB_2534105
**rabbit IgG**	goat	polyclonal	Biotin	Vector Labs	BA-1000	AB_2313606

Supplemental Table 3

Primers for RT-qPCR

Gene	Forward	Reverse
CXCL10	GGTGAGAAGAGATGTCTGAATCC	GTCCATCCTTGGAAGCACTGCA
CXCL2	GGCAGAAAGCTTGTCTCAACCC	CTCCTTCAGGAACAGCCACCAA
DDX58	CACCTCAGTTGCTGATGAAGGC	GTCAGAAGGAAGCACTTGCTACC
IFIH1	GCTGAAGTAGGAGTCAAAGCCC	CCACTGTGGTAGCGATAAGCAG
IFIT3	CCTGGAATGCTTACGGCAAGCT	GAGCATCTGAGAGTCTGCCCAA
IFNAR1	CGCCTGTGATCCAGGATTATCC	TGGTGTGTGCTCTGGCTTTCAC
IL6	TACCACTTCACAAGTCGGAGGC	CTGCAAGTGCATCATCGTTGTTC
IRF1	GAGGAGGTGAAAGACCAGAGCA	TAGCATCTCGGCTGGACTTCGA
IRF2	TAGAGGTGACCACTGAGAGCGA	CTCTTCATCGCTGGGCACACTA
IRF3	TCTGCCCTCAACCGCAAAGAAG	TACTGCCTCCACCATTGGTGTC
IRF4	GAACGAGGAGAAGAGCATCTTCC	CGATGCCTTCTCGGAACTTTCC
MX1	GGCTGTTTACCAGACTCCGACA	CACAAAGCCTGGCAGCTCTCTA
OAS1	AGGAAAGGTGCTTCCGAGGTAG	GGACTGAGGAAGACAACCAGGT
OAS2	GCTTCCGACAATCAACAGCCAAG	CTTGACGATTTTGTGCCGCTCG
OAS3	CCTGATTCTGCTGGTGAAGCAC	TCCCAGGCAAAGATGGTGAGGA
SOCS3	CATCTCTGTCGGAAGACCGTCA	GCATCGTACTGGTCCAGGAACT
STAT1	ATGGCAGTCTGGCGGCTGAATT	CCAAACCAGGCTGGCACAATTG
STAT3	CTTTGAGACCGAGGTGTATCACC	GGTCAGCATGTTGTACCACAGG
STING	CCTGAGTCTCAGAACAACTGCC	GGTCTTCAAGCTGCCCACAGTA

Primers for genotyping

Primer name	Forward	Reverse
Cdh5CreERT2	ACCAGCCAGCTATCAACTCG	TTACATTGGTCCAGCCACC
Rosa26fsTRAP	CTACAACAGCCACAACGTCTA	CAAACACAGCACCTTCTTCATC
SOCS3 flox	CGGGCAGGGGAAGAGACTGT	TCGACTGTCCTCGGTCAC
STING flox	ACACGCTCTGTTTACTATGAACCTC	GGGGGAAGGAGAGAACTGAC

Supplemental code

```
library(parallel)
library(Rsubread)
library(gplots)
library(openxlsx)
library(edgeR)
library(limma)
library(DESeq2)
library(genefilter)
library(RColorBrewer)
library(org.Mm.eg.db)
library(cluster)
library(factoextra)
library(clusterProfiler)
library(sva)
library(systemPipeR)
library(rtracklayer)
library(stringr)
library(GenomicFeatures)
library(progeny)
library(decoupleR)
library(dplyr)
library(tibble)
library(tidyr)
library(pheatmap)
library(ggplot2)
library(ggrepel)
library(ReactomePA)
library(graphite)
library(igraph)
library(ggraph)

runEdger <- function (data, sampletype="samples", contrast, topnum) {
  group <- factor(as.character(data[1,]))
  tmpdata <- data
  data <- as.data.frame(lapply(tmpdata[2:nrow(tmpdata),], as.numeric))
  rownames(data) <- rownames(tmpdata[2:nrow(tmpdata),])
  y <- DGEList(counts=data, group=group)
  keep<-filterByExpr(y)
  y<-y[keep,,keep.lib.sizes=FALSE]
  y<-normLibSizes(y)
  design<-model.matrix(~0+group,data=y$samples)
  y<-estimateDisp(y,design)
  v <- voom(y,design)
  fit <- lmFit(v)

  if (contrast=="LPSvsSaline") {
    cont.matrix <- makeContrasts(LPSvsSaline = group3_Oil_LPS - group1_Oil_Saline, levels=design) }
  else if (contrast=="LPS_KOvsWT") {
    cont.matrix <- makeContrasts(LPS_KOvsWT = group4_TMX_LPS - group3_Oil_LPS, levels=design) }
  else if (contrast=="Saline_KOvsWT") {
    cont.matrix <- makeContrasts(Saline_KOvsWT = group2_TMX_Saline - group1_Oil_Saline,
levels=design) }
  else if (contrast=="LPS_KOvsSaline_WT") {
    cont.matrix <- makeContrasts(LPS_KOvsSaline_WT = group4_TMX_LPS - group1_Oil_Saline,
levels=design) }
  else if (contrast=="LPS_KOvsSaline_KO") {
    cont.matrix <- makeContrasts(LPS_KOvsSaline_KO = group4_TMX_LPS - group2_TMX_Saline,
levels=design) }

  fit.cont <- contrasts.fit(fit, cont.matrix)
  fit.cont <- eBayes(fit.cont)
  dim(fit.cont)
  summa.fit <- decideTests(fit.cont)
  summary(summa.fit)
  top <- topTable(fit.cont, sort.by = "P", adjust="BH", n = topNumber)
  topAll <- merge(top, data, by = 'row.names')
  topAll <- topAll[order(-topAll$logFC),]
  colnames(topAll)[1] <- "SYMBOL"
  rownames(topAll) <- topAll$SYMBOL
  write.csv(topAll, file=paste(sampletype,"_",contrast,"_top",topnum,".csv", sep=""),row.names =
FALSE)
```

```
  counts <- topAll[,8:ncol(topAll)]
  counts <- t(apply(counts, 1, cal_z_score))
  pal_breaks <- c(seq(min(counts)*0.9, 0, length.out = 33),
                  seq(0.03, 1, length.out = 33),
                  seq(1.03, max(counts)*0.9, length.out = 34))
  if (!is.null(dev.list())) {dev.off()}
  pdf(paste(sampletype,"_",contrast,"_heatmap_top",topnum,".pdf", sep=""), useDingbats=FALSE)
  pheatmap(counts, cluster_rows = FALSE, cluster_cols = FALSE, fontsize = 8, angle_col = 90,
           cellwidth=10, cellheight = 0.75, show_rownames = FALSE, show_colnames = TRUE,
           color = pal, breaks = pal_breaks)
  dev.off()

  top$diffexpressed <- "NO"
  top$diffexpressed[top$logFC > log2(2) & top$P.Value < 0.05] <- "UP"
  top$diffexpressed[top$logFC < log2(0.5) & top$P.Value < 0.05] <- "DOWN"

  pdf(paste(sampletype,"_",contrast,"_volcano.pdf", sep=""), useDingbats=FALSE)
  print(
    ggplot(data = top, aes(x = logFC, y = -log10(P.Value), col = diffexpressed)) +
      theme_classic() +
      geom_vline(xintercept = c(log2(0.5), log2(2)), col = "gray", linetype = 'dashed') +
      geom_hline(yintercept = -log10(0.05), col = "gray", linetype = 'dashed') +
      geom_point(size = 0.5) +
      scale_color_manual(values = c("#0000FF", "#BBBBBB", "#FF0000"), labels = c("Downregulated",
"Not significant", "Upregulated")) +
      labs(color = sampletype, x = expression("log"[2]*"(FC)"), y = expression("-log"[10]*"(P)")) +
      scale_x_continuous(expand = c(0, 0)) +
      scale_y_continuous(expand = c(0, 0))
  )
  dev.off()


  return (topAll)
}

runGSEA <- function (top, sampletype="samples") {
  top <- top[order(-top$logFC),]
  geneList <- top$logFC
  names(geneList) <- rownames(top)
  gse1 <- clusterProfiler::gseGO(geneList = geneList,
                                 ont = "BP",
                                 keyType = "SYMBOL",
                                 minGSSize = 3,
                                 maxGSSize = 800,
                                 pvalueCutoff = 0.05,
                                 verbose = TRUE,
                                 OrgDb = org.Mm.eg.db,
                                 pAdjustMethod = "BH")

  gse_results1 <- enrichplot::dotplot(gse1, showCategory=20)
  if (!is.null(dev.list())) {dev.off()}
  pdf(paste(sampletype,"_",contrast,"_GSEA.pdf", sep=""), useDingbats=FALSE)
  print(gse_results1)
  dev.off()
  write.csv(gse1, paste(sampletype,"_",contrast,"_GSEA.csv"), quote=F)
  return (gse1)
}

runDecouplerPathways <- function (sampletype="samples", data, top, treatConditions, contrast) {
  tmpdata <- data
  data <- as.data.frame(lapply(tmpdata[3:nrow(tmpdata),], as.numeric))
  rownames(data) <- rownames(tmpdata[3:nrow(tmpdata),])

  activities <- run_wmean(mat=data, net=net, .source='source', .target='target',
                          .mor='weight', times = 100, minsize = 5)
  activities_mat <- activities %>%
    filter(statistic == 'norm_wmean') %>%
    pivot_wider(id_cols = 'condition', names_from = 'source',
                values_from = 'score') %>%
    column_to_rownames('condition') %>%
    as.matrix()
  activities_mat <- scale(activities_mat)
```

```
  palette_length = 100
  my_color = colorRampPalette(c("Darkblue", "white","red"))(palette_length)

  my_breaks <- c(seq(-3, 0, length.out=ceiling(palette_length/2) + 1),
                 seq(0.05, 3, length.out=floor(palette_length/2)))
  if (!is.null(dev.list())) {dev.off()}
  pdf(paste(sampletype,"_",contrast,"_PathwaysHM.pdf", sep=""), useDingbats=FALSE)
  pheatmap(activities_mat, border_color = NA, color=my_color, breaks = my_breaks)
  dev.off()

  deg <- as.matrix(top$t)
  rownames(deg) <- rownames (top)
  contrast_acts <- run_wmean(mat=deg, net=net, .source='source', .target='target',
                             .mor='weight', times = 100, minsize = 5)
  f_contrast_acts <- contrast_acts %>%
    filter(statistic == 'norm_wmean')
  if (!is.null(dev.list())) {dev.off()}
  pdf(paste(sampletype,"_",contrast,"_Pathways.pdf", sep=""), useDingbats=FALSE)
  print (
    ggplot(f_contrast_acts, aes(x = reorder(source, score), y = score)) +
      geom_bar(aes(fill = score), stat = "identity") +
      scale_fill_gradient2(low = "darkblue", high = "indianred",
                           mid = "whitesmoke", midpoint = 0) +
      theme_minimal() +
      theme(axis.title = element_text(face = "bold", size = 12),
            axis.text.x =
              element_text(angle = 45, hjust = 1, size =10, face= "bold"),
            axis.text.y = element_text(size =10, face= "bold"),
            panel.grid.major = element_blank(),
            panel.grid.minor = element_blank()) +
      xlab("Pathways"))
  dev.off()

  pathway <- 'JAK-STAT'

  df <- net %>%
    filter(source == pathway) %>%
    arrange(target) %>%
    mutate(ID = target, color = "3") %>%
    column_to_rownames('target')
  inter <- sort(intersect(rownames(deg),rownames(df)))
  df <- df[inter, ]
  df['t_value'] <- deg[inter, ]
  df <- df %>%
    mutate(color = if_else(weight > 0 & t_value > 0, '1', color)) %>%
    mutate(color = if_else(weight > 0 & t_value < 0, '2', color)) %>%
    mutate(color = if_else(weight < 0 & t_value > 0, '2', color)) %>%
    mutate(color = if_else(weight < 0 & t_value < 0, '1', color))

  if (!is.null(dev.list())) {dev.off()}
  pdf(paste(sampletype,"_",contrast,"_",pathway,".pdf", sep=""), useDingbats=FALSE)
  print (
    ggplot(df, aes(x = weight, y = t_value, color = color)) + geom_point() +
      scale_colour_manual(values = c("red","royalblue3","grey")) +
      geom_label_repel(aes(label = ID)) +
      theme_minimal() +
      theme(legend.position = "none") +
      geom_vline(xintercept = 0, linetype = 'dotted') +
      geom_hline(yintercept = 0, linetype = 'dotted') +
      ggtitle(pathway))
  dev.off()
}

runDecouplerTFs <- function (sampletype="samples", data, top, treatConditions, contrast, n_tfs, tf =
"STAT1") {
  dataUp <- top
  rownames(dataUp) <- toupper(rownames(dataUp))

  tmpdata <- data
  data <- as.data.frame(lapply(tmpdata[3:nrow(tmpdata),], as.numeric))
  rownames(data) <- rownames(tmpdata[3:nrow(tmpdata),])
  rownames(data) <- toupper(rownames(data))
```

```
  sample_acts <- run_wmean(mat=data, net=netTF, .source='source', .target='target',
                           .mor='mor', times = 100, minsize = 5)
  sample_acts_mat <- sample_acts %>%
    filter(statistic == 'norm_wmean') %>%
    pivot_wider(id_cols = 'condition', names_from = 'source',
                values_from = 'score') %>%
    column_to_rownames('condition') %>%
    as.matrix()

  # Get top tfs with more variable means across clusters
  tfs <- sample_acts %>%
    group_by(source) %>%
    summarise(std = sd(score)) %>%
    arrange(-abs(std)) %>%
    head(n_tfs) %>%
    pull(source)
  sample_acts_mat <- sample_acts_mat[,tfs]

  # Scale per sample
  sample_acts_mat <- scale(sample_acts_mat)

  # Choose color palette
  palette_length = 100
  my_color = colorRampPalette(c("Darkblue", "white","red"))(palette_length)

  my_breaks <- c(seq(-3, 0, length.out=ceiling(palette_length/2) + 1),
                 seq(0.05, 3, length.out=floor(palette_length/2)))

  if (!is.null(dev.list())) {dev.off()}
  pdf(paste(sampletype,"_",contrast,"_TFsHM.pdf", sep=""), useDingbats=FALSE)
  pheatmap(sample_acts_mat, border_color = NA, color=my_color, breaks = my_breaks)
  dev.off()

  contrast_acts <- run_wmean(mat=dataUp[, 't', drop=FALSE], net=netTF, .source='source',
.target='target',
                             .mor='mor', times = 100, minsize = 5)
  f_contrast_acts <- contrast_acts %>%
    filter(statistic == 'norm_wmean') %>%
    mutate(rnk = NA)
  msk <- f_contrast_acts$score > 0
  f_contrast_acts[msk, 'rnk'] <- rank(-f_contrast_acts[msk, 'score'])
  f_contrast_acts[!msk, 'rnk'] <- rank(-abs(f_contrast_acts[!msk, 'score']))
  tfs <- f_contrast_acts %>%
    arrange(rnk) %>%
    head(n_tfs) %>%
    pull(source)
  f_contrast_acts <- f_contrast_acts %>%
    filter(source %in% tfs)

  if (!is.null(dev.list())) {dev.off()}
  pdf(paste(sampletype,"_",contrast,"_TFs.pdf", sep=""), useDingbats=FALSE)
  print (
    ggplot(f_contrast_acts, aes(x = reorder(source, score), y = score)) +
      geom_bar(aes(fill = score), stat = "identity") +
      scale_fill_gradient2(low = "darkblue", high = "indianred",
                           mid = "whitesmoke", midpoint = 0) +
      theme_minimal() +
      theme(axis.title = element_text(face = "bold", size = 12),
            axis.text.x =
              element_text(angle = 45, hjust = 1, size =10, face= "bold"),
            axis.text.y = element_text(size =10, face= "bold"),
            panel.grid.major = element_blank(),
            panel.grid.minor = element_blank()) +
      xlab("Pathways"))
  dev.off()

  df <- netTF %>%
    filter(source == tf) %>%
    arrange(target) %>%
    mutate(ID = target, color = "3") %>%
    column_to_rownames('target')
```

```
  inter <- sort(intersect(rownames(dataUp),rownames(df)))
  df <- df[inter, ]
  df[,c('logfc', 't_value', 'p_value')] <- dataUp[inter, c(2,4,6)]
  df <- df %>%
    mutate(color = if_else(mor > 0 & t_value > 0, '1', color)) %>%
    mutate(color = if_else(mor > 0 & t_value < 0, '2', color)) %>%
    mutate(color = if_else(mor < 0 & t_value > 0, '2', color)) %>%
    mutate(color = if_else(mor < 0 & t_value < 0, '1', color))

  if (!is.null(dev.list())) {dev.off()}
  pdf(paste(sampletype,"_",contrast,"_",tf,".pdf", sep=""), useDingbats=FALSE)
  print (
    ggplot(df, aes(x = logfc, y = -log10(p_value), color = color, size=abs(mor))) +
      geom_point() +
      scale_colour_manual(values = c("red","royalblue3","grey")) +
      geom_label_repel(aes(label = ID, size=1)) +
      theme_minimal() +
      theme(legend.position = "none") +
      geom_vline(xintercept = 0, linetype = 'dotted') +
      geom_hline(yintercept = 0, linetype = 'dotted') +
      ggtitle(tf))
  dev.off()

}

runReactome <- function (top, sampletype="samples") {
  top <- top[order(-top$logFC),]
  geneList <- top$logFC
  symbols <- rownames(top)
  eg = clusterProfiler::bitr(symbols, fromType="SYMBOL", toType="ENTREZID", OrgDb="org.Mm.eg.db")
  names(geneList) <- eg$ENTREZID
  y <- gsePathway(geneList,
                  organism = "mouse",
                  pvalueCutoff = 0.2,
                  pAdjustMethod = "BH",
                  verbose = FALSE)
  head(y)
  gse_results1 <- enrichplot::dotplot(y, showCategory=20)
  if (!is.null(dev.list())) {dev.off()}
  pdf(paste(sampletype,"_",contrast,"_Reactome.pdf", sep=""), useDingbats=FALSE)
  print(gse_results1)
  dev.off()
  write.csv(y, paste(sampletype,"_",contrast,"_Reactome.csv"), quote=F)
  return (y)
}

viewPathways <- function (pathwayNumber = 1, top, reactome, sampletype="samples") {
  print (reactome$Description[pathwayNumber])
  core_enr <- as.vector(strsplit(reactome$core_enrichment[pathwayNumber], "\\/")[[1]])
  symbols = clusterProfiler::bitr(core_enr, fromType="ENTREZID", toType=c("SYMBOL", "GENENAME"),
OrgDb="org.Mm.eg.db")
  rownames(symbols) <- symbols$SYMBOL
  fc <- merge(symbols, y, by = 'row.names')$logFC
  names(fc) <- core_enr
  fc2 <- merge(symbols, y, by = 'row.names')
  fc2 <- fc2[,c(9,6,3)]
  colnames(fc2) <- c("pvalue","logFC","gene")
  fc3 = fc2$logFC
  names(fc3) <- fc2$gene
  print(
    viewPathway2(sampletype, reactome$Description[pathwayNumber],
                 organism = "mouse",
                 readable = TRUE,
                 foldChange = fc3)
  )
}

viewPathway2 <- function(sampletype,
                         pathName,
                         organism="human",
                         readable=TRUE,
```

```
                         foldChange=NULL,
                         keyType = "ENTREZID",
                         layout = "kk"){
  print (pathName)
  org2org <- list(arabidopsis="athaliana",
                  bovine="btaurus",
                  canine="cfamiliaris",
                  chicken="ggallus",
                  ecolik12="ecoli",
                  fly="dmelanogaster",
                  human="hsapiens",
                  mouse="mmusculus",
                  pig="sscrofa",
                  rat="rnorvegicus",
                  celegans="celegans",
                  xenopus="xlaevis",
                  yeast="scerevisiae",
                  zebrafish="drerio")

  if(!(organism %in% names(org2org))){
    cat(paste(c("the list of supported organisms:",names(org2org)), collapse='\n'))
    stop(sprintf("organism %s is not supported", organism))
  }
  pathways <- eval(parse(text="pathways"))
  p <- pathways(org2org[[organism]], 'reactome')[[pathName]]

  p <- convertIdentifiers(p, "symbol")

  g <- pathwayGraph(p)
  gg <- igraph.from.graphNEL(g)
  gg <- as.undirected(gg)
  gg <- setting.graph.attributes(gg)
  V(gg)$name <- sub("[^:]+:", "", V(gg)$name)

  if (!is.null(foldChange)) {
    fch <- foldChange[V(gg)$name]
    V(gg)$color <- fch

  }
  print(
    ggraph(gg, layout=layout) +
      geom_edge_link(alpha=.8, colour='darkgrey') +
      geom_node_point(aes_(color=~as.numeric(as.character(color)), size=~size)) +
      scale_color_continuous(low="red", high="blue", name = "fold change", na.value = "#E5C494") +
      geom_node_text(aes_(label=~name), repel=TRUE, max.overlaps=Inf) +
      ## scale_color_gradientn(name = "fold change", colors=palette, na.value = "#E5C494") +
      scale_size(guide = "none") + theme_void()
  )
  names <- V(gg)$name
  fold <- foldChange[V(gg)$name]
  genelist <- as.data.frame(cbind(names, fold))
  genelist <- genelist[!is.na(genelist$fold),]
  write.csv(genelist, paste(sampletype,"_",contrast,"_Reactome_", pathName, ".csv"), quote=F)
  print (paste(sampletype,"_",contrast,"_Reactome_", pathName, ".csv"))
}

setting.graph.attributes <- function(g, node.size=8,
                                     node.color="#B3B3B3",
                                     edege.width=2,
                                     edege.color="#8DA0CB") {
  V(g)$size <- node.size
  V(g)$color <- node.color
  V(g)$label <- V(g)$name

  E(g)$width <- edege.width
  E(g)$color <- edege.color

  return(g)
}

plotReactomeHeatmap <- function (pathwayNumber, y, reactome) {
  print (reactome$Description[pathwayNumber])
```

```
  reactome$Description[pathwayNumber]
  core_enr <- as.vector(strsplit(reactome$core_enrichment[pathwayNumber], "\\/")[[1]])
  symbols = clusterProfiler::bitr(core_enr, fromType="ENTREZID", toType=c("SYMBOL", "GENENAME"),
OrgDb="org.Mm.eg.db")
  rownames(symbols) <- symbols$SYMBOL
  counts <- merge (symbols, y, by = 'row.names')
  rownames(counts) <- counts$SYMBOL.x
  counts <- counts[,12:ncol(counts)]
  counts <- t(apply(counts, 1, cal_z_score))
  print(colSums(counts))
  pal_breaks <- c(seq(min(counts)*0.9, 0, length.out = 33),
                  seq(0.03, 1, length.out = 33),
                  seq(1.03, max(counts)*0.9, length.out = 34))
  pheatmap(counts, cluster_rows = FALSE, cluster_cols = FALSE, fontsize = 8, angle_col = 90,
           cellwidth=10, cellheight = 0.75, show_rownames = FALSE, show_colnames = TRUE,
           color = pal, breaks = pal_breaks)
}

plotGOTermHeatmap <- function (GOTable, GOterm, sampletype, y) {
  GOTable <- GOTable[!duplicated(GOTable$SYMBOL),]
  GOTable <- GOTable[!is.na(GOTable$SYMBOL),]
  rownames(GOTable) <- GOTable$SYMBOL
  GOTable2 <- merge(GOTable, y, by = 'row.names')
  GOTable2 <- GOTable2[,7:ncol(GOTable2)]
  GOTable3 <- GOTable2[GOTable2$adj.P.Val<0.05,]

  counts <- GOTable3[,8:ncol(GOTable3)]
  rownames(counts) <- GOTable3$SYMBOL
  counts <- t(apply(counts, 1, cal_z_score))
  print(colSums(counts))
  pal_breaks <- c(seq(min(counts)*0.9, 0, length.out = 33),
                  seq(0.03, 1, length.out = 33),
                  seq(1.03, max(counts)*0.9, length.out = 34))
  pheatmap(counts, cluster_rows = TRUE, cluster_cols = FALSE, fontsize = 8, angle_col = 90,
           cellwidth=10, cellheight = 0.75, show_rownames = FALSE, show_colnames = TRUE,
           color = pal, breaks = pal_breaks)
  write.csv(GOTable3, paste(sampletype,"_",contrast,"_GO_", GOterm, ".csv"), quote=F)
}

cal_z_score <- function(x){ (x - mean(x)) / sd(x) }
#Identify many cores have to use
detectCores()
#Puget computer has 128 cores to use

# Read the sample information into R
sampleTable <- read.csv('metadata_STING.csv', header = TRUE, sep = ",")
sampleTable

#Upload to environment FASTQ files
reads1 <- list.files(path = "./01_trim", pattern = "R1_001_val_1.fq.gz$", full.names = TRUE)
reads2 <- list.files(path = "./01_trim", pattern = "R2_001_val_2.fq.gz$", full.names = TRUE)

align( index          = "~/bioinf/Core/1_index_rnaseq/mm10_index/mm10",
       readfile1      = reads1,
       readfile2      = reads2,
       type           = 'rna',
       input_format   = "gzFASTQ",
       output_format  = "BAM",
       PE_orientation = 'fr',
       nthreads       = 64 ) #use the parallel to identify how many cores have to use

#Have a variable with all the bam files
bam.files <- list.files(path = "./02_bam", pattern = ".BAM$", full.names = TRUE)
bam.files

#The function propmapped returns the proportion of mapped reads in the output SAM file:
#total number of input reads, number of mapped reads and proportion of mapped reads.
props <- propmapped(files=bam.files)
props

#Rsubread provides a read summarization function featureCounts, which takes two inputs:
# 1. the aligned reads (BAM or SAM) and assigns them to
```

```
# 2. genomic features (GTF annotation file)
seqdata <- featureCounts( bam.files, useMetaFeatures = TRUE, annot.inbuilt = "mm10", isPairedEnd =
TRUE,
                          nthreads = 64)

#matrix with the counts
gene.counts <- seqdata$counts
#Save the Ids for the lines
gene.ids    <- seqdata$annotation$GeneID

#Convert counts to DGEList object
y <- DGEList(gene.counts)

#Add groups for the samples
group <- paste(sampleTable$CellType)

# Convert to factor
group <- factor(group)

# Add the group information into the DGEList
y$samples$group <- group
y$samples

#build up our annotation information in a separate data frame using the select function
ann <-
AnnotationDbi::select(org.Mm.eg.db,keys=rownames(y$counts),columns=c("ENTREZID","SYMBOL","GENENAME"))

#merge data witn the annotation (ann)
y$genes <- ann

#Filtering lowly expressed genes#
#counts-per-million (CPM) above 0.5 in at least two samples
myCPM <- cpm(gene.counts)
thresh <- myCPM > 0.5
keep <- rowSums(thresh) >= 2

# Subset the rows of countdata to keep the more highly expressed genes
counts.keep <- gene.counts[keep,]

#filter the DGEList object
y <- y[keep, keep.lib.sizes=FALSE]

#Save csv file
#If analyzing sorted ECs
write.csv(y, file="Endo.csv")
#If analyzing total kidney
write.csv(y, file="Total.csv")




pal = colorRampPalette(c("#0000FF", "#FFFFFF", "#FFFF00", "#FF0000"))(100)


net <- get_progeny(organism = 'mouse', top = 100)
netTF <- get_collectri(organism='mouse', split_complexes=FALSE)

setwd("D:/OneDrive - Albany Medical Center/Projects/RNA-Seq/STING KO/9-18-24")

treatConditions <-read.csv("decoupleRconditions.csv", header = T)

#For STINGiEKO kidneys
data_ke <-read.csv("Endo.csv", header = T, row.names="SYMBOL")
data_wk <-read.csv("Total.csv", header = T, row.names="SYMBOL")

#For SOCS3iEKO TRAP
data_bt <-read.csv("BrainTRAP_LPS15h.csv", header = T, row.names="SYMBOL")
data_wb <-read.csv("WholeBrain_LPS15h.csv", header = T, row.names="SYMBOL")
data_kt <-read.csv("KidneyTRAP_LPS15h.csv", header = T, row.names="SYMBOL")
data_wk <-read.csv("WholeKidney_LPS15h.csv", header = T, row.names="SYMBOL")

#Choose a max number of top genes
```

```
topNumber = 100
topNumber = Inf

#Choose 1:
name <- "KidneyEndo"
data <- data_ke

name <- "WholeKidney"
data <- data_wk

data <- data_bt
name <- "BrainTRAP"

data <- data_wb
name <- "WholeBrain"

data <- data_kt
name <- "KidneyTRAP"

data <- data_wk
name <- "WholeKidney"
#From all the above

#Choose 1:
contrast <- "LPSvsSaline"
contrast <- "LPS_KOvsWT"
contrast <- "Saline_KOvsWT"
contrast <- "LPS_KOvsSaline_WT"
contrast <- "LPS_KOvsSaline_KO"
#From all the above

y <- runEdger(data, name, contrast, topNumber)
gsea <- runGSEA (y, name)
runDecouplerPathways (name, data, y, treatConditions, contrast)
runDecouplerTFs (name, data, y, treatConditions, contrast, 30)

reactome <- runReactome (y, name)
#choose pathway number from reactome result table
viewPathways (1, y, reactome, name)
plotReactomeHeatmap (1, y, reactome)

ISGlist <- (read.csv("ISG list.csv", header = F))$V1

yy <- filter(y, SYMBOL %in% ISGlist)
yy <- yy[yy$adj.P.Val<0.05,]
yy <- yy[order(yy$logFC),]
counts <- yy[,8:ncol(yy)]
counts <- t(apply(counts, 1, cal_z_score))
print(colSums(counts))
pal_breaks <- c(seq(min(counts)*0.9, 0, length.out = 33),
                seq(0.03, 1, length.out = 33),
                seq(1.03, max(counts)*0.9, length.out = 34))
pheatmap(counts, cluster_rows = FALSE, cluster_cols = FALSE, fontsize = 8, angle_col = 90,
         cellwidth=10, cellheight = 7, show_rownames = TRUE, show_colnames = TRUE,
         color = pal, breaks = pal_breaks)
write.csv(yy, "ISGlistFolds.csv")
```
