# Supplementary material for: Endothelial STING and STAT1 mediate IFN-independent effects of IL-6 in an endotoxemia-induced model of shock
Source: J Clin Invest. 2025 Sep 16;135(21):e189570. doi: 10.1172/JCI189570 (PMC12578408; doi:10.1172/JCI189570)

Uncropped blots corresponding to Figure 3B

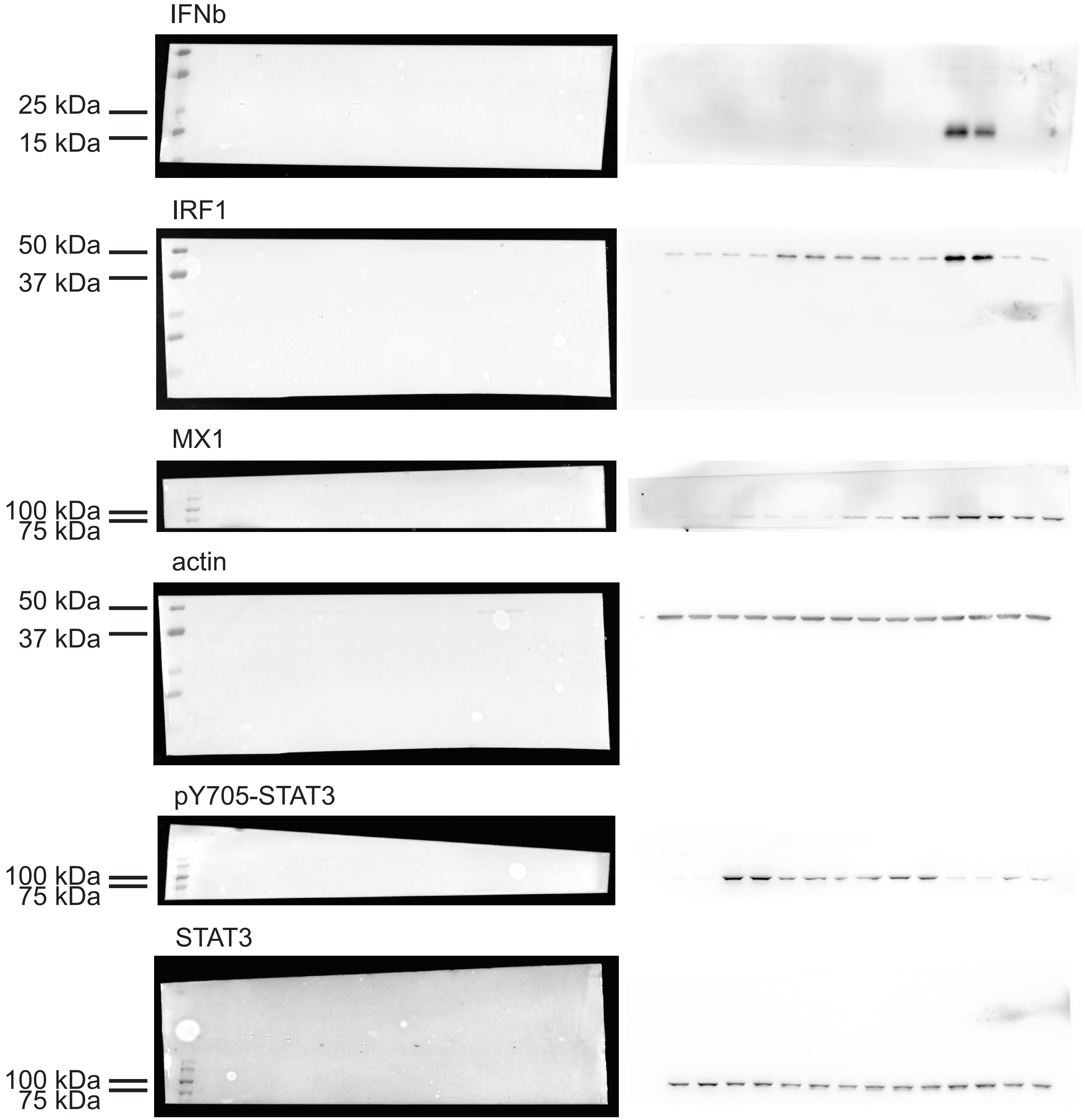

Uncropped blots corresponding to Figure 4A

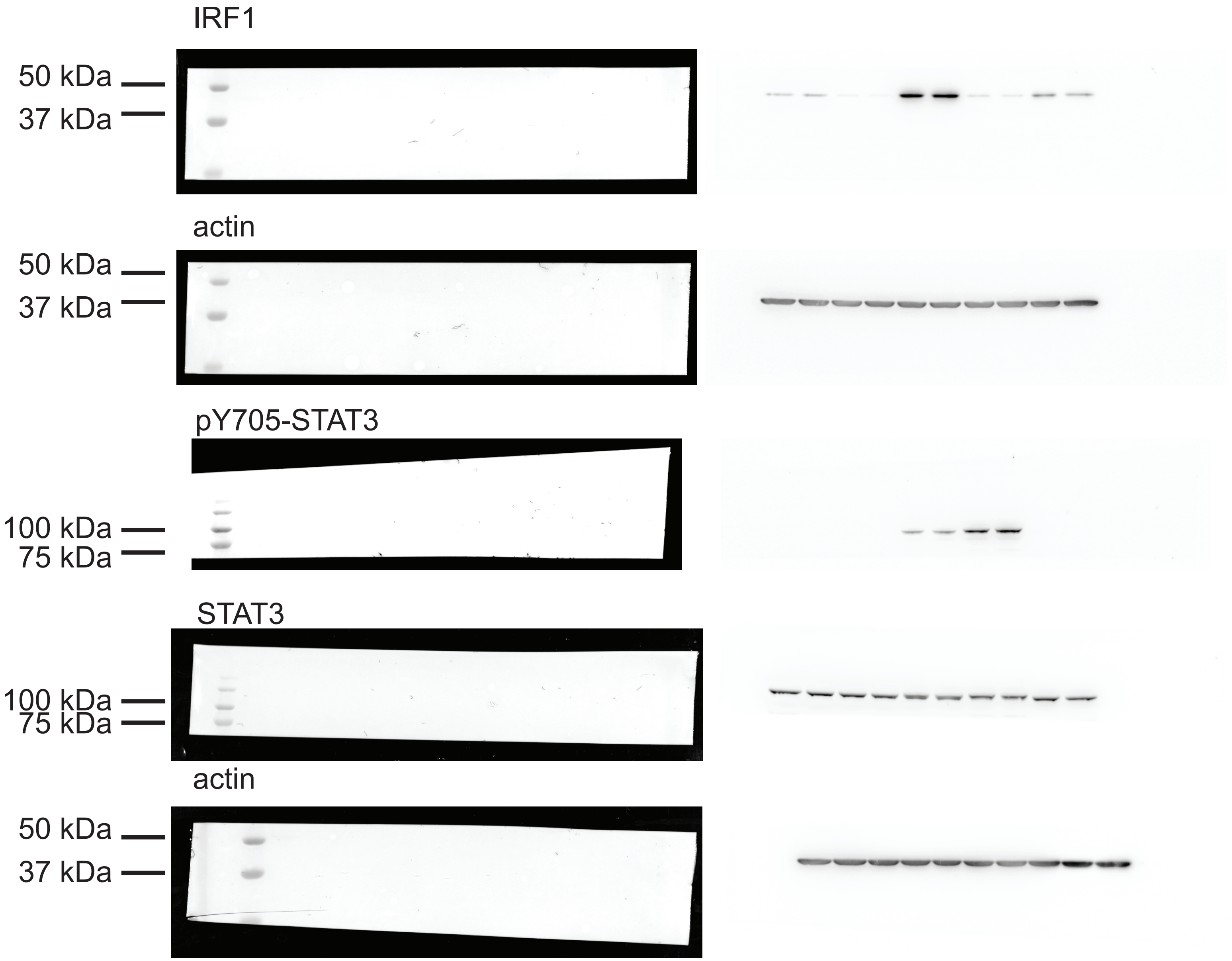

Uncropped blots corresponding to Figure 5A

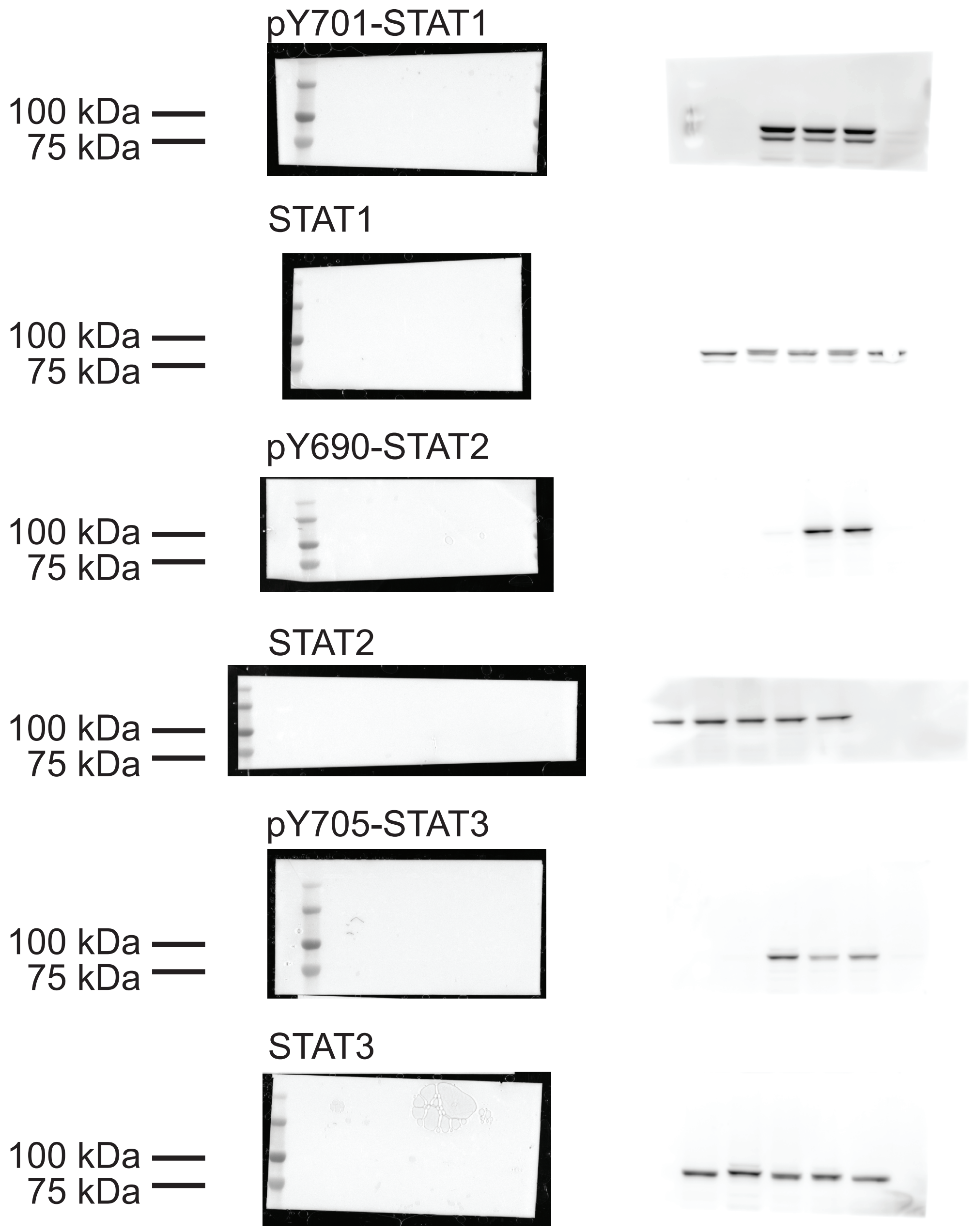

Uncropped blots corresponding to Figure 5D

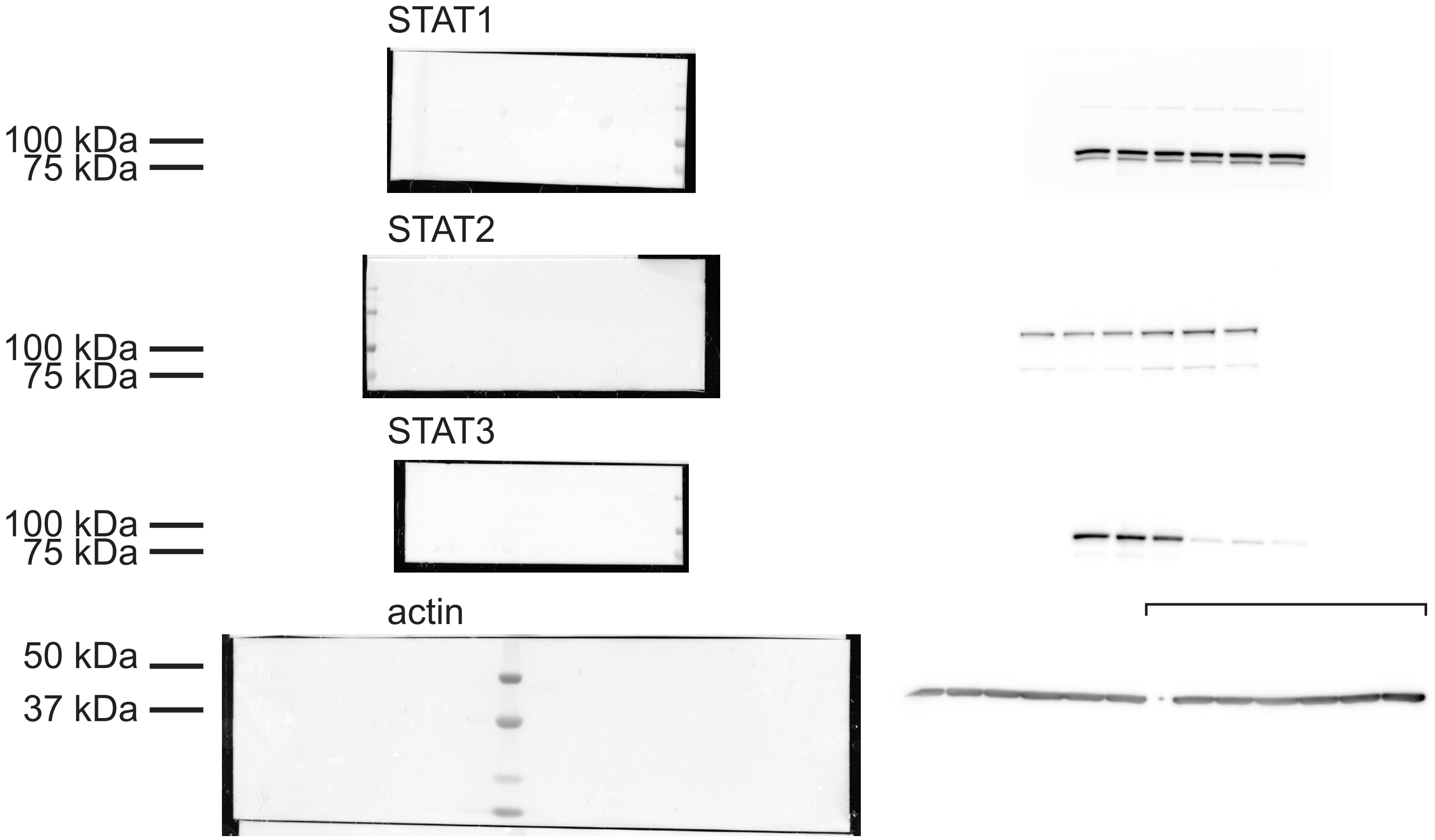

Uncropped blots corresponding to Figure 5E

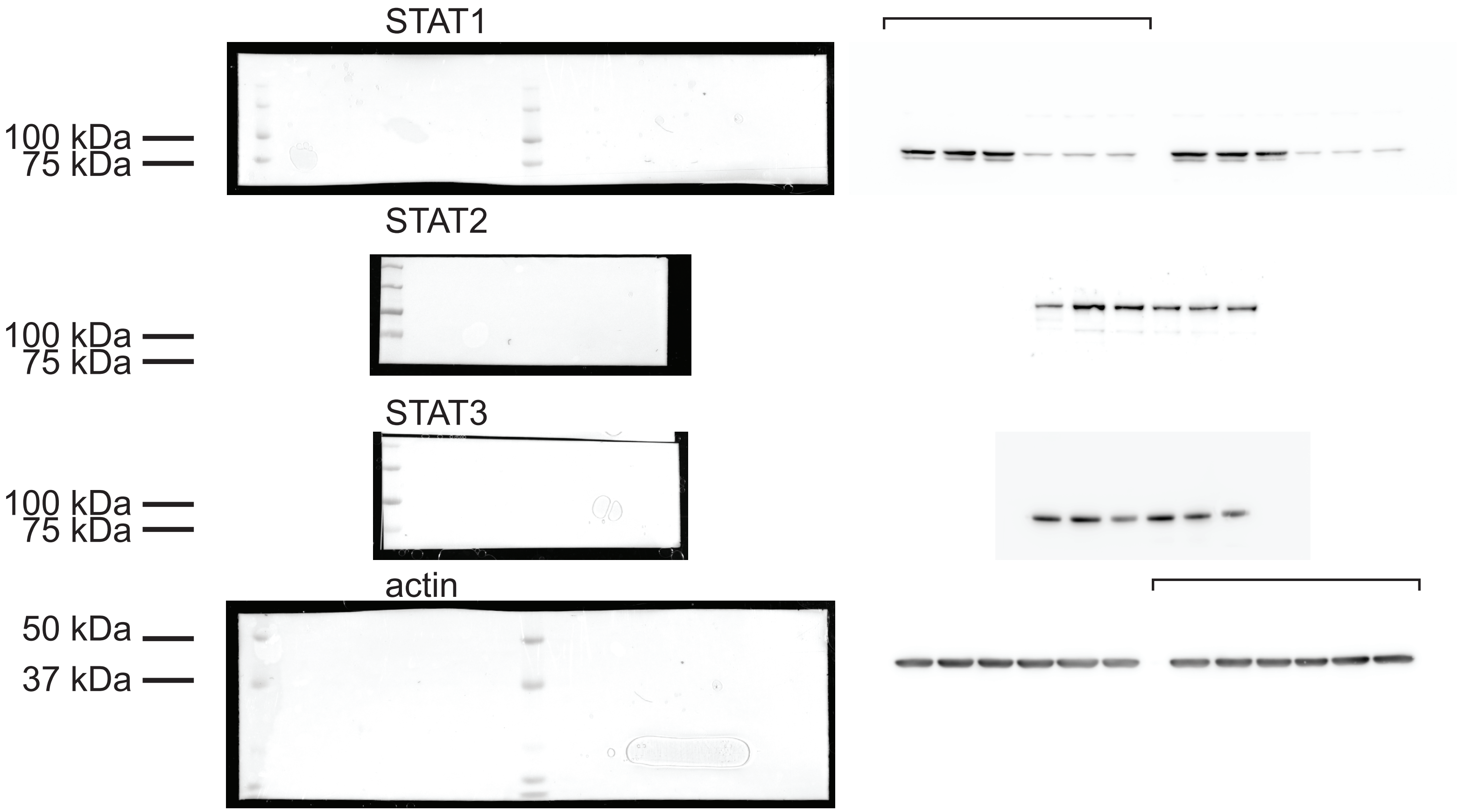

Uncropped blots corresponding to Figure 7B

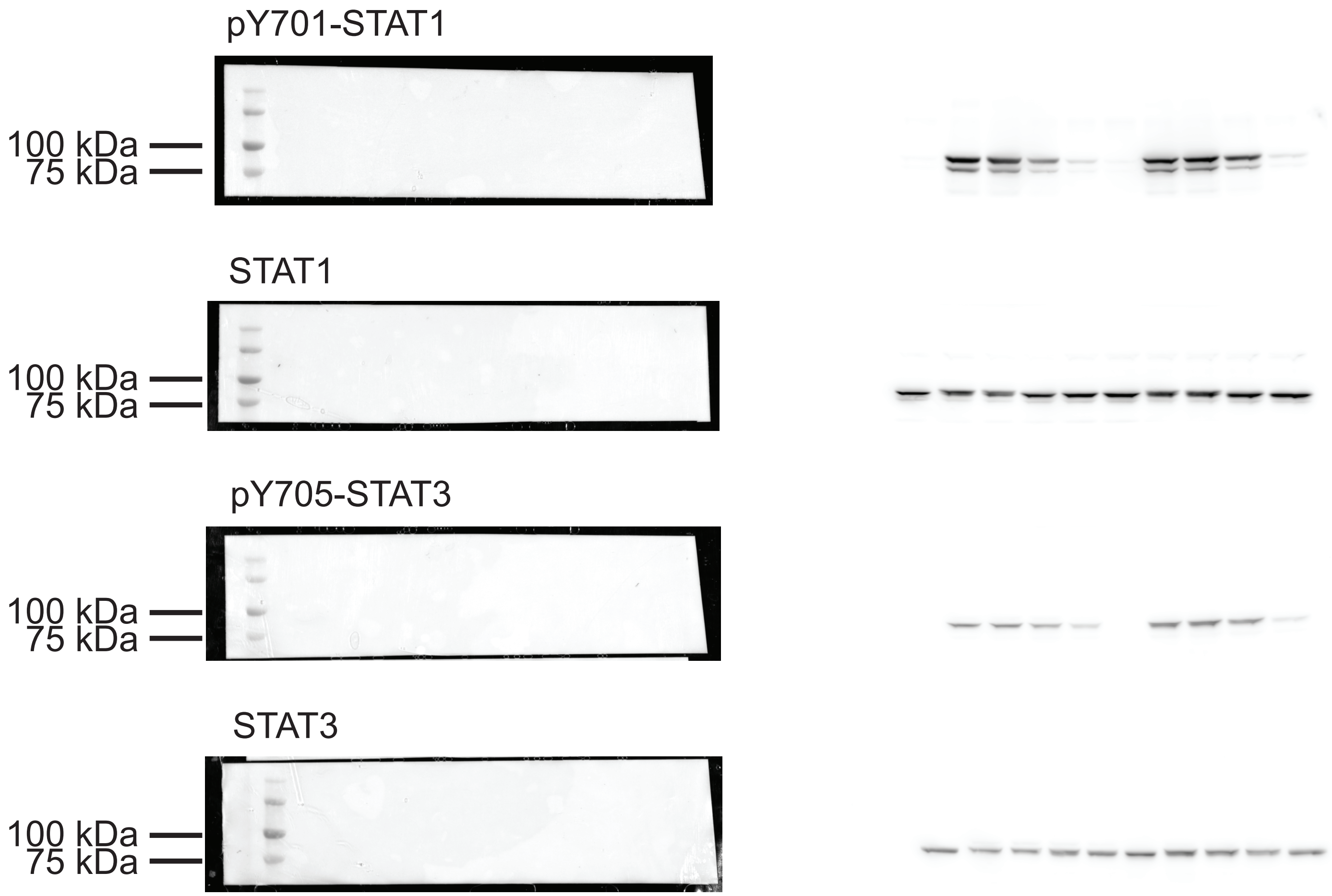

Uncropped blots corresponding to Figure 7C

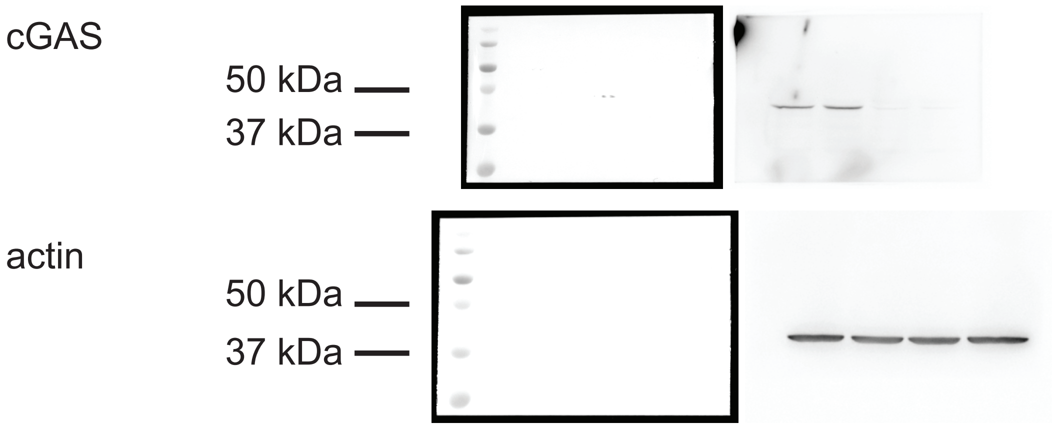

Uncropped blots corresponding to Figure 8A

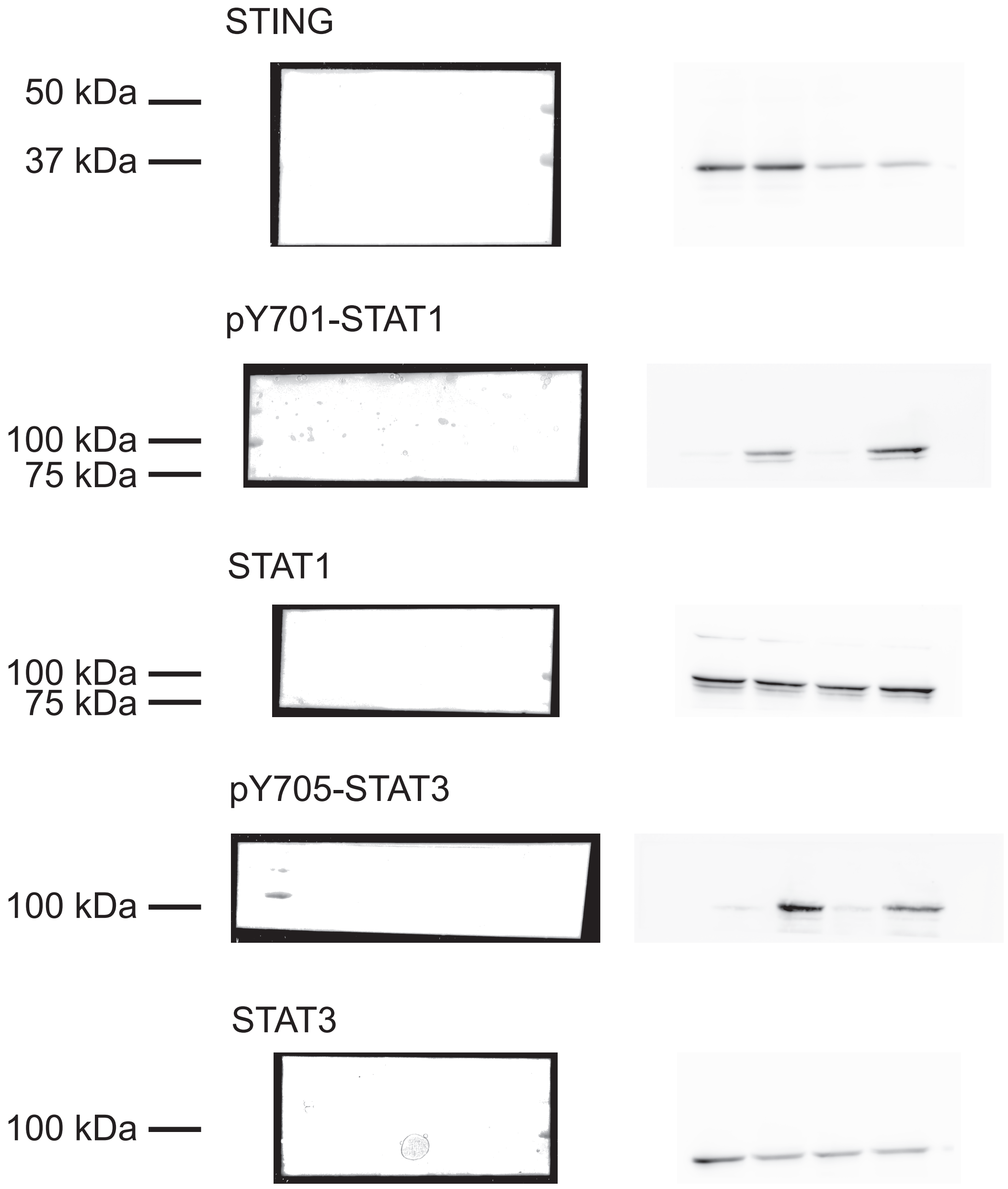

Uncropped blots corresponding to Figure 9A

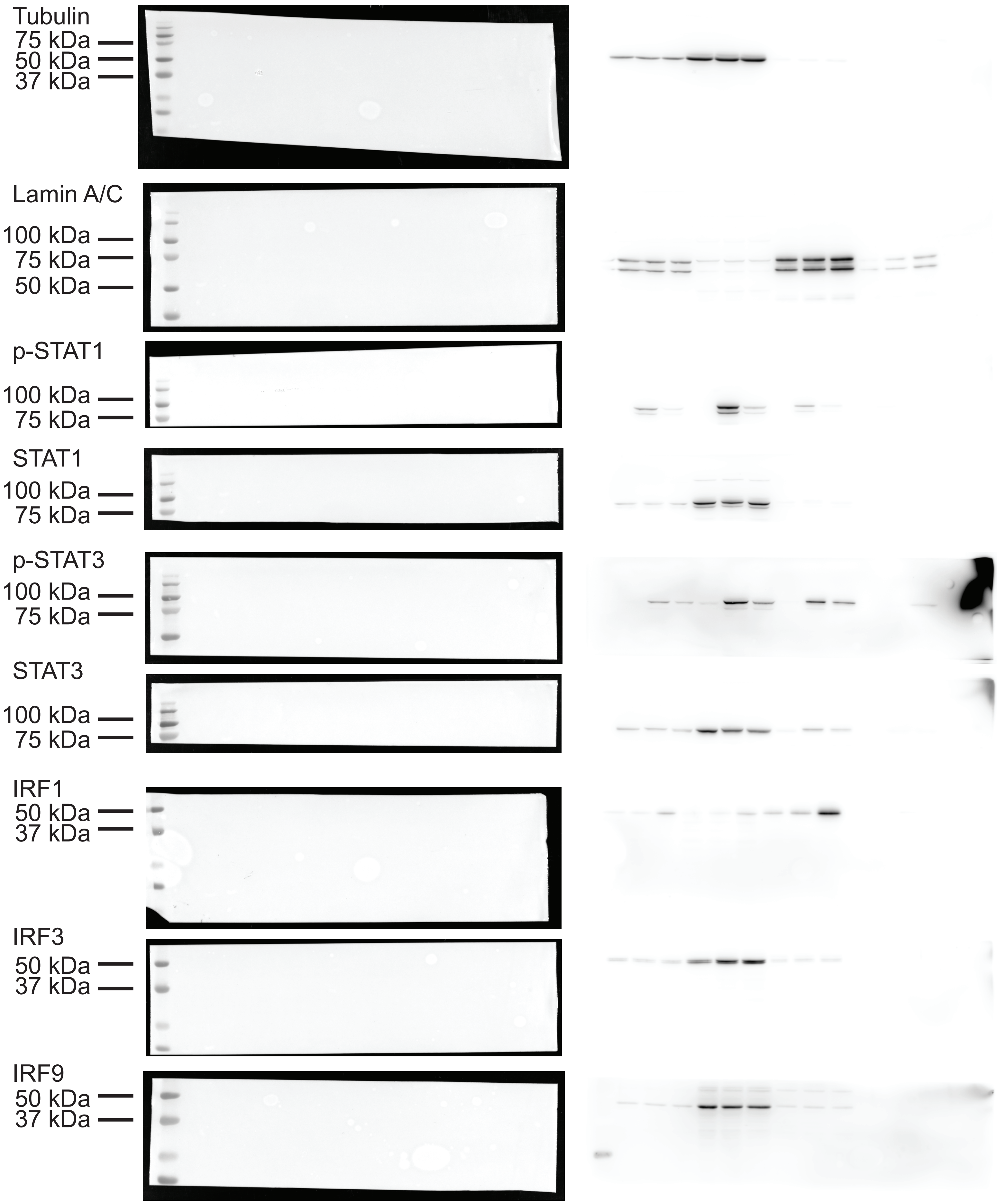

Uncropped blots corresponding to Figure S4B

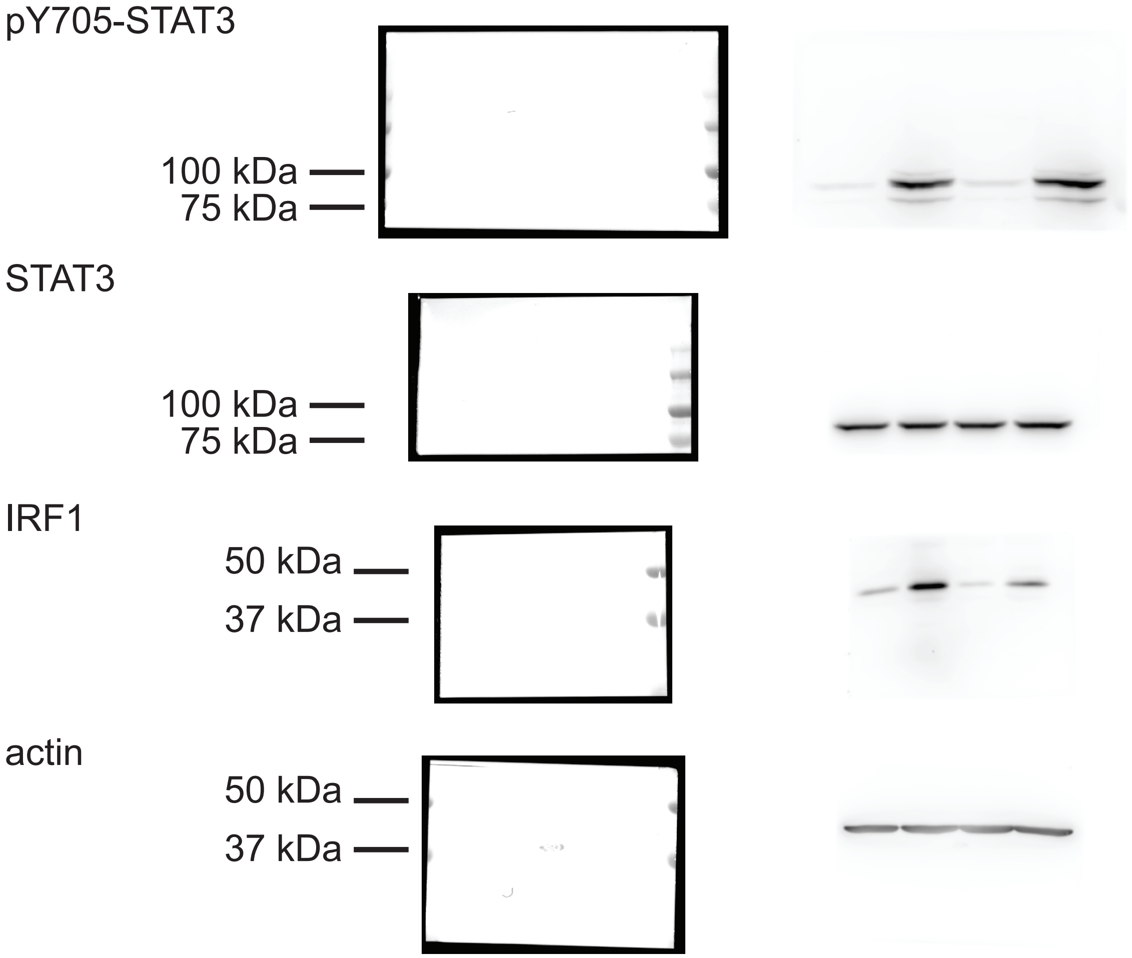

Supplement: Unedited blot and gel images [file jci-135-189570-s084.pdf]
